# New drug discovery of cardiac anti-arrhythmic drugs: insights in animal models

**DOI:** 10.1038/s41598-023-41942-4

**Published:** 2023-09-29

**Authors:** Ashish Kumar Sharma, Shivam Singh, Mehvish Bhat, Kartik Gill, Mohammad Zaid, Sachin Kumar, Anjali Shakya, Junaid Tantray, Divyamol Jose, Rashmi Gupta, Tsering Yangzom, Rajesh Kumar Sharma, Sanjay Kumar Sahu, Gulshan Rathore, Priyanka Chandolia, Mithilesh Singh, Anurag Mishra, Shobhit Raj, Archita Gupta, Mohit Agarwal, Sumaiya Kifayat, Anamika Gupta, Prashant Gupta, Ankit Vashist, Parth Vaibhav, Nancy Kathuria, Vipin Yadav, Ravindra Pal Singh, Arun Garg

**Affiliations:** 1 NIMS Institute of Pharmacy, NIMS University Rajasthan, Jaipur, Rajasthan 303121 India; 2 Medley Pharmaceuticals Ltd., Formulation R&D, Mumbai, 400059 India; 3 MVN University, Palwal, Haryana India

**Keywords:** Arrhythmias, Phenotypic screening

## Abstract

Cardiac rhythm regulated by micro-macroscopic structures of heart. Pacemaker abnormalities or disruptions in electrical conduction, lead to arrhythmic disorders may be benign, typical, threatening, ultimately fatal, occurs in clinical practice, patients on digitalis, anaesthesia or acute myocardial infarction. Both traditional and genetic animal models are: In-vitro: Isolated ventricular Myocytes, Guinea pig papillary muscles, Patch-Clamp Experiments, Porcine Atrial Myocytes, Guinea pig ventricular myocytes, Guinea pig papillary muscle: action potential and refractory period, Langendorff technique, Arrhythmia by acetylcholine or potassium. Acquired arrhythmia disorders: Transverse Aortic Constriction, Myocardial Ischemia, Complete Heart Block and AV Node Ablation, Chronic Tachypacing, Inflammation, Metabolic and Drug-Induced Arrhythmia. In-Vivo: Chemically induced arrhythmia: Aconitine antagonism, Digoxin-induced arrhythmia, Strophanthin/ouabain-induced arrhythmia, Adrenaline-induced arrhythmia, and Calcium-induced arrhythmia. Electrically induced arrhythmia: Ventricular fibrillation electrical threshold, Arrhythmia through programmed electrical stimulation, sudden coronary death in dogs, Exercise ventricular fibrillation. Genetic Arrhythmia: Channelopathies, Calcium Release Deficiency Syndrome, Long QT Syndrome, Short QT Syndrome, Brugada Syndrome. Genetic with Structural Heart Disease: Arrhythmogenic Right Ventricular Cardiomyopathy/Dysplasia, Dilated Cardiomyopathy, Hypertrophic Cardiomyopathy, Atrial Fibrillation, Sick Sinus Syndrome, Atrioventricular Block, Preexcitation Syndrome. Arrhythmia in Pluripotent Stem Cell Cardiomyocytes**.** Conclusion: Both traditional and genetic, experimental models of cardiac arrhythmias’ characteristics and significance help in development of new antiarrhythmic drugs.

## Introduction

Cardiovascular disease is the most common disease in the world today and has serious emotional and financial implications. Cardiac arrhythmias, affecting approximately 17 million people worldwide, are one of the most common types of heart disease and one of the leading causes of death. An irregular heartbeat is the hallmark of a condition called an abnormal heart rhythm. Atypical shock wave onset, abnormal shock wave propagation, or any combination thereof is associated with arrhythmias. Cardiac arrhythmias can manifest themselves in many ways and how exactly they work is not clear. Arrhythmias can also be classified based on heart rate^1^.

Cardiac arrhythmias affect ≈2% of community-dwelling adults, with an incidence of ≈0.5% per year^2^. Arrhythmias can manifest as relatively benign entities, such as atrial and ventricular premature beats, or as life-threatening arrhythmias such as ventricular tachycardia (VT) and ventricular fibrillation (VF), which can lead to sudden cardiac death (SCD), accounting for 10–15% of all deaths. Atrial fibrillation (AF) accounts for the greatest arrhythmia burden and is associated with stroke and heart failure, fueling huge health care costs. Arrhythmia treatment approaches focused on risk factor reduction, drug therapy, catheter ablation, device implantation, or a combination of these strategies has improved morbidity and mortality over the last 20 years, but treatment with antiarrhythmic drugs is often ineffective or increases mortality long term^3–5^. A more thorough understanding of the pathophysiology of arrhythmia initiation and maintenance is important for improving clinical outcomes. The mechanisms underlying arrhythmogenesis at the cellular level involve ion channels and electrogenic transporters that are altered via biogenic (synthesis, processing, trafficking, and degradation), biochemical (posttranslation modification, phosphorylation), and biophysical (gating, permeation) processes (reviewed here in study by Delisle et al.)^6^. The interplay between ion channels and transporters controls the action potential duration (APD), effective refractory period, and Ca2 + cycling to coordinate excitation–contraction coupling and normal myocyte function; dysregulation leads to abnormal cardiomyocyte electrical activity^7^. Structural and hemodynamic parameters contribute to further cardiac remodeling, increasing the risk for arrhythmia development and maintenance^8–11^. To study underlying arrhythmia mechanisms and evaluate treatment approaches, multiple in vitro systems and in vivo models have been developed. This review focuses on animal models that have informed our understanding of arrhythmia pathophysiology and have been used to develop new therapeutic approaches. An ideal model would recapitulate human anatomic, electrophysiological, and hemodynamic parameters. Currently, no single model can accomplish this feat. However, animal models have enabled the discovery of new treatment strategies for humans with genetic arrhythmia disorders. For example, mouse models demonstrated the efficacy of flecainide in catecholaminergic polymorphic ventricular tachycardia^12^ and mexiletine in long QT type 3^13^. When choosing an animal model of cardiac arrhythmia, researchers must consider the most appropriate model to address a specific scientific question based on cost, complexity, ease of handling, access to diagnostic and surgical expertise, and the ability for genetic modification.

Here, we provide the reader with a brief overview of basic and clinical cardiac electrophysiology, followed by an in-depth review of existing animal models of cardiac arrhythmias. Animal models are classified as either genetic (ie, arrhythmia risk caused by gene mutation) or acquired (ie, arrhythmia risk caused by nongenetic heart diseases such as myocardial infarction, metabolic abnormalities, or cardiac hypertrophy). Here, we provide the reader with a brief overview of basic and clinical cardiac electrophysiology, followed by an in-depth review of existing animal models of cardiac arrhythmias. Animal models are classified as either acquired (ie, arrhythmia risk caused by nongenetic heart diseases such as myocardial infarction, metabolic abnormalities, or cardiac hypertrophy) or genetic (ie, arrhythmia risk caused by gene mutation).

## Principles of cardiac electrophysiology

**Figure 1:**
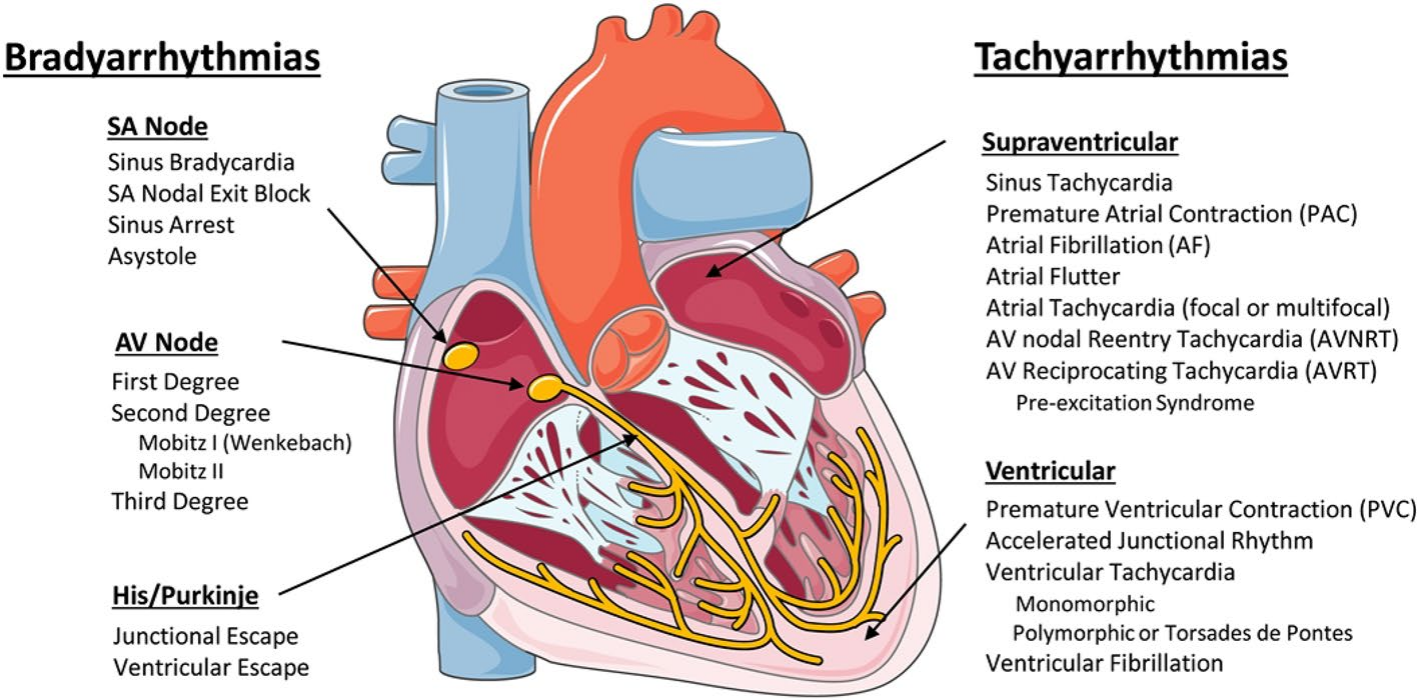
Schematic of the cardiac conduction system and clinical classification of cardiac arrhythmias. AF indicates atrial fibrillation; AV, atrioventricular; AVNRT, AV nodal reentry tachycardia; AVRT, AV reciprocating tachycardia; PAC, premature atrial; and SA, sinoatrial. Illustration credit: Ben Smith. [Daniel J. Blackwell. Circulation Research. Animal Models to Study Cardiac Arrhythmias, Volume: 130, Issue: 12, Pages: 1926–1964, (10.1161/CIRCRESAHA.122.320258)].

**Figure 2:**
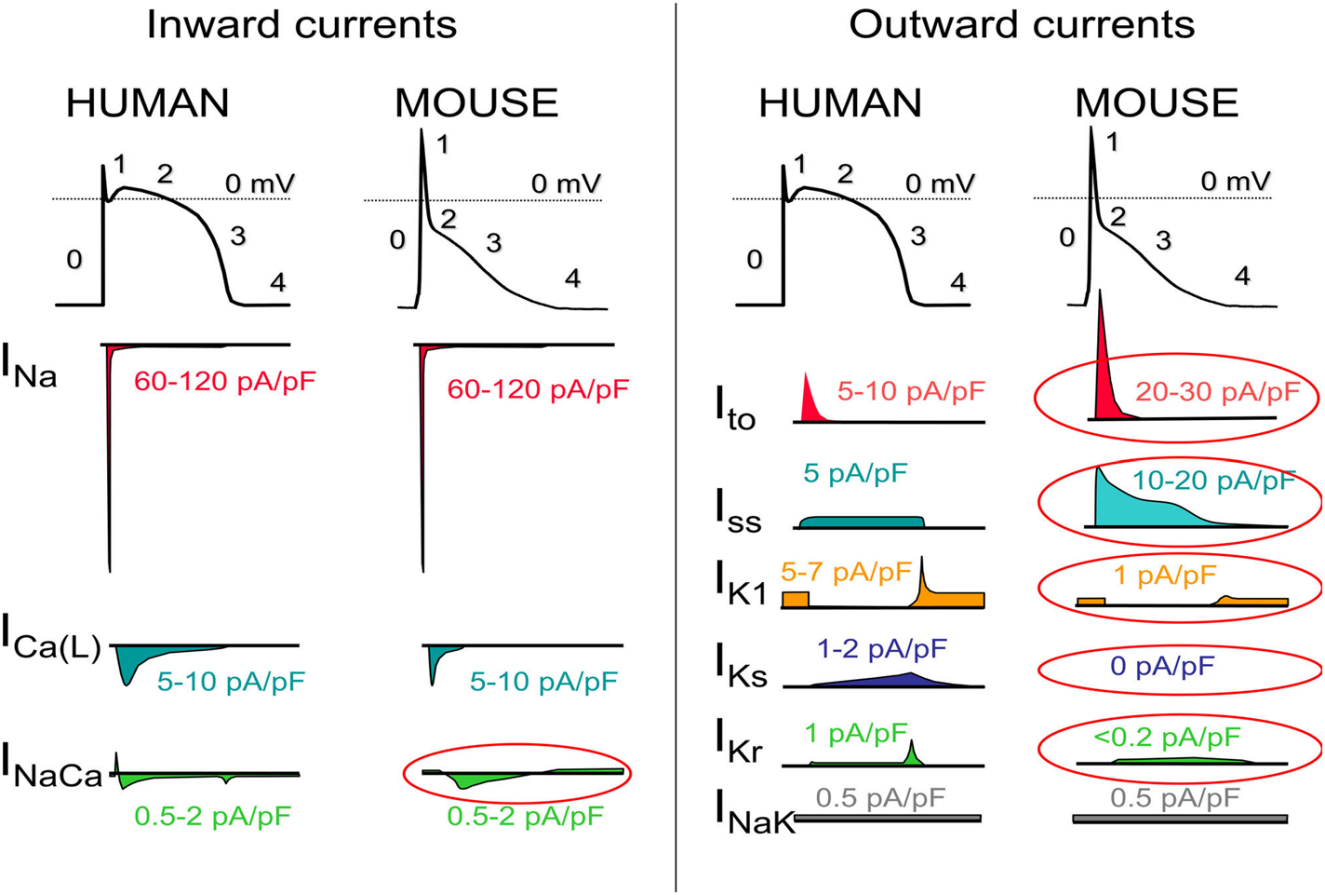
The ventricular action potential and ionic currents in humans and mice. Note the differences in action potential shape, which is caused primarily by differences in ionic currents circled in red. I_Ca(L)_ indicates L-type Ca current; I_Ca(T)_, T-type Ca current; I_Na_, Na current; I_NaCa_, Na-Ca-Exchange current; I_NaK_, NaK-ATPase pump current; I_K1_, Inward rectifier K-current; I_Kr_, rapidly activating delayed rectifier K-current; I_Ks_, slowly activating delayed rectifier K-current; I_ss_, rapidly activating steady-state K-currents; and I_to_, transient outward K-current. Please note that current densities (pA/pF) measured in single cells vary drastically with experimental conditions and voltage clamp protocols. Current densities were chosen to reflect relative contributions to the AP. [Daniel J. Blackwell. Circulation Research. Animal Models to Study Cardiac Arrhythmias, Volume: 130, Issue: 12, Pages: 1926–1964 (10.1161/CIRCRESAHA.122.320258)].

The Cardiac Conduction System Normal heart rhythm is generated and regulated in the specialized cardiac conduction system, which consists of the sinoatrial node, the atrioventricular (AV) node, and the HIS-Purkinje system (Fig. 1). Electrical impulses are initiated in the sinoatrial node and spread through the atria to the AV node. After a slight delay (0.12–0.20 s), excitation continues through the bundle of His, the right and left bundle branches, and finally the Purkinje fibers, which then excite the working myocardium. The delay in the AV node allows the atria to contract earlier than the ventricles and provides adequate time for optimal ventricular filling^14^. The specialized cells within the sinoatrial node, AV node, and His-Purkinje system are capable of spontaneous depolarization that is regulated by both the sympathetic and parasympathetic nervous system. Conduction through the heart depends on electrical coupling between cells, which is mediated by gap junctions. Species Differences in the Cardiac Action Potential and Cardiac Ca2 + Handling The cardiac action potential (AP) results from the opening and closing of ion channels and electrogenic transporters in the plasma membrane of individual cardiomyocytes (see study by Varró et al. for details)^15^. Figure 2 illustrates AP wave forms and underlying membrane currents for ventricular cardiomyocytes of humans and mice. When choosing an animal model for arrhythmia research, it is important to recognize species differences in cardiac AP and membrane currents, which are the result of species-specific expression of ion channels and transporters. For example, unlike humans, mice and rats have a low AP plateau at ≈ − 40 mV membrane potential (Fig. 2). This is primarily the result of differential expression in repolarizing transient K-currents, as illustrated in Fig. 2. On the other hand, rabbits and guinea pigs have a more positive AP plateau analogous to humans^16^. For a more detailed comparison of ionic currents in different species, the reader is referred to here^17^. As with the AP, there are important species differences in cardiac Ca2 + handling. For example, mice and rats primarily (> 90%) utilize sarcoplasmic reticulum (SR)- mediated Ca2 + cycling (via the cardiac ryanodine receptor [RyR2] and the SR Ca uptake pump [SERCA2a]) for excitation–contraction coupling, whereas in humans, dogs, and rabbits the SR accounts for ≈65%, with the remainder coming from outside the cell via the L-type Ca channel (CaV1.2) and the NCX (Na/Ca exchanger)^18^. For a more detailed comparison of species differences in Ca2 + handling, the reader is referred to here^19^. Pathophysiology of Cardiac Arrhythmias The main mechanisms of arrhythmogenesis can be divided into either abnormal impulse generation or abnormal impulse propagation. Disorders of impulse generation and propagation, regulation of the AP duration, and cellular substrates can all contribute to 3 categories of arrhythmias; enhanced automaticity, triggered ectopic beats, and reentry. Each arrhythmia category is explained briefly below. A more detailed review can be found in here. Automaticity Automaticity is the ability of cells to generate their own AP^20^. The intrinsic depolarization rate of the sinoatrial node is faster than the rest of the cardiac conduction system and overdrives pacemaking in the AV node and His-Purkinje system. However, automaticity in the AV node and His-Purkinje system can become dominant in sinoatrial nodal dysfunction. The sinoatrial node is more sensitive to increased sympathetic and parasympathetic tone, leading to sinus tachycardia and bradycardia, respectively. Under normal conditions, atrial and ventricular cardiomyocytes display either no or very slow intrinsic depolarization that are easily suppressed by the faster, coordinated impulses from the sinoatrial node through the conduction system. Increased automaticity in the atria can lead to focal and multifocal atrial tachycardia and AF. Specifically, the pulmonary vein sleeve, where the left atria myocytes transition to the tunica media of the pulmonary veins, is known to harbor tissue with increased automaticity^21^, and is a target for catheter based ablation by pulmonary vein isolation for AT and AF. Increased automaticity in the ventricle is less common but can lead to VT or accelerated idioventricular rhythms. Afterdepolarizations and Triggered Arrhythmia Triggered arrhythmias are because of spontaneous membrane depolarization of atrial or ventricular myocytes that precede the next sinus beat. Membrane depolarizations that occur within or follow the cardiac AP are referred to as afterdepolarizations. Two classes are traditionally recognized: early and delayed. An early afterdepolarization (EAD) interrupts the repolarization during phase 2 or early phase 3 of the cardiac AP, whereas a delayed afterdepolarization (DAD) occurs after full repolarization in Phase 4. When an EAD or DAD brings the membrane to its threshold potential, a spontaneous AP is referred to as a triggered response. These triggered events can give rise to premature extrasystolic complexes in the atria or the ventricle (PVCs), precipitating tachyarrhythmias. In general, any unbalanced increased inward current (ie, gain-of-function mutations in Na + or Ca2 + channels) or decreased outward currents (ie, loss-of-function mutations in K + channels) will depolarize the cell membrane and can lead to EADs or DADs^22^. Specifically, a major cause of triggered arrhythmia is spontaneous RyR2-mediated SR Ca2 + release, driving inward Na + current via the NCX, leading to EAD and in particular DAD formation, which are important cellular arrhythmia mechanisms in AF, VT, and SCD^23^. Reentrant Arrhythmia In reentry, a group of myocardial cells that are not activated during the early stage of depolarization can resume excitability before the impulse vanishes. In this situation, they may connect to re-excite zones that were previously depolarized but were recovered from the refractory period of the initial wave. Two crucial factors predisposing reentry are prolonged conduction time and shortened refractory period. Reentry is the dominant mechanism of arrhythmias in the clinical setting and occurs due to anatomic and functional factors^24^.

### Classification of clinical arrhythmias

**Figure 3:**
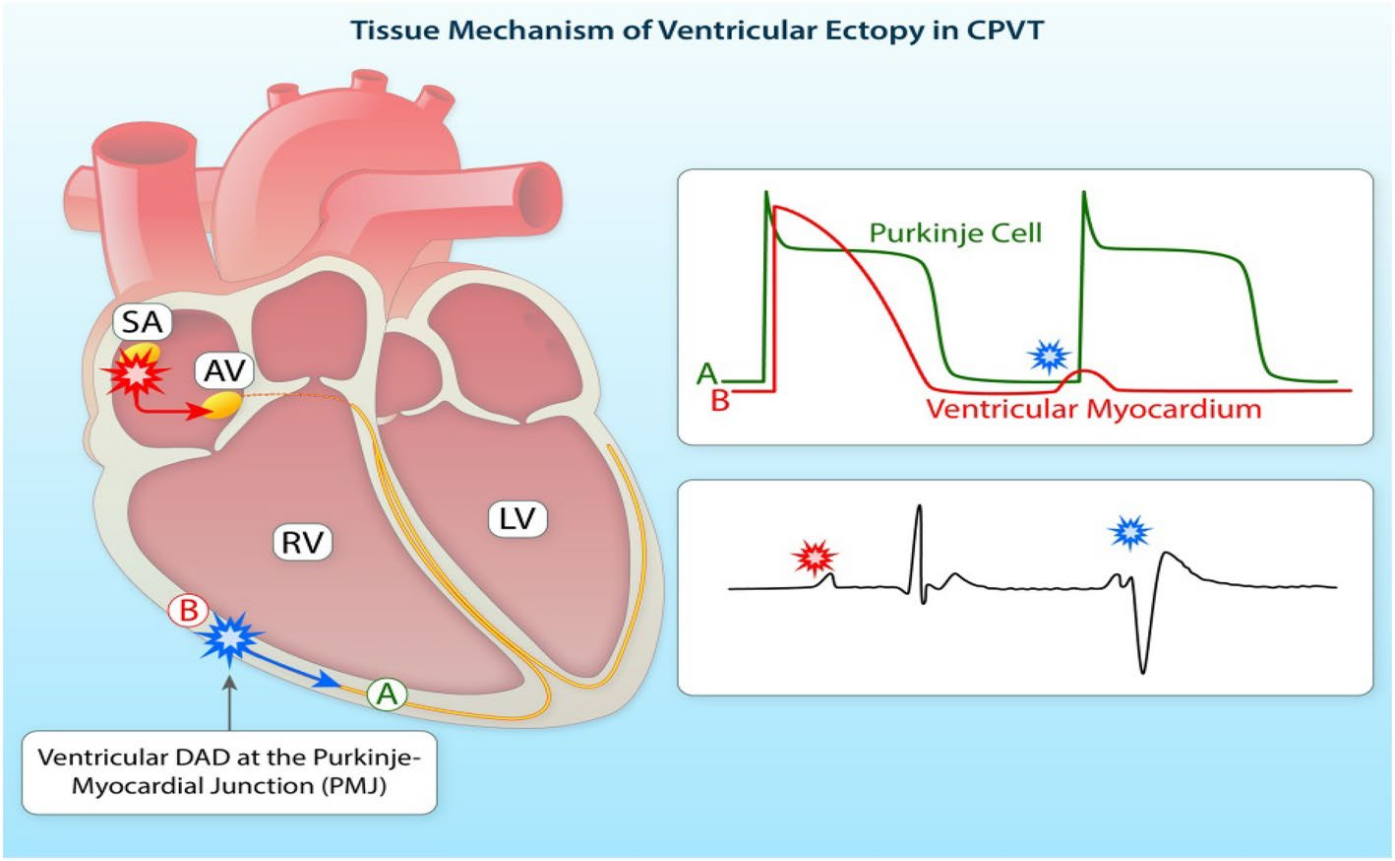
Tissue-targeted CASQ2 knock-out mice help decipher the anatomic origin of ventricular ectopy in catecholaminergic polymorphic ventricular tachycardia (CPVT). Based on a combination of tissue-targeting and in silico modeling, the PMJ was identified as the likely origin of ventricular ectopy in CPVT. DAD indicates delayed afterdepolarization; LV, left ventricular; PMJ, Purkinje myocardial junction; and RV, right ventricular. Illustration credit: Ben Smith. [Daniel J. Blackwell. Circulation Research. Animal Models to Study Cardiac Arrhythmias, Volume: 130, Issue: 12, Pages: 1926–1964 (10.1161/CIRCRESAHA.122.320258)].

Clinically, cardiac arrhythmias are usually classified as bradyarrhythmias and tachyarrhythmias (Fig. 1). Both types can reduce cardiac output, resulting in hypotension and ultimately can cause death but have different underlying mechanisms. Figure 1 lists the major clinical types of brady and tachyarrhythmias. Briefly, bradyarrhythmias reduce the heart rate either by reducing spontaneous depolarization within the sinoatrial node, slowing conduction through the conduction system, or increasing parasympathetic tone. Sinus bradycardia, sinus node exit block, sinus arrest, and asystole are caused by dysfunction within the sinus node itself, due to destruction of the pacemaker cells, fibrosis of the sinoatrial node, or increased parasympathetic tone. AV nodal block prolongs the conduction above, within or below the AV node. Depending on the severity of AV block, it is classified as first-degree, second-degree, or complete (third-degree) heart block (Fig. 1). Tachyarrhythmias are accelerated rhythms that originate from either above (supraventricular tachycardia) or below the AV node (ventricular arrhythmia). The most common supraventricular tachycardias are sinus tachycardia and AF. Premature atrial contractions and AT are commonly caused by automatic foci within the atria. Reentrant atrial arrhythmias include atrial flutter, AV nodal reentry tachycardia, and AV reciprocating tachycardia. Atrial flutter is a macroreentrant loop, typically involving the tricuspid annulus limited by anatomic barriers such as the superior and inferior cava veins, the coronary sinus and crista terminalis^25^. AV nodal reentry tachycardia is a microreentry related to differences in the refractory period of the slow and fast pathway within the AV node^26^. AV reciprocating tachycardia, also known as preexcitation syndrome, occurs due to the presence of an accessory pathway, most notably the Bundle of Kent leading to Wolf-Parkinson-White syndrome, which can prematurely conduct impulses between the atria and ventricles. Ventricular arrhythmias include premature ventricular contractions (PVCs), VT, and VF. PVCs are single premature beats due to EADs or DADs in myocardial cells and benign, unless they trigger VT or VF. VT and VF are usually reentrant arrhythmias, and if not treated rapidly, can lead to sudden cardiac death. While a majority of cases of VT are because of reentry around the scar in structural heart disease, 10% of VT occurs in structurally normal hearts due to nonreentrant mechanisms such as catecholaminergic polymorphic VT (CPVT), fascicular VT, left or right outflow tract VT, mitral and tricuspid annular VT, long QT, and Brugada syndrome^27^. Animal Models An important consideration for selecting an animal model to study cardiac arrhythmias is how closely the species resembles human cardiac physiology. Caenorhabditis elegans and Drosophila melanogaster both develop heart tubes and have been primarily used to screen gene function and examine development and cardiac structure. Zebrafish have a 2 chambered heart with some similarities in AP electrophysiology to humans and provide advantages for understanding cardiogenesis. Zebrafish embryos are transparent, enabling optical viewing, fluorescent protein expression, and optogenetic pacing; they have large clutch sizes with a rapid embryonic stage lasting only 3 to 4 days postfertilization; are amenable to drug absorption; and genes are easily manipulated. Mouse hearts are anatomically similar to human hearts with 4 chambers and comparable development^28^, albeit differences in coronary anatomy^29^. However, there are major differences in heart rate, cardiac AP, and membrane currents (Fig. 2). These differences influence ion channel function, refractoriness, and arrhythmia susceptibility. In addition, the small size of the mouse heart may contribute to the frequently observed self-termination of reentrant arrhythmias or lack of spontaneous arrhythmias in many models. Nevertheless, the mouse has been the primary animal model for cardiac arrhythmia studies of inherited cardiomyopathies and channelopathies, and many models faithfully capture cardiac disease. Rabbits more closely recapitulate the human AP compared with rats and mice. The rabbit AP has a sustained Ca2 + current-driven plateau phase, and the major repolarizing K + currents are similar to humans. Rabbit heart size and beating rate is between that of mice and humans. Dogs, pigs, and goats have a similar cardiac anatomy, size, and beating rate (slightly higher in dogs) as humans. Their cardiac electrophysiology, APs, and ionic currents are all fairly comparable to humans, and their primary limitations as an animal model for research come from their cost, size, and time to breed and reach sexual maturity (Fig. 3).

## Methods for antiarrhythmic drug screening (animal models)

### Cell culture technique

#### Studies on isolated ventricular myocytes

Isolated ventricular myocytes can be used to assess ventricular arrhythmias, particularly torsades de pointes. Action potential analysis and patch-clamp methods in isolated ventricular myocytes can help us better understand the mechanisms underlying torsades de pointes.

Proarrhythmia has been observed with the antipsychotic agent thioridazine (THIO). The mechanisms underlying these effects are unknown. The objectives of this study were 1) to characterize the effects of THIO on cardiac repolarization and 2) to determine whether lengthening of the Q-T interval could be explained by blocking major K + -repolarizing currents. Isolated, buffer-perfused guinea pig hearts (n = 32) were stimulated at various pacing cycle lengths (150–250 ms) and exposed to THIO at concentrations ranging from 300 nM to 3 microM. THIO increased monophasic action potential duration at 90% repolarization (MAPD90) in a concentration-dependent manner from 14.9 + /- 1.8 at 300 nM to 37.1 + /- 3.2 ms at 3 microM. Increase in MAPD90 was also reverse frequency-dependent; THIO (300 nM) increased MAPD90 by 14.9 + /- 1.8 ms at a pacing cycle length of 250 ms, but by only 7.7 + /- 1.2 ms at a pacing cycle length of 150 ms. Patch-clamp experiments demonstrated that THIO decreases the time-dependent outward K + current elicited by short depolarizations (250 ms; IK250) in a concentration-dependent manner. Estimated IC50 for IK250, which mostly underlies IKr, was 1.25 microM. Time-dependent outward K + current elicited in tsA201 cells expressing high levels of HERG protein was also decreased approximately 50% by 1.25 microM THIO. On the other hand, THIO was less potent (IC50 of 14 microM) to decrease time-dependent K + current elicited by long pulses (5000 ms; IK5000). Under the latter conditions, IK5000 corresponds mainly to IKs. Thus, these results demonstrate block of K + currents and lengthening of cardiac repolarization by THIO in a concentration-dependent manner. This may provide an explanation of Q-T prolongation observed in some patients treated with THIO^30^.

Limitations of Isolated ventricular myocytes used to assess ventricular arrhythmias, particularly torsades de pointes. We can better understand the processes causing torsades de pointes by analysing action potential and patch clamp techniques in isolated ventricular myocytes but not Atrial Fibrillation-Flutter Arrhythmias.

### In-vitro models

#### Isolated guinea pig papillary muscles

A fast and accurate method without microelectrodes is available for identifying and classifying potential antiarrhythmic drugs into Classes I, II, III and IV. In the guinea pig right ventricle, papillary muscle excitability, developed tone (DT) and effective refractory period (ERP) are evaluated. Inhibition of the Na + channel decreases excitability, inhibition of the K + channel increases refractory period, and blockade of the Ca2 + channel decreases myocardial tone.

Class I antiarrhythmic agents are heterogeneous with respect to their cardiac electrophysiological effects and have been subdivided into three categories: la, lb and lc. The purpose of the present study was to determine the classification and investigate the mechanism of action of ACC-9358 [4-hydroxy-N-phenyl-3,5-bis (1-pyrrolidinyl-methyl)benzamide], a novel class I antiarrhythmic agent currently under clinical investigation. The effects of ACC-9358 on action potentials from isolated canine Purkinje fibers and ventricular muscle were examined using standard microelectrode techniques. In Purkinje fibers, ACC-9358 (1–50 microM) exerted a dose-dependent reduction in maximum upstroke velocity (Vmax) and action potential duration at 50 and 90% repolarization (APD50 and APD90). The reduction of Vmax was voltage-dependent (greater at an extracellular potassium concentration of 6 mM than at 2.7 mM), frequency-dependent (greater at a basic cycle length of 500 than at 2000 ms) and very slow in onset (rate constant of 0.017 action potentials-1) and offset (recovery half-time of 66.9 s). In Purkinje fibers, ACC-9358 attenuated the action potential shortening effects of lidocaine but not that of nicardipine or nicorandil and shortened APD50 to a greater extent at a basic cycle length of 2000 than at 500 ms. In ventricular muscle, ACC-9358 (1–50 microM) exerted a dose-dependent reduction in Vmax and prolongation of APD50 and APD90^31^.

**Figure 4:**
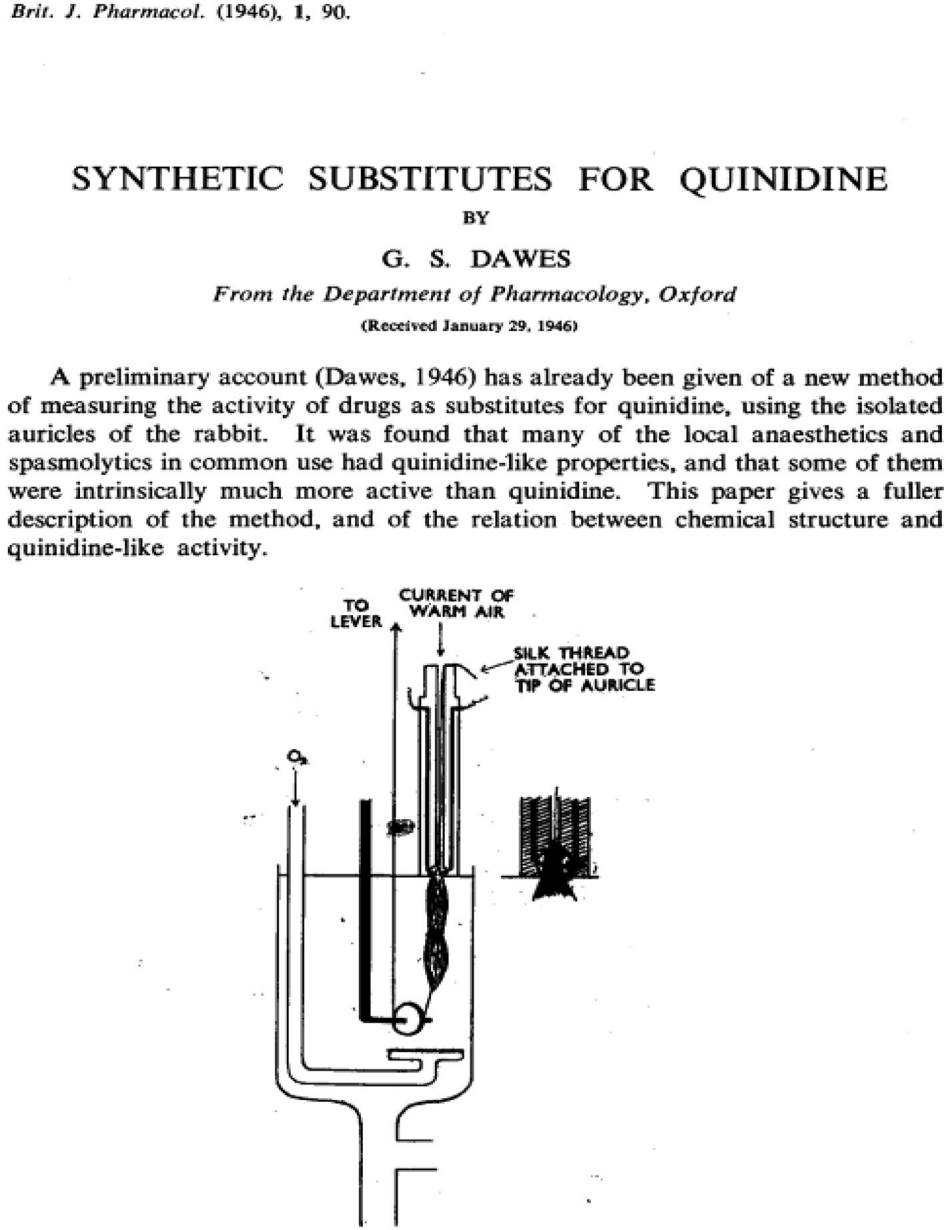
Prepration of rabbit auricles suspended in oxygenated Ringer-Locke at 29 °C. The electrode holder is made of perpex, and the inset shows a closer view of its lower end. (Dawes GS. Synthetic substitutes for quinidine. British Journal of Pharmacology and Chemotherapy. 1946 Jun;1(2):90–112. PMCID: PMC1509732 PMID: 19108083).

Figure 4 is a diagrammatic illustration of the preparation. The auricles are dissected from the heart of a rabbit and suspended in a bath of oxygenated Ringer-Locke at 29 °C.; at their upper end they are fixed in a pair of platinum electrodes just above the surface of the bath. The sharpened tips of the electrodes project into a tiny chamber at the bottom of a perspex rod. This chamber tapers at its lower end to form an oval opening; a silk thread is tied through the tip of one auricle, which is then drawn into the chamber so that the electrodes penetrate its substance, and so that the auricle itself completely seals the oval opening. At its upper end the chamber is continuous with a circular channel which runs through the perspex rod; a gentle current of warm air is blown down this channel at constant pressure, and serves the double purpose of oxygenating the tissue in the chamber and of preventing Ringer-Locke entering the chamber by capillary attraction, and so shortcircuiting the electrodes. The object of this device is to ensure that, while the main part of the muscle is immersed in the bath, the electrodes are outside it; when methyl violet was added to the bath, the tip of the auricle drawn up into the electrode chamber was scarcely stained at all. The contraction of the muscle is recorded by a lever writing on a smoked drum, and attached to the lower end of the auricles by a silk thread running round a pulley immersed in the bath. The auricles contract spontaneously (at a rate of 80–120 per minute), and they can also be stimulated by break-shocks from an induction coil at any desired speed, using a Lewis rotary contact-breaker. The coil separation is adjusted so that the peak voltage in the secondary is about ten times that necessary to cause extrasystoles at the beginning of the experiment; this ensures that stimuli are so far above threshold that the notorious irregularity of induction shocks will not be of practical significance. As the rate of electrical stimulation is increased the auricle follows each stimulus up to a certain point (between 250 and 350 per minute) at which it begins to drop beats (because the interval between shocks is less than the absolute refractory period; cf. Mines, 1913). It is easy to distinguish these dropped beats since the next auricular contraction is more powerful. Thus in t experiment shown in Fig. 2 the auricle followed each stimulus at 200 and 260 per minute to 266 per minute it dropped a single beat, and at 274 per minute the response soon became ery irregular; at 338 per minute it had adopted a 2: 1 rhythm. In this instance the maximal rate at which the auricle could respond would be recorded as 260 per minute. The method is based upon the observation that quinidine reduces this maximal rate^32^.

The maximal rate at which the auricle responds to electrical stimuli is 218/min.; after quinidine 1:100,000 for 10 min this is reduced to 184/min. i.e., the maximal rate was reduced by 34 per minute or 15.6 per cent^32^.

Aftercontractions, delayed afterdepolarizations, and automaticity occurred in guinea pig papillary muscles that were reoxygenated after hypoxic conditioning. The emergence of dysfunction was dependent on the severity of hypoxic conditioning and on stimulation during reoxygenation. After 60 min of substrate-free hypoxia, reoxygenation induced automaticity in a high proportion of stimu- lated muscles; the automaticity appeared within 1 min and lasted for 10–20 min. After similar conditioning, muscles reoxygenated for 7–15 min were stimulated at various cycle lengths. The incidence of automaticity and the amplitudes of delayed events had W-shaped dependencies on cycle length (200–1000 ms), whereas coupling intervals had M-shaped dependencies. In ventricular myocytes that displayed automaticity after reoxygenation, extrasystolic upstrokes arose smoothly from delayed afterdepolarizations that reached threshold. In tissue, extrasystolic upstrokes usually rose sharply from delayed afterdepolarizations that were distinctly subthreshold. Thus, threshold was reached elsewhere in the tissue. Further evidence of electrical heterogeneity was obtained from surface mapping of delayed-afterdepolarization amplitude in reoxygenated muscle. There were no detectable aftercontractions, delayed afterdepolarizations, or signs of automaticity in quiescent reoxy- genated muscles or in stimulated reoxygenated muscles that were treated with 1/uM ryanodine. We conclude that the dysfunction precipitated by reoxygenation is due to synchronized spontaneous releases of calcium from overloaded sarcoplasmic reticulum^33^.

Limitations of Isolated guinea pig papillary muscles used to assess ventricular arrhythmias but not Atrial Fibrillation-Flutter & Torsades de Pointes Arrhythmias.

#### Patch-clamp experiments in CHO cells

Cells expressing hKv1.5 or hKv4.3 with hKChIP2.2b were assessed using the conventional whole cell patch clamp technique^34^.

The novel compound AVE1231 was investigated in order to elucidate its potential against atrial fibrillation. In CHO cells, the current generated by hKv1.5 or hKv4.3 + KChIP2.2b channels was blocked with IC50 values of 3.6 microM and 5.9 microM, respectively. In pig left atrial myocytes, a voltage-dependent outward current was blocked with an IC50 of 1.1 microM, mainly by accelerating the time constant of decay. Carbachol-activated IKACh was blocked by AVE1231 with an IC50 of 8.4 microM. Other ionic currents, like the IKr, IKs, IKATP, ICa, and INa were only mildly affected by 10 microM AVE1231. In guinea pig papillary muscle the APD90 and the upstroke velocity were not significantly altered by 30 microM AVE1231. In anesthetized pigs, oral doses of 0.3, 1, and 3 mg/kg AVE1231 caused a dose-dependent increase in left atrial refractoriness (LAERP), associated by inhibition of left atrial vulnerability to arrhythmia. There were no effects on the ECG intervals, ventricular monophasic action potentials, or ventricular refractory periods at 3 mg/kg AVE1231 applied intravenously. In conscious goats, both AVE1231 (3 mg/kg/h iv) and dofetilide (10 microg/kg/h iv) significantly prolonged LAERP. After 72 h of tachypacing, when LAERP was shortened significantly (electrical remodelling), the prolongation of LAERP induced by AVE1231 was even more pronounced than in sinus rhythm. In contrast, the effect of dofetilide was strongly decreased. The present data demonstrate that AVE1231 blocks early atrial K channels and prolongs atrial refractoriness with no effects on ECG intervals and ventricular repolarisation, suggesting that it is suited for the prevention of atrial fibrillation in patients^34^.

Limitations of Patch-Clamp Experiments in CHO Cells used to assess ventricular arrhythmias but not Atrial Fibrillation-Flutter & Torsades de Pointes Arrhythmias.

#### Isolation of porcine atrial myocytes

All investigations with animals conform to the Guide for the Care and Use of Laboratory Animals published by the US National Institutes of Health (NIH publication no. 85–13, revised 1996) and were performed following approval by the Ethical Review Board of the State of Hessen and in accordance with the German animal protection law.

Male pigs (German Landrace) weighing 15 to 30 kg were anesthetized with pentobarbital as described previously. After a left thoracotomy, the lung was retracted, the pericardium incised, and the heart quickly removed and placed in oxygenated, nominally Ca^2+^-free Tyrode solution containing (in mM): NaCl 143, KCl 5.4, MgCl_2_ 0.5, NaH_2_PO_4_ 0.25, HEPES 5, and glucose 10, pH adjusted to 7.2 with NaOH. The hearts were then mounted on a Langendorff apparatus and perfused via the left circumflex coronary artery with Tyrode solution at 37 °C with constant pressure (80 cm H_2_O). All coronary vessels descending to the ventricular walls were ligated, ensuring sufficient perfusion of the left atrium. When the atrium was clear of blood and contraction had ceased (∼5 min), perfusion was continued with the same Tyrode solution, which now contained 0.015 mM CaCl_2_ and 0.03% collagenase (type CLS II, Biochrom KG, Berlin, Germany), until atrial tissue softened (∼20 min). Thereafter, left atrial tissue was cut into small pieces and mechanically dissociated by trituration. Cells were then washed with storage solution containing (in mM): L-glutamic acid 50, KCl 40, taurine 20, KH_2_PO_4_ 20, MgCl_2_ 1, glucose 10, HEPES 10, EGTA 2 (pH 7.2 with KOH), and then filtered through a nylon mesh. The isolated cells were kept at room temperature in the storage solution^35^.

Limitations of Isolation of Porcine Atrial Myocytes used to assess Atrial Fibrillation-Flutter arrhythmias but not ventricular Arrhythmias.

Atrial fibrillation (AF) is the most common sustained arrhythmia encountered in humans and is a significant source of morbidity and mortality. Despite its prevalence, our mechanistic understanding is incomplete, the therapeutic options have limited efficacy, and are often fraught with risks. A better biological understanding of AF is needed to spearhead novel therapeutic avenues. Although “natural” AF is nearly nonexistent in most species, animal models have contributed significantly to our understanding of AF and some therapeutic options. However, the impediments of animal models are also apparent and stem largely from the differences in basic physiology as well as the complexities underlying human AF; these preclude the creation of a “perfect” animal model and have obviated the translation of animal findings. Herein, we review the vast array of AF models available, spanning the mouse heart (weighing 1/1000th of a human heart) to the horse heart (10 × heavier than the human heart). We attempt to highlight the features of each model that bring value to our understanding of AF but also the shortcomings and pitfalls. Finally, we borrowed the concept of a SWOT analysis from the business community (which stands for strengths, weaknesses, opportunities, and threats) and applied this introspective type of analysis to animal models for AF. We identify unmet needs and stress that is in the context of rapidly advancing technologies, these present opportunities for the future use of animal models^36^.

#### Isolation of guinea pig ventricular myocytes

Ventricular myocytes were isolated by enzymatic digestion according to the same procedure as above. We used 400 g Dunkin Hardley Pirbright White guinea pigs to kill them quickly by concussion and cervical dislocation. The chest was opened; the hearts removed and immediately placed in ice-cold isotonic saline. Retrograde cardiac perfusion through the aorta at 37 °C was performed with the same solutions used for the isolation of porcine atrial myocytes^37^.

Limitations of Isolation of Guinea Pig Ventricular Myocytes used to assess ventricular arrhythmias but not Atrial Fibrillation-Flutter arrhythmias.

#### Isolated guinea pig papillary muscle: action potential and refractory period

After electrical stimulation, the papillary muscle of the left ventricle of a guinea pig registers an intracellular action potential. The stimulation frequency is changed to determine the refractory period. The pro- or anti-arrhythmic effect of a chemical can be identified by the length of the effective refractory period.The inotropic effect of the test substance is also determined.

The electromechanical effects of UK-68,798 (UK), a novel class III antiarrhythmic drug, were studied in guinea pig and rat papillary muscles (PMs) and atria in vitro using conventional microelectrode technique. UK (10(-8)-10(-6) M) prolonged the action potential duration (APD) by 21–58% and effective refractory period in parallel, without affecting the resting potential or maximum rate of depolarization in guinea pig PM stimulated at 1 Hz. UK increased the contractile force without prolonging the time to peak force or relaxation. In comparison, 5 × 10(-5) M d-sotalol was needed to induce the same electrophysiological effects as 10(-8) M UK. UK prolonged the APD significantly less at 2 Hz than at 1 and 0.5 Hz. Early afterdepolarizations (EADs) developed in 2 of 11 preparations after 10(-6) M at 0.5 Hz. No reversal of drug effect was seen after up to 2 h washout. UK (10(-9)-10(-5) M) reduced the spontaneous heart rate and prolonged the sinus node recovery time of guinea pig right atria. No effects on rat PM or atria, even after 10(-5) M, indicate a selective action of UK on the delayed rectifying outward potassium current, Ik. These results indicate a potent and selective, rate-dependent class III antiarrhythmic action of UK-68,798 linked with positive inotropy. Increased APD, bradycardia, and induction of EADs, however, represent a potential arrhythmogenic combination^38^.

Limitations of Action potential and refractory period in isolated guinea pig papillary muscle used to assess ventricular arrhythmias but not Atrial Fibrillation-Flutter & Torsades de Pointes Arrhythmias.

#### Langendorff technique

This technique's fundamental idea is that the heart is supplied with oxygenated saline solutions at constant pressure or flow in a retrograde route from the aorta. Similar to the in-situ heart during diastole, retrograde perfusion shuts the aortic valves. Through the coronary arteries branching off the coronary sinus and the expanded right atrium, the perfusate is displaced. By stunning, guinea pigs of either sex that weigh between 300 and 500 g are sacrificed. At 37, the heart was rapidly removed and placed in a dish with Ringer's solution. Lung and pericardial tissues that are related are taken out. Below the point of division, the aorta is located and severed. The aorta is punctured, the cannula is tied, and oxygenated Ringer's solution is then injected into the heart. The heart is moved to a double-walled perfusion device made of plexiglass that is kept at a temperature of 37 °C. At a constant pressure and temperature of 40 mm Hg and 37 °C, oxygenated Ringer's solution is perfused from a reservoir. The LAD coronary artery is bound with a ligament, and an occlusion is maintained for 10 min before reperfusion. Before or after occlusion, test chemical is given via perfusion medium. Because pulsatile stimulation and arrhythmia induction involve epicardial ECG electrodes (rectangular pulses of 0.75 ms duration, usually of 10 V; frequency 400–1800 shocks per min). At the top of the heart is a little steel hook with a rope. A force transducer measures contractile force isometrically, and the results are recorded on a polygraph. A chronometer connected to the polygraph measures heart rate. The perfusion medium is infused with drugs. Both the control group and the test group's ventricular fibrillation or ventricular tachycardia frequency and duration are noted^39^.

**Figure 5:**
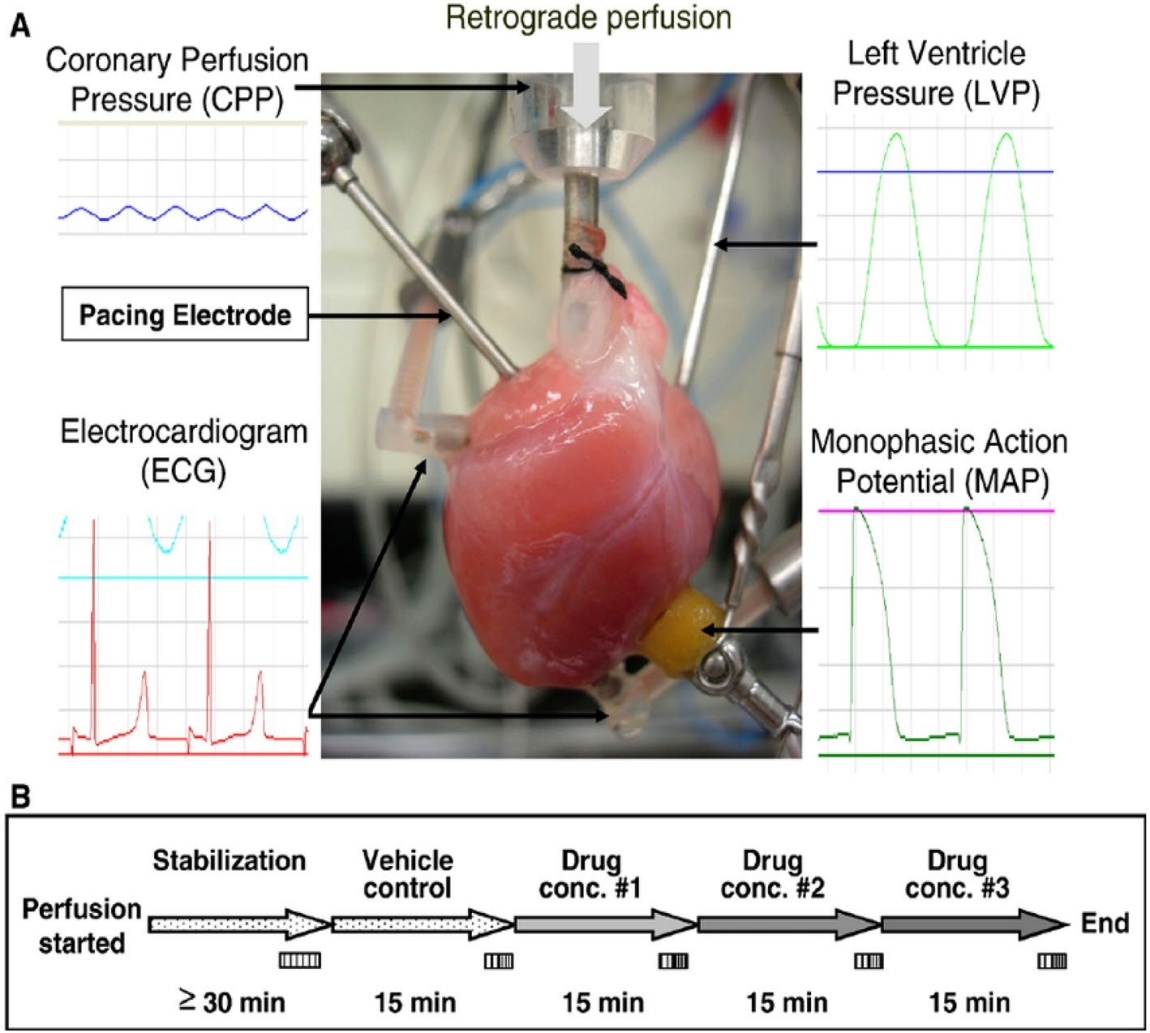
Shows the experimental methodology and the separated core. (**A**). Isolated guinea pig heart installed on the Langendorff apparatus showing the front view with all the electrodes and probes in place. (**B**). The usual procedure required at least 30 min for cardiac perfusion stabilization, followed by four periods of 15 min each for exposure to baseline and vehicle in three escalating doses.CPP, LVP, ECG and MAP were continuously monitored and recorded throughout the study. The horizontal bars show that the heart is beating freely and automatically, except slowly. (Guo L, Dong Z, Guthrie H. Validation of a guinea pig Langendorff heart model for assessing potential cardiovascular liability of drug candidates. Journal of Pharmacological and Toxicological Methods. 2009 Sep-Oct, 60(2):130–151. PMID: 19616638. 10.1016/j.vasch.2009.07.002))^243^.

**Figure 6:**
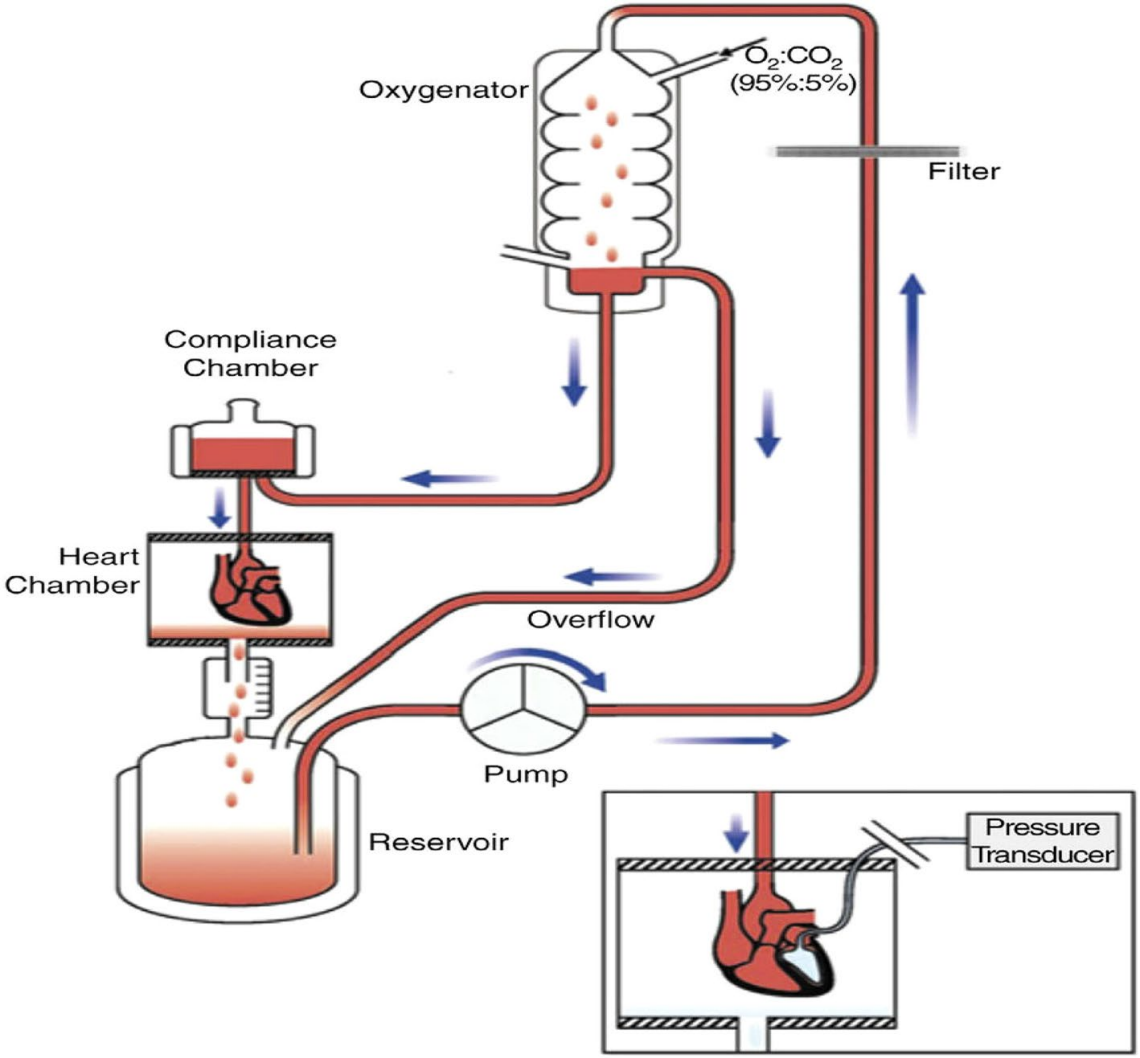
Isolated Langendorff heart perfusion model, (Ravelli F, Allessie MA. Effects of atrial dilatation on refractory period and vulnerability to atrial fibrillation in the isolated Langendorff-perfused rabbit heart. Circulation 1997 Sep 2;96(5):1686–95. PMID: 9315565. 10.1161/01.cir.96.5.1686))^39^.

Increased atrial pressure in the isolated rabbit heart resulted in a significant increase in vulnerability to Atrial fibrillation (AF) that was closely correlated to shortening of the Right and left atrial effective refractory periods (AERPs). These changes were completely reversible within 3 min after release of the atrial stretch, resulting in prompt termination of AF (Figs. 5 and 6)^39^. There are no limitations Langendorff technique have vulnerability to atrial and ventricular Fibrillation-Flutter.

#### Acetylcholine or potassium-induced arrhythmia

New Zealand white rabbits weighing between 0.5 and 3 kg were used for the experiment. The hearts of the slaughtered animals are immediately removed. In Ringer's solution, more tissue is removed from the atria. There is an electrode at the bottom of the tank to which the vestibules are connected and used. The atria are fibrillated with acetylcholine (3 × 10 g/mL) or potassium chloride (0.10 g). (Frequency: 400–1800 impacts per minute). Machines are used to create kymographic records. It takes up to 10 min to treat arrhythmias to prevent them from developing and continuing. After a 30 min inactivity period, the test chemical is added to the bath and fibrillation continues. After administration of the test material, the preparation is washed off and if the atria do not stop fibrillating within 8–10 min, normal contraction resumes.The effectiveness of the test chemical is determined by whether the fibrillation disappears immediately or within five minutes of placing the test drug in the organ bath^40,41^.

There are no limitations, Acetylcholine or potassium-induced arrhythmia, vulnerability to atrial and ventricular Fibrillation.

## Animal models of acquired arrhythmia disorders

Acquired heart disease is the most common cause of increased arrhythmia risk in humans. Acquired heart disease develops over the course of a person’s life becasue of structural remodeling associated with hypertension^42^, coronary artery disease^43^, nonischemic cardiomyopathy^44^, primary and secondary valvular disease^45^, autoimmune rheumatic diseases^44^, and myocarditis^46^. Acute cardiac stress or injury leads to activation of specific cell signaling pathways^47,48^, mitochondrial dysfunction^49,50^, altered Ca2 + handling^51–53^, and a switch in metabolism^54^ from fatty acid oxidation to glycolysis^55^. This leads to chronic changes in gene expression of trophic and mitotic factors, inflammation, and ultimately to myocyte hypertrophy and fibrosis. Pathological fibrosis is characterized by excessive proliferation of cardiac fibroblasts and ECM (extracellular matrix) protein deposition. Several key profibrotic factors have been identified, including TGF (transforming growth factor)-β, angiotensin II and aldosterone, which contribute to the development of cardiac fibrosis rssegardless of the underlying pathology^56^. However, the relative contribution of a distinct molecular pathway depends on the type and the degree of the initial cardiac injury. Various animal models have been developed to study aspects of these acute and chronic changes leading to arrhythmia risk and are discussed below (Table 1).

**Table 1:** Animal models of arrhythmia disorders.

Animal	Notes/limitations	
In vitro models		
Pig	Studies on isolated ventricular Myocytes: Can be induced minimally invasively with low mortality, model of TdP	
Pig, Dog	Isolated guinea pig papillary muscles: Limitation to ventricular arrhythmia	
Pig	Patch-Clamp Experiments in CHO Cells: Limitation to atrial Fibrillation	
Pig	Isolation of Porcine Atrial Myocytes: Limitation to atrial Fibrillation	
Pig	Isolation of guinea pig ventricular myocytes: Limitation to ventricular arrhythmia	
Pig, Rat	Isolated guinea pig papillary muscle: action potential and refractory period: Limitation to ventricular arrhythmia	
Rat, Pig, Dog, Rabbit	Langendorff technique: vulnerability to atrial and ventricular Fibrillation	
Rabbit	Acetylcholine or potassium-induced arrhythmia: vulnerability to atrial and ventricular Fibrillation	
Animal models of aquired arrhythmia disorders		
Transverse aortic constriction		
Mouse	Severity of injury dependent on strain (BALB/c > C57BL/6 > 129S1/SvImJ) and sub-strain (C57BL/6Tac > C57BL/6NCrl > C57BL/6J). Model requires PES to induce in vivo arrhythmias	
Rat	Develop spontaneous arrhythmias with catecholamine challenge	
Guinea Pig	High mortality, develops catecholamine-induced arrhythmias	
Rabbit	In vivo injury, but arrhythmia studies completed ex vivo with Langendorff system	
Pig	Model of heart failure with preserved ejection fraction, study of arrhythmias	
Sheep	Model of heart failure with reduced ejection fraction, study of arrhythmias	
Myocardial ischemia		
Mouse	Single left coronary artery leads to variation in severity of injury. Model requires PES with catecholamine challenge to induced arrhythmias	
Rat	Requires PES and catecholamine challenge to induce ventricular arrhythmias reliably but rare spontaneous arrhythmias	
Rabbit	PES done as ex vivo in Langendorff system	
Dog	Atrial ischemia extensive studied	
Pig	High mortality due to poor collateral circulation, early spontaneous VT/VF during injury and late model of SCD	
Sheep	High mortality with spontaneous ventricular arrhythmia	
AV node ablation		
Rat	High mortality, particularly in male rats. Requires specialized surgical equipment and skill	
Rabbit	Studied completed ex vivo in Langendorff system	
Dog	Can be induced minimally invasively with low mortality	
Sheep	Can be induced minimally invasively with low mortality, model of TdP	
Chronic atrial pacing		
Rat		Model for AF
Rabbit		In vivo pacing, but PES studied completed ex vivo with Langendorff system
Dog		AF and spontaneous VT model, recapitulates tachycardia mediated cardiomyopathy
Pig		AF model, recapitulates tachycardia-mediated cardiomyopathy
Chronic ventricular pacing		
Mouse		Requires tethered or ex vivo pacing
Rat		Model tachycardia mediated cardiomyopathy with VF induction with rapid pacing
Dog		Recapitulates tachycardia-mediated cardiomyopathy, model of spontaneous AF and VT
Sheep		Recapitulates tachycardia mediated cardiomyopathy with reduced ejection fraction, no studies of arrhythmia
Pig		
Inflammation		
Mouse		Strain-specific susceptibility. C3H/He and DBA/2 mice susceptible to viral myocarditits while C57BL/6 are protected. BALB/c susceptible to immunogen induced myocarditis while C57BL/6 more resistive
Rat		Model of AF, but nonphysiological induction of inflammation with talc
Guinea Pig		
Dog		
Sheep		
Metabolic/drug-induced		
Mouse		Streptozotocin and DIO models well established, increased susceptibility to AF and VT with PES
Rat		Age-dependent fibrosis found in Fisher 344 rat strain, model of AF
Rabbit		Established model of clofilium-induced TdP
In vivo approaches		
Arrhythmia of chemical origin		
Male Ivanovas rats		Rats with Aconitine Antagonism: With regard to ventricular extrasystoles, tachycardia, fibrillation, and death, the antiarrhythmic action of the test substance is quantified
Guinea Pig		Arrhythmias in Whole Guinea Pigs Caused by Aconitine: induce ventricular arrhythmias in a whole animal model
Male Marioth guinea pigs		Digioxin developed arrhythmia in guinea pigs: ventricular premature beats, fibrillation, and cardiac arrest
Rats, Rabbits		Strophanthin/Ouabain-induced Arrhythmia: ventricular premature beats, fibrillation, and cardiac arrest
Dogs		Adrenaline-induced Arrhythmia: induce ventricular arrhythmias in a whole animal model
Rats		Calcium-induced arrhythmia: ventricular flutter and fibrillation
Electrically triggered Arrhythmia		
Dogs		Ventricular Fibrillation Electrical Threshold: Atria & ventricular thresholds were assessed using a variety of electrical pacing techniques, including sequential pulse pacing
Dogs		Programmed Electrical stimulation induced Arrhythmia: sustained ventricular tachycardia and ventricular fibrillation
Male mongrel dogs		Dog Model of Sudden Coronary Death: Coronary artery stenosis investigations into the onset of lethal arrhythmia and ventricular ectopy are conducted using recordings from the cardiocassette evaluation of tachyarrhythmias
Rats, Dogs		Exercise-related ventricular fibrillation: Coronary artery stenosis of exercise on the treadmill
Mechanically generated arrhythmia		
Rats		Reperfusion Arrhythmia in Rats: Ventricular arrhythmia and myocardial infarction by ligation of the left major coronary artery in the course of the ligation and subsequent reperfusion
Dogs		Reperfusion Arrhythmia in Dogs: Ventricular arrhythmia and myocardial infarction by ligation of the left major coronary artery in the course of the ligation and subsequent reperfusion
Mice, Rats, Dogs, Guinea Pigs, Rabbits, Monkeys		Genetically Prone Arrhythmias. Limitations: Limited to embrogenic gene manupulations, ie. Gene mutations and gene knockout varients
Mice, Rats, Dogs, Guinea Pigs, Rabbits, Monkeys & Humans Stem cell cardiomyocytes		Arrhythmia Mechanisms in Human Induced Pluripotent Stem Cell-Derived Cardiomyocytes; Limitations: When compared with native human ventricular tissue, hiPSC-based EHTs lack a positive force–frequency relation, which is one of the hallmarks of cardiac contractility. Furthermore, the frequency-dependent acceleration of relaxation is much weaker in EHTs

Representative list of animal models with reference to the method to their development. AF indicates atrial fibrillation; DIO, diet-induced obesity; PES, programmed electrical stimulation; SCD, sudden cardiac death; TdP, Torsades de Pointes; VF, ventricular fibrillation; and VT, ventricular tachycardia.

### Transverse aortic constriction

The transverse aortic constriction (TAC) model mimics chronic hypertension or aortic stenosis by causing a stricture in the thoracic aorta^57^ Left ventricular and atrial pressure overload increases the wall tension leading to hypertrophy, chamber dilation, and fibrosis^58,59^. This can be done in various methods, including sutures^57,58^, inflatable cuffs^60^ or intravascular stents^59,61,62^. Within hours of injury, myocyte hypertrophy is induced by activation of p38 MAP Kinase^63^, ERK1/2^64^, and PI3K/AKT signaling^65^. Increased TGF-β production due to cytokine^66^ and adrenergic receptor activation^67,68^ leads to fibroblast proliferation and collagen deposition. The reninangiotensin-aldosterone system plays an important role in developing hypertrophy and arrhythmia, as treatment with ACE inhibitors^69^ and spironolactone^70^ reduces fibrosis and improving conduction velocity. This initially leads to ventricular hypertrophy, later followed by chamber dilation^66^. While recruitment of Ly6ClowCXCR1 + macrophages has been found in the LV early after TAC^71^, there is histologically less inflammation in this model than in other cardiac injury models^64^. Notable in this model is the upregulation of NCX^72^ and downregulation of SERCA2a in myocytes over time, which is also seen in explant human hearts with reduced ejection fraction. While restoring SERCA2 has shown promise in improving systolic dysfunction^73,74^ and suppression of ventricular arrhythmias^75,76^ in animal models, the CUPID2 trial in patients with human hearts with reduced ejection fraction showed neutral results^77^. In mice with TAC, multiple investigators have shown an increase conduction time, AP duration, and AV nodal refractory period, QT prolongation with inducible atrial (50%–60%) and ventricular arrhythmias (40%–50%), but spontaneous arrhythmias in vivo are rare^78–81^. Redistribution of connexin-43 (Cx43) laterally away from intercalating disk has been purposed to explain, in part, the changes in conduction in this model^80^. Arrhythmia induction can be challenging in this model, which is likely due to variability in the extent of constriction postprocedure^82^, the length of time of injury before analysis, sex, and strain^83–87^. Care must be taken when comparing results from different injury protocols and strains in this model. Similarly, rabbits, rats, and guinea swine develop ventricular hypertrophy and dilation after TAC, albeit over a more extended period (8 versus 4 weeks in mice)^88^ and with a higher incidence of sudden cardiac death. Unlike the mouse model, rabbits^89–91^ (aortic insufficiency with abdominal aortic constriction), rats^75,79^, and guinea pigs^92–94^, develop spontaneous arrhythmias in response to catecholamine challenge. Similarly, these animals develop ventricular hypertrophy, chamber dilation and fibrosis after TAC, albeit over a more extended period (8 versus 4 weeks in mice)^88^, and with a higher incidence of sudden cardiac death. AP prolongation is a hallmark in these models and is linked to increased INaL and INCX with reduced ICaL responsiveness to β-adrenergic stimulus and increased CaMKII activity, which is seen in human heart failure myocytes^95^. Overall, these animals seem to represent a better model for arrhythmias than mice, albeit most studies use explanted heart in the Langendorff system. Unlike small animals, no studies report an increase in arrhythmia in large animals with TAC. Ascending aortic constriction in pig and sheep has been described with polyester band^96^, or an implanted inflatable cuff^60,97^. The swine model of TAC differs from other species as they tend to develop heart failure with preserved ejection fraction characterized by LV hypertrophy with diastolic dysfunction^98–101^. In contrast to pig, sheep develop cardiac dysfunction at 6 to 18 weeks post-TAC with elevated markers of ECM remodeling, chemokine production, and apoptosis^102^, but no study reported arrhythmia generation outside procedural effects.

Limitations: Severity of injury dependent on strain (BALB/c > C57BL/6 > 129S1/SvImJ) and sub-strain (C57BL/6Tac > C57BL/6NCrl > C57BL/6 J). Model requires PES to induce in vivo arrhythmias.

### Myocardial ischemia

Acute and chronic myocardial ischemia is a major cause of ventricular arrhythmias in humans. During acute ischemic, myocytes are exposed to hypoxia, acidosis, increased extracellular K + , and intracellular Ca2 + ^103,104^. Under ischemic conditions, cardiomyocyte mitochondria switch to glycolysis from fatty acid oxidation to maximize ATP production with limited oxygen supply^105^. In addition, hypoxia leads to reactive oxygen species production that further damages intracellular proteins and organelles^106^. Myocardial infarction occurs with myocyte apoptosis and replacement fibrosis if normal blood flow is not restored. This is a highly inflammatory model, with significant infiltration of CD11b + macrophages and upregulation of inflammatory cytokines (CCL2, TNF-α, and IL-10) after injury, leading to proinflammatory Ly6chighCCR2 + macrophages early, then pro-wound healing Ly6clowCXCR1 + chronically, which contribute to interstitial fibrosis in the border zone^107^. Chronically, myocardial fibrosis leads to conduction heterogeneity, a substrate for reentry arrhythmias. In mice and rats, myocardial ischemia is induced surgically, either transiently by ischemia/reperfusion (I/R) injury^108,109^ or complete occlusion by coronary artery ligation^108,110^ or cryoinjury^109,111,112^. Unlike complete occlusion, I/R injury produces reversible ischemia that leads to significant myocardial dysfunction without widespread necrosis in the area at risk. Spontaneous ventricular arrhythmias are observed during the reperfusion phase^108^. However, spontaneous ventricular arrhythmias are rare after complete occlusion aside from isolated preventricular contractions (internal data). In addition, LV dysfunction leads to volume overload in the left atrium, causing fibrosis and susceptibility to AF^113^. Atrial and ventricular arrhythmias can be induced universally in isolated explanted post-MI hearts, with the occurrence of VT (inducible in up to 90%–100% in mice), VF (inducible in up to 89% of rats), and AF (inducible in 73% of rats and 33% of mice)^114–120^. In vivo ventricular arrhythmia induction is more difficult, requiring rapid pacing protocols^121–123^ and a catecholamine challenge. Transvenous, pericardial, and transesophageal protocols have been described for AF and ventricular arrhythmia induction, with the occurrence of VT (20%–70% in mice and rats)^124^ and AF (60%–90% in mice)^125^. Electrophysiological study of isolated myocytes from acute and chronic infarcted hearts has shown both shortening of the myocardial effective refractory period and slowing of condition time in the left atria, infarct, and border zone. The proposed mechanism for AF induction is reduced expression of Cx40, dephosphorylation of Cx40 and Cx43, and redistribution from the intercalated disc to the lateral cell membrane. Established models for rabbit myocardial ischemia focus extensively on ex vivo studies using Langendorf perfusion systems. After infarction, rabbit myocardium shows prolonged APD and Ca2 + transients ex vivo^126^. Unlike mice and rats, infarction in rabbits leads to delayed AV nodal conduction becaue of fibrosis and reduction in Cx40 expression but did not affect the ventricular effective refractory period^127^. No arrhythmia induction protocol was used in these studies. Dog models focus on atrial ischemia and arrhythmias, with ~ 40% of animals developing AF^128^. Increased INCX current, spontaneous Ca2 + leak, and conduction heterogeneity were found in myocytes from the border zone of the atrial infarct, supporting both triggered and reentry as the mechanism in this model^129^. The use of beta-blocker (nadolol) and Ca2 + channel inhibitor (nifedipine) were more effective at suppressing atrial arrhythmia in this model than class Ic (flecainide) or class III (dofetilide) antiarrhythmic drugs^130^. Pig myocardial ischemia models are well established as they are of similar size and physiology as humans. Ischemia can be induced by either transient intravascular occlusion of the coronary artery or chronic occlusion with an ameroid constructor^131^. Acutely, pigs are exquisitely sensitive to ischemia because of lack of functional collaterals at baseline, which leads to significant procedural mortality from VF^132^. After chronic ischemia, SCD due to spontaneous ventricular arrhythmias occurs in ≈60% to 70% of animals by 3 months^133,134^. Myocytes in the remote zone from have a decreased rapid delayed rectifier K + current (IKr), altered Na + -Ca2 + exchange current (INCX), and increases of late Na + current (INaL), Ca2 + -activated K + current [IK(Ca)], and Ca2 +—activated Cl − current [ICl(Ca)]. In addition, myocytes in the border zone show the same changes along with a decrease of L-type Ca2 + current (ICaL), a decrease of inward rectifier K + current (IK1), and arrhythmogenic SR Ca2 + release-induced EADs and DADs^135^. These changes in the current lead to shortening of the APD in the border zone and prolonged APD in the remote region, setting up a substrate for both triggered and reentrant arrhythmias. Gene therapy with dominant-negative K + channel (KCNH2-G628S)^136^ or Cx43^137^ reduced VT induction by prolonging the ADP and effective refractory period in the border zone.

Limitations:

Mouse: Single left coronary artery leads to variation in severity of injury. Model require PES with catecholamine challenge to induced arrhythmias.

Rat: Requires PES and catecolamine challenge to induce ventricular arrhythmias reliably but rare spontaneous arrhythmias.

Rabbit: PES done as ex vivo in Langendroff system.

Dog: Atrial ischemia extensive studied.

Pig: High mortality due to poor collateral circulation, early spontaneous VT/VF during injury and late model of SCD.

Sheep: Model of heart failure with reduced ejection fraction, study of arrhythmias.

### Complete heart block and AV node ablation

Complete heart block leads to AV dyssynchrony and bradycardia, which acutely causes reduced cardiac output and induces volume overload. Compensatory hemodynamic changes occur to improve cardiac function, including ventricular dilation, hypertrophy, and increased stroke volume but are not able to fully restore cardiac output^138^. While genetic models are available for primary complete heart block, secondary complete heart block, which is characterized by fibrosis and necrosis of the AV node, is more difficult to reproduce. Outside histological remodeling, little is known about the molecular changes associated with secondary complete heart block. Given the size, mouse AV node is challenging to identify without immunostaining^139^. Genetic have generated to establish the important transcription factors and ion channels in the AV node (noted in the above section atrioventricular block). Interestingly, disruption of tissue resident macrophages in the AV node, by either knockout of Cx43 in these cells or by genetic ablation of macrophages using diphtheria toxin receptor/diphtheria treatment, lead to progressive AV conduction block^140^. While these resident macrophages were noted in human AV nodes, it unclear if the presence or absence in of these cells contribute to human disease. Electrical needle AV nodal ablation in rats has been described, as it was noted that alcohol injection leads to either transient block or mortality based on the amount used^141,142^. This is a technically challenging model with variable success^142^ and high early mortality because of bradycardia and ventricular arrhythmias, as only 6-month-old female rats survived past 3 days^143^. Regardless, the surviving rats recapitulated the cardiac remodeling in humans, with 80% showing spontaneous TdP at baseline which could be induced to sustained VT with PES and isoproterenol challenge^144^. The dog model of AV ablation by transvenous catheter injection for formaldehyde has been well established and leads to compensated hypertrophy and QT prolongation^140,143^. This is due to APD prolongation in the setting of bradycardia, with ≈50% of animals developing spontaneous and 90% drug-induced TdP^144,145^. Chronically, this reduced IKs current and enhanced Ca2 + influx by NCX, leading to increased SR Ca2 + content^142^, which increases the risk for DADs^146^. In contrast, goats undergoing AV ablation did not show APD prolongation but led to increased PLB (phospholamban), Troponin-I and myosin light chain kinase by PKA and RyR2 by CaMKII, suggesting increased Ca2 + sensitivity in this model^147^.

Limitations:

Rat: High mortality, particularly in male rats. Requires specialized surgical equipment and skill.

Rabbit: Studied completed ex vivo in Langendorff system.

Dog: Can be induced minimally invasively with low mortality.

Sheep: Can be induced minimally invasively with low mortality, model of TdP.

### Chronic tachypacing

Overriding the normal conduction system has many deleterious effects but is often reversible. Rapid atrial heart rate or pacing can lead to tachycardia-mediated heart failure, and long-term RV pacing in humans can lead to ventricular dyssynchrony, reduced cardiac output, and heart failure^146^. Long-term pacing has been shown to be detrimental to LV, with significant wall motion abnormalities and perfusion defects in the inferior and apical walls without corresponding coronary disease^148^. Conversely, cardiac resynchronization therapy improves HF outcomes for patients with HF and left-bundle branch block. RV pacing has been used as a model of nonischemic cardiomyopathy, developing significant systolic dysfunction. Long-term RV pacing in AV nodal ablated dogs show the same perfusion mismatches seen in humans and was associated with increased sympathetic innervation of the ventricles^149^. Overdrive RV pacing for 4 weeks in dogs can induce spontaneous ventricular arrhythmias and SCD in 25% of dogs^150^. Myocytes isolated from paced ventricles showed prolonged ADP with reduced Ito currents^151^, likely due in part to downregulation of Kv4.3^152^ and increased INa,L^153^ in failing heart. In addition, Ca2 + transients showed reduced amplitude, slowed relaxation, and blunted frequency dependence due to reduction in SERCA2a and upregulation of NCX in failing myocytes^154^. Cardiac resynchronization therapy in this model was showed to normalization of APD, reduce the INa,L current, and prevent the negative remodeling associated with heart failure in this model^153,155,156^. VF can be induced in pig by applying AC current to the RV, but using arrhythmic drugs to improve resuscitation was not seen^157^. Ventricular pacing in dogs also leads to secondary atrial fibrosis, dilation, and reduced function as the LV fails, inducing AF^158,159^. As with rapid ventricular pacing, rapid atrial pacing leads to a reduction in the Ito, in addition to ICa,L and IKs currents^160^. If pacing is stopped and the animal is allowed to recover, the ion currents return to normal, but fibrosis remains, leading to persistent AF. Studying atrial cells from dogs undergoing both rapid atrial and ventricular pacing has shown the atrial ion channel expression in HF, AT, and HF with AT can be significantly different^161^. Upregulation of profibrotic miRNA has also been described in atrial paced dogs, providing a novel target to prevent atrial fibrosis and AF^162,163^. In rabbits, rapid atrial pacing increases atrial fibrosis and TGF-β signaling, which can be attenuated with losartan^162^. AF can be induced in 40% of rabbits in this model. Atrial pacing leads to decrease KCNE1 KCNB2 expression, reduced IKs, and shortening the AERP^164^. This was thought to be due to microRNA-1 upregulation in the atria. In sheep, natriuretic peptide release is found immediately after RV pacing, returning to normal after cessation^165^. In goats, chronic atrial pacing led to atrial dilation, with reduced PKA phosphorylation of PLB and increased CaMKII phosphorylation of RyR2, leading to reduced SR Ca2 + load^147^. While structural changes similar to humans with tachycardia mediated cardiomyopathy are seen, increased arrhythmogenesis has not been reported. Given their small size, in vivo pacing is difficult in mice. Tethered epicardial pacing has been used to study AV dyssynchrony and synchrony in mice after I/R injury. Dyssynchrony leads to further deterioration of cardiac function and activation of p38, ERK1/2, JNK, and MSK1 and inhibition of the GSK3β pathways. This was reversed by resynchrony^166^. Recently, the development of fully implantable epicardial micro pacing technology may allow for longitudinal pacing studies^167^. In rats, initiation of rapid atrial pacing leads to upregulation of multiple voltage-gated K + channels (Kv1.5, Kv4.2, and Kv4.3)^168^, which contribute to repolarization by I Kr and ITo. After 2 days of atrial pacing, AF can be induced in ≈20% of animals, with upregulation of associated AF genes (CASQ2, KCNJ2, and TGFB) and activation of the TGF-β and IL-6 pathways^169^. Rapid transesophageal pacing of the LV has been used to reliably induce VF to study the effects of medication for resuscitation^170^.

Limitations:

Chronic atrial pacing.

Rat: Model of Atrial Fibrillation-Flutter.

Rabbit: In vivo pacing, but PES studied completed ex vivo with Langendorff system.

Dog: AF and spontaneous VT model, recapitulates tachycardia mediated cardiomyopathy.

Pig: AF model, recapitulates tachycardia-mediated cardiomyopathy.

Chronic ventricular pacing.

Mouse: Requires tethered or ex vivo pacing.

Rat: Model tachycardia mediated cardiomyopathy with VF induction with rapid pacing.

Dog: Recapitulates tachycardia-mediated cardiomyopathy, model of spontaneous AF and VT.

Sheep/Pig: Recapitulates tachycardia mediated cardiomyopathy with reduced ejection fraction, no studies of arrhythmia.

### Inflammation

Myocardial inflammation is a known driver of atrial and ventricular arrhythmias^171,172^. Postprocedural arrhythmias are common after cardiothoracic surgery but, while they are usually self-limiting, they lead to prolonged hospital stays^173^. While there is usually a preexisting arrhythmogenic substrate due to the underlying disease, surgical scarring and inflammation further exacerbate the system, leading to arrhythmia. Large cohort studies have shown elevated proinflammatory cytokines associated with persistent AF, including CRP^174–176^, TNF-α, IL-1β, IL-6, and IL-10^177,178^. After surgery, there is an increase in both macrophages and neutrophils to the surgical site, with reactive oxygen species production from MPO (myeloperoxidase) activity^179,180^. Inflammation because of myocarditis is more complex, and the course of the acute and chronic phase of the immune response is dependent on the underlying cause (infectious versus rheumatological). The cytokine profiles are found in viral and autoimmune myocarditis includes more proinflammatory monocyte infiltration and myocyte necrosis than postsurgical injury^181^. This leads to a multitude of ECG changes, including sinus tachycardia, widened QRS patterns, low voltage, prolonged QT, variable AV blocks, and diffuse ST-elevations^182–184^. Sterile inflammation has been used to induce AF in dogs^183^ and sheep^185^. Pericardial talc treatment of dog atria to induce sterile inflammation induced AF in ≈60% of dogs and was significantly reduced with topical steroids or NSAIDS^185^. In sheep, treatment with atorvastatin reduced hs-CRP, IL-6, and TNF-α expression, which improved the atrial effective refractory period at 72 hours^186^. While inflammatory myocarditis can be induced in rats^187^ and guinea pigs^188^, no current reports on the arrhythmia potential are available. Currently, there are 2 models of viral myocarditis due to exposure to coxsackievirus B3 (CVB3)^189^ and encephalomyocarditis virus A (EMCV)^190^. There are several issues with the viral myocarditis models in animals. First, of the viruses primarily associated with human myocarditis (parvovirus B19, herpes simplex 9 and coxsackievirus B3), only CVB3 is infectious to animals, with EMCV only rarely causing human disease newborns. Second, the CVB3 mouse model is highly inflammatory and more mimics childhood infections than the milder course in adults^191^. Third, only particular stains of mice are susceptible to viral infection. Regardless, C3H/He mice exposed to develop similar arrhythmias to humans (80% sinus arrest, 30% second or third-degree AV block, 30% PACs, 20% PVCs, and 10% VT)^192^. Ex vivo electrophysiological studies showed no change to the APD, but mice exposed to CVB3 were hyperpolarized with slightly increased VERP^193^. EMCV exposed DBA/2 mice develop AV block in 40% of mice over 2 weeks, with two-thirds of those mice showing mononuclear cell infiltration and edema and another onethird showing necrosis of the conduction system^194^. No further electrophysiological studies were conducted. Traditionally, myocarditis can be induced in mice by either immunizing with a cardiac structural peptide (myosin heavy chain [MHC-α] or cardiac troponin I)^195^ or delivering primed dendritic cells pulsed with MHCα^196^. Importantly, BALB/c mice are susceptible to peptide immunized myocarditis while C57BL/6 strains are resistant, from which a majority of transgenic lines are created^197^. MHC-α peptide-induced myocarditis showed significant immune cell infiltration, increased expression of both TNFα and INFγ, fibrosis and prolongation for the APD in ventricular myocytes, which all could be attenuated with atorvastatin^187,188,198^. Ctla4 + / − Pdcd1 − / − mice spontaneously develop myocarditis, modeling immune checkpoint inhibitor–induced myocarditis^199^. These mice succumb to progressive ventricular hypertrophy and SCD early. Progressive AV block and sinus arrest occurs in ~ 30% of transgenic mice, similar to arrhythmia seen in patient with immune checkpoint inhibitor–induced myocarditis. Further study using this model would greatly improve treatment for patient with adverse events after immune checkpoint inhibitor therapy.

Limitations:

Mouse: Strain-specific susceptibility. C3H/He and DBA/2 mice susceptible to viral myocarditits while C57BL/6 are protected. BALB/c susceptible to immunogen induced myocarditis while C57BL/6 more resistive.

Rat/Guinea Pig/Dog/Sheep: Model of AF, but nonphysiological induction of inflammation with talc.

### Metabolic- and drug-induced arrhythmia

Dietary and metabolic considerations also contribute to atrial arrhythmia development as there is a known association with BMI and diabetes in AF^200,201^, and incidence of paroxysmal AF is reduced after gastric bypass surgery^202^. Off-target drug effects lead to adverse clinical outcomes, particularly arrhythmias induction due to block of delayed rectifier K + channels, IKr, causing drug-induced long QT syndrome and TdP ventricular arrhythmias. Streptozotocin-induced diabetic models have been developed for both mice and rats^203^ and consistently shown prolonged APD, increased sympathetic innervations, and inflammation. Studies have shown this is likely due to production of advanced glycation end products (AGEs) in diabetes animals, but they are inconstant in the electrophysiological mechanisms^204^. Initial studies showed a reduction in the Ito current as the underlying cause of AF^205–207^, while others suggest changes in sinoatrial node connexin channel expression^208,209^, atrial myocyte Ca2 + handling proteins (TRPC1/6, RyR3)^210^ and AV nodal ion channels (TRPC1, CASQ2, RYR2, and RYR3)^211^ are involved. Diabetic mice and rats also have reduced K + channel expression, leading to overall reduced K + currents, which was dependent on glycosylation of CaMKII and activation PKC^212^. Further studies showed in the setting of hyperglycemia, mice had more diastolic calcium leak through RyR2, prolonged ADP90, ADP alternans, increased DADs, and frequent premature ventricular complex, which could be suppressed by genetic inhibition of CaMKII^213^. Blocking IL-1β activation of CaMKII in this model restores the APD to normal and suppresses VT induction in explanted hearts from diabetic mice^214^. These studies all provide a central role for CaMKII in regulating ion channel expression and function in hyperglycemia. Diet-induced obesity has been shown to affect inflammation and gene expression in multiple disease models. Mice fed 3 months of a high-fat diet had a 15% increase in their QTc and increased IKs current than mice on a normal diet and developed a tenfold increase in premature ventricular complex burden^215^. The increased IKs current was thought to be due to the reduced expression of voltagegated K + channels. Diet-induced obesity mice were also found to have reduced NaV1.5 expression and current, leading to reduced ADP and conduction velocity in the atrial and increased incidence of induced AF^216^. Diet-induced obesity rats over 8 weeks have increased expression of CaV1.2, HCN4, Kir2.1, RYR2, NCX, and SERCA2a in the LV, which may contribute to DADs and triggered PVCs^217^. Rabbits fed a high-fat diet had increased cardiac sympathetic innervation (as seen by increased GAP23 expression), prolonged ADP and increased ICa, which led to QTc prolongation and repolarization heterogeneity in the ventricle^218^. Isolated hearts were more susceptible to VT induction. While these studies highlight the important changes to ion channel expression animals due to a high-fat Western diet, further study is needed to linking these findings to humans. A specific line of inbred rats (Fischer F344 at 20–24 months) develop age-dependent adverse cardiac remodeling, with males developing more cardiomyocyte hypertrophy, intestinal fibrosis, and systolic dysfunction and females with more cardiac hypertrophy and diastolic dysfunction^219^. Aged female Fischer F344 rats show enlarged atrial, fibrosis, and CD68 + monocyte infiltration, similar to human disease221.452 Both male and female Fischer 344 rats are more susceptible to AF induction by atrial pacing, with 80% of animals showing atrial arrhythmia^220,221^. These are the only models of spontaneous AF in aged animals and could provide important insight to mechanism and future therapies for AF in our aging population. In rabbits treated with clofilium (K + channel blocker), 70% of animals develop TdP, which can be increased to 100% with the addition of α1 agonist methoxamine^222^. Blockade of CaMK (calmodulin kinase) or PKA in this model reduced pause-dependent VT that was independent of these kinases effect on L-type Ca2 + channel activity^223^. Interestingly, the dose-dependent CaMKII blockade did not change the QT interval, unlike the dosedependent PKA blockade. Further study showed that blocking CaMKII reduced the ratio of the TU interval, which is likely more critical than the QT interval in the induction of pause-dependent VT^224^.

Limitations:

Mouse: Streptozotocin and DIO models well established, increased susceptibility to AF and VT with PES.

Rat: Age-dependent fibrosis found in Fisher 344 rat strain, model of AF.

Rabbit: Established model of clofilium-induced TdP.

## In vivo approaches

The following are examples of in vivo models used to evaluate potential new antiarrhythmic drugs:

### Arrhythmia of chemical origin

Arrhythmias can be caused by many drugs alone or in combination. Arrhythmias result from the concomitant use of sensitizing drugs such as intravenous adrenaline and anesthetics such as ether, chloroform, or halothane. These arrhythmic substances have different effects in different animal species.

**Limitations-comments:**

Male Ivanovas rats: Rats with Aconitine Antagonism: With regard to ventricular extrasystoles, tachycardia, fibrillation, and death, the antiarrhythmic action of the test substance is quantified.

Guinea Pig: Arrhythmias in Whole Guinea Pigs Caused by Aconitine: induce ventricular arrhythmias in a whole animal model.

Male Marioth guinea pigs: Digioxin developed arrhythmia in guinea pigs: ventricular premature beats, fibrillation, and cardiac arrest.

Rats, Rabbits: Strophanthin/Ouabain-induced Arrhythmia: ventricular premature beats, fibrillation, and cardiac arrest.

Dogs: Adrenaline-induced Arrhythmia: induce ventricular arrhythmias in a whole animal model.

Rats: Calcium-induced arrhythmia: ventricular flutter and fibrillation.

#### Rats with aconitine antagonism

Activating sodium channels over time, aconitine, a plant alkaloid from aconitine root, causes ventricular arrhythmias. Rats that have consumed aconitine can be used to test drugs that are thought to have anti-arrhythmic effects. Urethane (1.25 g/kg) is administered intraperitoneally to anaesthetize male Ivanovas rats (300–400 g). Aconitine (5 ug/kg) is dissolved in 0.1 N HNO_3_ and administered 0.1 ml/min continuously into the rat's saphenous vein. Every 30 s, an ECG in lead II is recorded. 5 min before to the infusion of aconitine, a test substance is administered intravenously or orally. The test group receives a larger dose of aconitine than the untreated group, which results in a measure of antiarrhythmic activity. With regard to ventricular extrasystoles, tachycardia, fibrillation, and death, the antiarrhythmic action of the test substance is quantified by the amount of aconitine/100 g animal (infusion duration)^225,226^.

#### Arrhythmias in whole guinea pigs caused by aconitine

**Figure 7:**
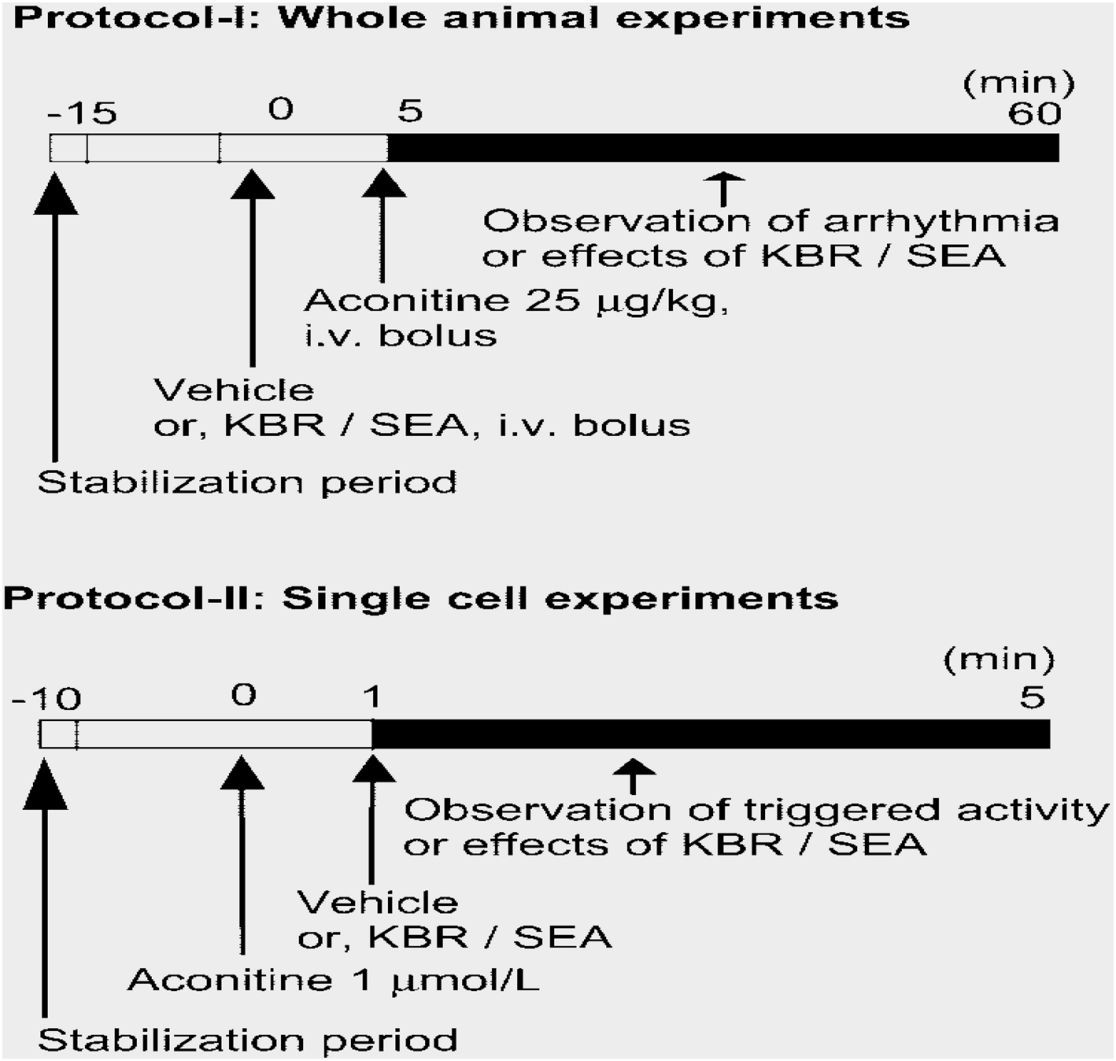
Overview of the test protocols. Protocol I was used for whole animal experiments and Protocol II for single cell experiments. In Protocol I, after stabilization, the medium or each dose of KBR or SEA was pretreated. Then aconitine (25 g/kg, IV)bolus has been delivered). The black column indicates the duration of the arrhythmia observation. A total of 130 animals were introduced into this series of experiments. 74 were used in Protocol I, but six were excluded based on exclusion criteria. The number of animals assigned to each control (vehicle), KBR and SEA is shown in Fig. 3. In Protocol II, after stabilization, aconitine (1 M) was infused first, then vehicle, or each dose of KBR or SEA was further elaborated. A total of 13 cells were included in Protocol II(Amran MS, Hashimoto K, Homma N. Effects of sodium-calcium exchange inhibitors, KB-R7943 and SEA0400, on aconitine-induced arrhythmias in guinea pigs in vivo, in vitro, and in computer simulation studies. Journal of Pharmacology and Experimental Therapeutics. 2004 Jul 1;310(1):83–9. PMID: 15028781. 10.1124/jpet.104.066951) )^227^.

Sodium pentobarbital (50 mg/kg i.p.) was used to anesthetize guinea pigs weighing between 300 and 400 g. In order to keep pCO2, pO2 and pH in the normal range, respiration was maintained by artificial respiration (ambient air, volume 1.5 ml/100 g, frequency 55 beats per minute).Aconitine and study drugs were administered through a polyethylene tube inserted into the right jugular vein. A cannula was inserted into the left carotid artery to measure systemic blood pressure. The ECG leads of the articulated limbs were recorded. A polygraph recorder (NEC San-ei Instruments Ltd., Tokyo, Japan) was used to continuously monitor the subjects' blood pressure, heart rate and ECG (Leads I and II). Vehicle or NCX inhibitors were administered to different groups for each drug or vehicle dose (ie, 0–30 mg/kg) by intravenous bolus injection in the jugular vein after 15 min of stabilization. To induce ventricular arrhythmias in a whole animal model (Lu and Clerck, 1993) (Fig. 1, Protocol I), 25 g/kg aconitine was administered five minutes later.Each animal received a single dose (treatment) of vehicle or one of the NCX inhibitors. The doses of SEA and KBR were 1 to 10 mg/kg and 1 to 30 mg/kg, respectively (Fig. 7)^227^.

#### Digioxin developed arrhythmia in guinea pigs

Overdose with digoxin leads to ventricular premature beats, fibrillation and death. Antiarrhythmic drugs prolong the duration of these symptoms. Male Marioth guinea pigs (350–500 g) are anesthetized by intraperitoneal administration of sodium pentobarbital (35 mg/kg). The animal is catheterized under mechanical ventilation (45 breaths per minute) in the trachea, jugular vein and carotid artery. Digoxin is administered into the jugular vein using an infusion pump at a dose of 85,g/kg.266 mL/min to cardiac arrest. Steel needle electrodes are used to record the ECG throughout the experiment. The carotid artery is used to measure blood pressure. The study drug will be administered intravenously or orally one hour before the infusion. Note the interval between ventricular premature beats, fibrillation, and cardiac arrest. The total amount of digoxin delivered (μg/kg) to induce VF, VF and cardiac arrest after treatment with study drug is statistically compared to a control group receiving digoxin alone^228^.

#### Strophanthin/ouabain-induced arrhythmia

The carotid artery is used to measure blood pressure. The study drug will be administered intravenously or orally one hour before the infusion. Note the interval between ventricular premature beats, fibrillation, and cardiac arrest. The total amount of digoxin delivered (μg/kg) to induce VF, VF and cardiac arrest after treatment with study drug is statistically compared to a control group receiving digoxin alone. The test material is administered 10 min after the arrhythmia has resolved. The test substance is considered antiarrhythmic if the extrasystoles cease immediately after ingestion of the drug. If the study drug does not show a positive effect, higher doses are given 15 min apart. If the test chemical is effective in reversing the arrhythmias, the next dose is administered when a sustained arrhythmia recurs^229,230^.

#### Adrenaline-induced arrhythmia

High doses of adrenaline may cause arrhythmia to occur. Pentobarbitone sodium (30–40 mg/kg) is administered intraperitoneally to dogs (10–11 kg) to induce anaesthesia. A cannula is placed in the femoral vein, and 2–2.5 g/kg of adrenaline is then infused. Atrial and Lead II ECGs are both recorded. Three minutes after the infusion of adrenaline, the test medication is given^231^. A test substance is deemed to have an antiarrhythmic action if extrasystoles stop occurring right away after taking the medication.

#### Calcium-induced arrhythmia

Nembutal (60 mg/kg) is administered intraperitoneally to anesthetize albino Wistar rats (60–130 g). 2 ml/kg of a 10% aqueous solution of calcium chloride is injected into the femoral vein to induce ventricular flutter and fibrillation. Using a cardioscope connected to the animal via two percutaneous precordial clamp electrodes, the rhythm and behavior of the heart are observed during and two minutes after the injection.A test drug is administered two minutes before the calcium chloride infusion^232^.

**Figure 8:**
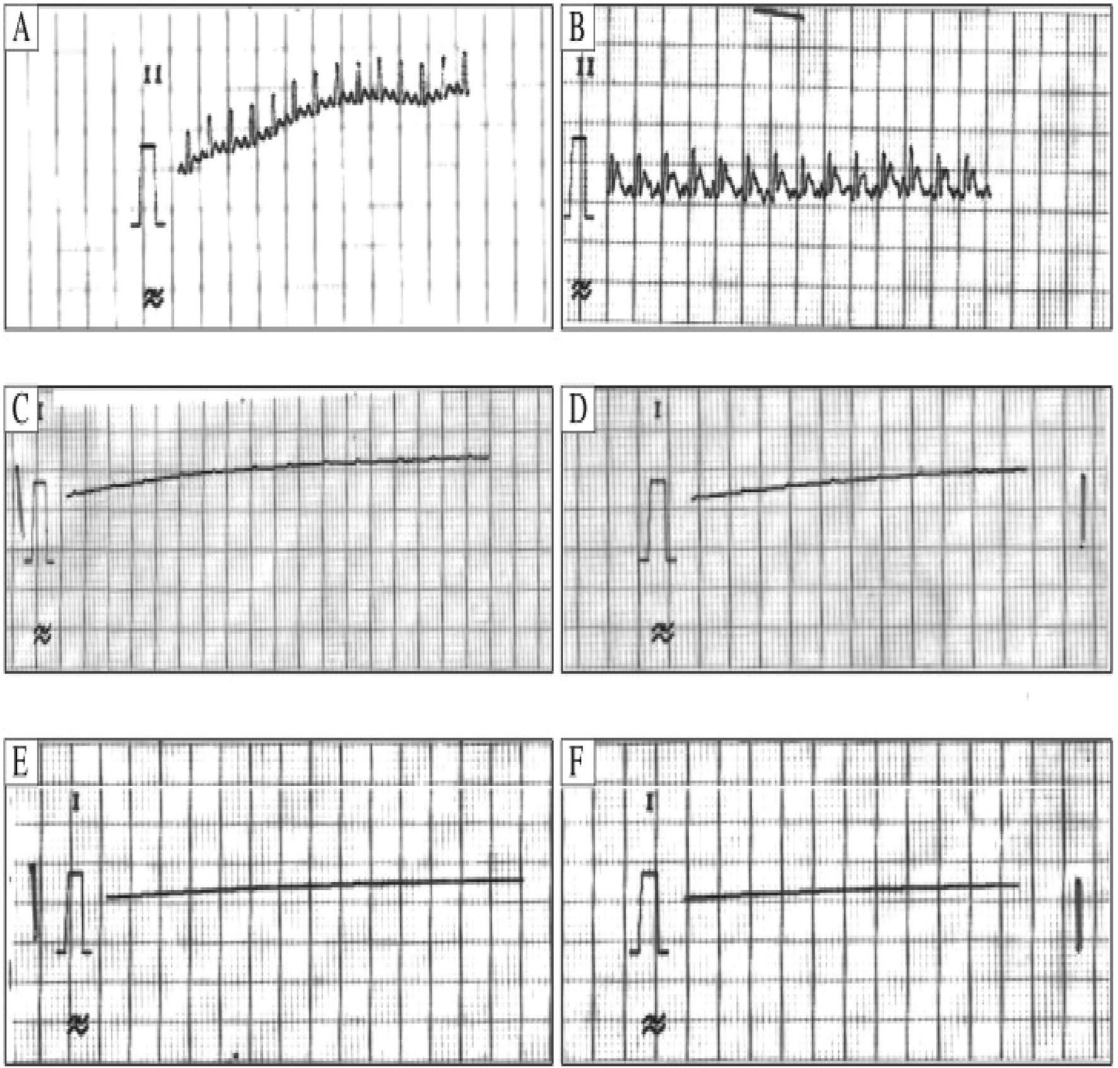
A sample ECG for Group II (10% CaCl2) taken at various times. In Group II [Arrhythmia control group 1, 10% CaCl2 (50 mg/kg) (n = 6)], the ECGs were found. Using 10% CaCl2 dose (at various times after intravenous injection). After administering 10% CaCl2, arrhythmia, cardiodepression, and death were noted at 20 min. (**A**) Normal reading (Control); (**B**) Arrhythmia caused by 10% CaCl2 dose at 0 min after administration; (**C**) Arrhythmia caused by 10% CaCl2 dose at 5 min after administration; (**D**) Arrhythmia caused by 10% CaCl2 dose at 10 min after administration; (**E**) Complete cardiodepression caused by 10% CaCl2 dose at 15 min after administration; (**F**) Mortality caused by 10% CaCl2 dose at 20 min after administration. . (Sharma AK, Kishore K, Sharma D, Srinivasan BP, Agarwal SS, Sharma A, Singh SK, Gaur S, Jatav VS. Cardioprotective activity of alcoholic extract of Tinospora cordifolia (Wild.) Miers in calcium chloride-induced cardiac arrhythmia in rats. Journal of Biomedical Research. 2011 Jul 1;25(4):280–6. PMID: 23554702. 10.1016/S1674-8301(11)60038-9))^233^.

**Figure 9:**
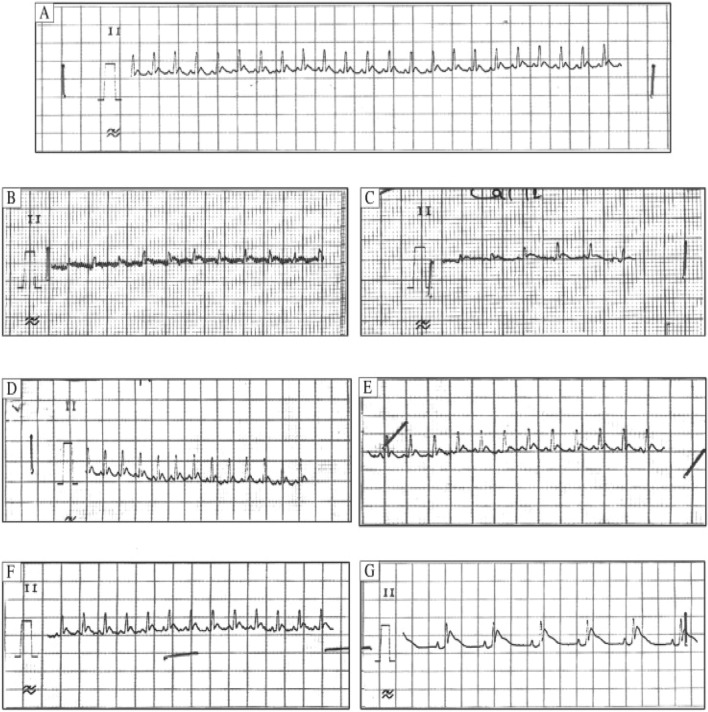
Shows an ECG that is representative of the several groups (Group I to Group VIII, n = 6) (**A**) Standard reading (Control); (**B**) Group III: "5% CaCl2 (25 mg/kg)" for arrhythmia management; (**C**) "CaCl2 (5%)-induced arrhythmia + alcohol (95%, 0.5 ml, IV); (**D**) Group V: Verapamil (5 mg/kg, IV) with CaCl2 (5%)-induced arrhythmia; (**E**) Tinospora cordifolia dried ethanolic extract dissolved in saline plus Group VI CaCl2 (5%)-induced arrhythmia.Tinospora cordifolia dried ethanolic extract diluted in saline (150 mg/kg, iv); Group VII CaCl2 (5%)-induced arrhythmia + Tinospora cordifolia extract (250 mg/kg, iv); Group VIII CaCl2 (5%)-induced arrhythmia + Tinospora cordifolia dry ethanolic extract (450 mg/kg, iv) (n = 6). ). (Sharma AK, Kishore K, Sharma D, Srinivasan BP, Agarwal SS, Sharma A, Singh SK, Gaur S, Jatav VS. Cardioprotective activity of alcoholic extract of Tinospora cordifolia (Wild.) Miers in calcium chloride-induced cardiac arrhythmia in rats. Journal of Biomedical Research. 2011 Jul 1;25(4):280–6. PMID: 23554702. 10.1016/S1674-8301(11)60038-9) )^233^.

**Figure 10:**
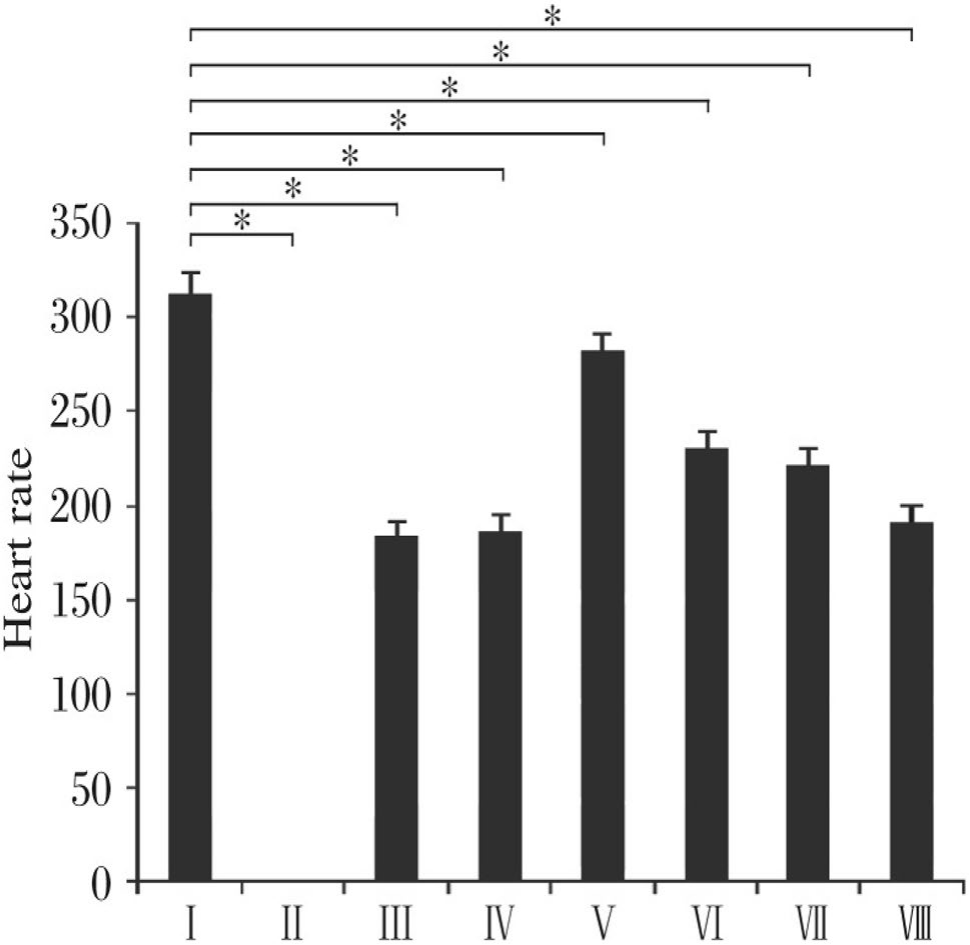
Shows a comparison of the effects of verapamil and Tinospora cordifolia extract on heart rate. *P 0.05 indicates statistical significance compared to control (Group I). (n = 6). CaCl2 (5%) + verapamil (5 mg/kg, iv); Group VI: CaCl2 (5%) + Tinospora cordifolia (150 mg/kg, iv); Group VII: CaCl2 (5%) + Tinospora cordifolia (250 mg/kg, iv); Group VIII: CaCl2 (5%) + Tinospora cordifolia (450 mg/kg, iv).Group I: control. (Sharma AK, Kishore K, Sharma D, Srinivasan BP, Agarwal SS, Sharma A, Singh SK, Gaur S, Jatav VS. Cardioprotective activity of alcoholic extract of Tinospora cordifolia (Wild.) Miers in calcium chloride-induced cardiac arrhythmia in rats. Journal of Biomedical Research. 2011 Jul 1;25(4):280–6. PMID: 23554702. 10.1016/S1674-8301(11)60038-9))^233^.

The study, Tinospora cordifolia (T. cordifolia) alcohol extract was tested for its ability to prevent Cacl_2_-induced arrhythmia. Rats were given intravenous infusions of Cacl_2_ (25 mg/kg) to induce arrhythmia. Then, T. cordifolia extract (150, 250, and 450 mg/kg) and verapamil (5 mg/kg, iv) were administered to the animals. Electrocardiogram in Lead II was seen. We measured the levels of potassium, sodium, and calcium in the plasma. Verapamil decreased the heart rate by 9.70%, T. cordifolia at 150, 300, and 450 mg/kg by 26.30%, 29.16%, and 38.29%, respectively, while Cacl_2_ decreased the heart rate by 41.10% when compared to the control group. In rats given verapamil and T. cordifolia, the PQRST waves were restored to normal, and atrial and ventricular fibrillation were controlled. Cacl_2_ raised the levels of calcium and salt while lowering potassium in the blood. T. cordifolia boosted potassium levels while dose-dependently lowering calcium and salt levels. As a result, T. cordifolia can be employed in therapeutic settings to treat antiarrhythmic conditions such as atrial and ventricular fibrillation and flutter, as well as ventricular tachyarrhythmia (Figs. 8, 9, 10)^233^.

### Electrically triggered arrhythmia

Serial electrical stimulation results in the development of flutter and fibrillation, which can resemble some of the most prevalent arrhythmias with clinical relevance. It is possible to evaluate the flutter threshold or the ventricular multiple response thresholds in dogs under anaesthesia, either before or after the test drug is supplied.

Limitations-Comments:

Dogs: Ventricular Fibrillation Electrical Threshold: Atria & ventricular thresholds were assessed using a variety of electrical pacing techniques, including sequential pulse pacing.

Dogs: Programmed Electrical stimulation induced Arrhythmia: sustained ventricular tachycardia and ventricular fibrillation.

Male mongrel dogs: Dog Model of Sudden Coronary Death: Coronary artery stenosis investigations into the onset of lethal arrhythmia and ventricular ectopy are conducted using recordings from the cardiocassette evaluation of tachyarrhythmias.

Rats, Dogs: Exercise-related ventricular fibrillation: Coronary artery stenosis of exercise on the treadmill.

#### Ventricular fibrillation electrical threshold

When comparing antifibrillators, consider the maximum rate at which the atria can respond to stimulation. Ventricular thresholds were assessed using a variety of electrical pacing techniques, including sequential pulse pacing, continuous 50 Hz pacing, and single pulse pacing. After intraperitoneal administration of pentobarbital sodium (35 mg/kg) to anesthetize dogs (8–12 kg), they are kept alive by artificial respiration.'s chest is opened, the heart is suspended over the pericardium, and blood pressure and temperature measurements are taken. The SA node is compressed and a Teflon disk sutured to the anterior surface of the left ventricle is implanted with the Ag–AgCl pacing electrode^234^. The pilot electrode is supplied with a constant anode current for 400 ms (3 ms squared). A computerized stimulator is used to program the electrical stimulation. A recording electrode is mounted outside each chamber. The ventricular fibrillation threshold (VFT) is determined by giving a value between 0.2 and 1.8-s train of 100 ms 50 Hz pulses for every 18 primary motor stimuli. VFT is the minimum pulse train current required to produce sustained VF. The heart is rapidly defibrillated at the onset of ventricular fibrillation and then has 15 to 20 min to return to a controlled state. The femoral vein is used to administer medication. VFT is assessed both before and after study drug administration and results are compared using Student's t-test^235^.

#### Programmed electrical stimulation induced arrhythmia

Dogs (8–12 kg) received 30 mg/kg sodium pentobarbital intravenously prior to anesthesia by mechanical ventilation. The cannula is inserted into the external jugular vein. A thoracotomy is performed between the fourth and fifth ribs to expose the heart.The left anterior descending coronary artery (ADL) is the only one. A 20 gauge hypodermic needle is placed over the LAD and a ligature is placed around the artery and needle. Withdrawing the needle creates a large stenosis in the vessel. The LAD infusion takes five minutes. Using a silicone rubber ring, the LAD is blocked for two hours to damage ischemic heart tissue. The critical stenosis is then maintained while the vessel is reperfused for an additional two hours. During the LAD reperfusion phase, an epicardial bipolar electrode is sutured to the interventricul septum, close to the region of obstruction. Silver disc electrodes that are subcutaneously implanted are used to track the ECG. The chest is reopened after 6–9 days, and planned electrical stimulation is carried out using an electrode implanted in a noninfarcted location. After 15 pacing stimuli, a 200 ms pacing stimulus is given as a final stimulus. Animals with persistent ventricular tachycardia and ventricular fibrillation are used in the investigation. Heart rate and ECG intervals are measured prior to the start of the planned electrical stimulation. The critical stenosis is then maintained while the vessel is reperfused for an additional two hours. During the LAD reperfusion phase, an epicardial bipolar electrode is sutured to the interventricular septum near the site of obstruction. Silver disc electrodes implanted under the skin are used for ECG monitoring. The chest is reopened after 6 to 9 days and elective electrical stimulation is given with an electrode implanted at a non-infarcted site. After 15 pacing stimuli, a stimulus lasting 200 ms is delivered as the last stimulus.Animals with sustained ventricular tachycardia and ventricular fibrillation are used for the study. Heart rate and ECG intervals are measured before programmed electrical stimulation begins. The study drug will then be administered after 30 min. The minimum current required to induce sustained VF is recorded before and after study drug administration, and the mean results of 10 experiments are compared using Student's t-test^234^.

#### Dog model of sudden coronary death

Sudden coronary death is one of the leading causes of death in industrialized countries. This dog model is used to investigate protection against sudden cardiac death. Male mongrel dogs weighing 14 to 22 kg are given intravenous pentobarital sodium (30 mg/kg) for sedation. The cannulas are inserted into the animal's trachea and maintained by artificial respiration. A cannula is inserted into the jugular vein to inject the test substance/saline solution.Once the chest is opened to show the heart, the left anterior descending coronary artery (LAD) is isolated and a 20G needle placed over it. Once the ligature is tied across the artery and a key stenosis develops, the needle is withdrawn. For severe strictures, the LAD is closed with a silicone rubber ring for two hours, followed by reperfusion for an additional two hours. Bipolar stainless steel epicardial electrodes and bipolar dip electrodes are sutured to the left atrial appendage and interventricular septum, respectively, to stimulate the left atrium. Two equivalent stainless steel electrodes are sutured to the wall of the left ventricle, one at the LAD position distal to the block and the other at the position of the left peripheral coronary artery (LCX). A silver-coated electrode is placed adjacent to the heart surface and penetrates through the wall into the LCX lumen. The silver disc electrodes for ECG monitoring are inserted via implants under the skin. The surgical incision is then closed and the animal is allowed to heal. After recovery, the animals receive the study drug. A direct anode current of 15 A from a 9-V nickel–cadmium battery connected to a subcutaneously implanted puck electrode is used to record a lead ECG II for 30 s every 15 min on a cardiac cassette recorder. If ventricular fibrillation or constant anodic current occurred for 24 h, the animals were sacrificed. After the hearts are removed, the thrombus mass in the LCX is removed and weighed. The heart is divided into slices and stained with tetrazolium triphenyl chloride (TTC stain) to determine where the infarction occurred. Investigations into the onset of lethal arrhythmia and ventricular ectopy are conducted using recordings from the cardiocassette evaluation of tachyarrhythmias, both sustained and not^235^.

### Exercise-related ventricular fibrillation

Coronary artery stenosis can be used in exercise-based studies that better mimic the circumstances of patients with coronary artery disease. This model can be used to assess how antiarrhythmic drugs affect various cardiovascular parameters during exercise and ischemia testing. Hybrid hearts weighing 15–19 kg are exposed after anesthesia with sodium pentobarbital (10 mg/kg, intravenously) and supported by the pericardial mount. The left circumflex artery is surrounded by a hydraulic obturator and a 20 MHz pulsed Doppler flow transducer. A pair of silver-plated insulated wires was used to suture the epicardial surfaces of the left and right ventricular electrograms, and a Gould biotachymeter was used to measure heart rate. In the next step, the left ventricle is punctured with a solid-state pressure transducer that has already been calibrated. The left anterior descending coronary artery is then partially occluded for 20 min before ligating. Leads from the cardiovascular system were inserted under the skin and tied around the animal's neck. To relieve the pain, the animals are given antibiotics and painkillers. Three to four weeks after the onset of myocardial ischemia, animals learned to sit still on a laboratory bench. Then run on a motorized treadmill during this healing period. VF sensitivity is then assessed on a motorized treadmill. The animals walked at 6.4 km/h (0% incline) for the first three minutes of treatment. The odds increase by 0%, 4%, 8%, 12% and 16% every three minutes. In the last minute of exercise, the treadmill is stopped, the left circumflex artery occluded, and the blockage maintained for an additional minute (total occlusion time 2 min). If the animal loses consciousness, an electrical defibrillator is used. In the case of ventricular fibrillation, the obstruction is released. The stress plus ischemia test is performed after pre-treatment with the test drug and the results are compared to those of the control group (saline).The effective refractory period is calculated the following day using the Medtronic Model 5325 programmable pacemaker, both at rest and during myocardial ischemia. In addition, the effect of the test chemical on coronary blood flow is measured using a flow meter^236^. Rate of change of left ventricular pressure and other hemodynamic data are recorded with a Gould Model 2800 S eight-channel recorder. Using analysis of variance, the response of reactive hyperemia to each refractory block is assessed after averaging the results. To investigate how a pharmacological intervention affects the occurrence of arrhythmias, the chi-square test with Yate's correction was performed.

### Mechanically generated arrhythmia

Arrhythmias can be caused by ischemia or reperfusion. Different stages of arrhythmias can be studied by coronary artery ligation or ischemia-induced infarction. The two-stage coronary ligation procedure focuses primarily on late cardiac arrhythmias. The impact on reperfusion arrhythmias can be assessed in many ways.

Limitations-Comments:

Rats: Reperfusion Arrhythmia in Rats: Ventricular arrhythmia and myocardial infarction by ligation of the left major coronary artery in the course of the ligation and subsequent reperfusion.

Dogs: Reperfusion Arrhythmia in Dogs: Ventricular arrhythmia and myocardial infarction by ligation of the left major coronary artery in the course of the ligation and subsequent reperfusion.

Mice, Rats, Dogs, Guinea Pigs, Rabbits, Monkeys: Genetically Prone Arrhythmias. Limitations: Limited to embrogenic gene manupulations, ie. Gene mutations and gene knockout varients.

#### Reperfusion arrhythmia in rats

Ventricular arrhythmia and myocardial infarction are the outcomes of ligation of the left major coronary artery. In the course of the ligation and subsequent reperfusion, an electrocardiogram is taken. In cardiac slices, p-nitro blue tetrazolium chloride staining is used to quantify the amount of infarcted tissue. Pentobarbitone sodium (60 mg/kg) is administered intraperitoneally to anaesthetize Sprague–Dawley(350-400gm) rats. The jugular vein is cannulated to provide test medications while the animal is kept on artificial breathing. Using a pressure transducer attached to a polygraph, blood pressure is taken from the carotid artery. The heart is visible when the chest is opened. In the case of infarct size investigations, the left coronary artery is found, ligated for 15 to 90 min, and then re-perfused for 30 min. Five minutes prior to the ligation, test medication is given. Throughout the entire experiment, peripheral blood pressure and ECG lead II are continually recorded. During the occlusion and reperfusion periods, the number of premature ventricular beats, ventricular tachycardia, and fibrillation are counted^237,238^. The animal is sacrificed at the conclusion of the reperfusion phase, and the infarct size is measured using TTC (p-nitro blue tetrazolium trichloride) staining. To visualise the infarct tissue (blue/violet, stained healthy tissue, unstrained necrotic tissue) the heart is dissected, sliced into transverse Sects. (1 mm thick), and stained with TTC produced in Sorensen phosphate buffer containing 100 mM, L-maleate. Slices are imaged, and the baby area is calculated using planimetry using all of the slice projections. Comparing drug-treated animals- variation in hemodynamic parameters and infarct size to control values.

#### Reperfusion arrhythmia in dogs

Dogs with coronary artery ligation may experience elevated blood pressure, heart rate, heart contractility, left ventricular end diastolic pressure, and ventricular arrhythmias, particularly during the reperfusion period. The anaesthetic thio-butobarbital sodium (30 mg/kg intraperitoneally) is administered to dogs (20–25 kg) to induce anaesthesia. The dogs are then maintained on intravenous chloralose (20 mg/kg) and 250 mg/kg urethane intravenously, followed by the subcutaneous delivery of 2 mg/kg(morphine). Animal is then kept on an artificial breathing system: For the purpose of administering the test substance, the saphenous vein is cannulated, and lead II of the ECG is constantly monitored. A pressure transducer is attached to a cannulated femoral artery to measure blood pressure. The left ventricular pressure curves are used to calculate heart rate and left ventricular end diastolic pressure. An increase in left ventricular pressure is used to evaluate myocardial contractility. Rats were used in the experiment in a similar manner. 90 min are spent ligating the coronary artery. The test substance is given twenty minutes before ligation. The animal is reperfused for 30min^239,240^. During the course of the experiment, all of the mentioned parameters are recorded.

Comparing drug-treated animals to vehicle controls reveals changes in metrics (mortality, hemodynamics, and arrhythmia).

#### Two stage coronary ligation in dogs

Methohexitone sodium (10 mg/kg) is administered intravenously to anaesthetize dogs (8–11 kg) and keep them breathing artificially. The heart is visible when the chest is opened. Two phases of coronary ligation are carried out after the location of the left coronary artery. A 21- gauge needle and two ligatures are put around the artery. The needle is removed after the initial ligature has been tied around the artery. The second ligature is firmly secured around the artery 30 min later. After the second ligature has been knotted for 30 min, the chest is closed in layers and the dog is given time to recover. Arrhythmias start to appear after 24 and 48 h following ligation and disappear after 3–5 days. The atrial electrogram, lead II ECG, and mean blood pressure are all recorded. Following coronary artery ligation, test medications are administered as an infusion for 10 minutes^241,242^. Animals given drugs are compared to vehicle controls for changes in metrics (mortality), hemodynamics, and arrhythmia).Boyden and Hoffman's canine model, which causes tricuspid regurgitation and right atrial enlargement by banding the pulmonary artery, may also have a clinical analogue in people who have both tricuspid regurgitation and chronic obstructive pulmonary disease^243^. Instead of an anatomical barrier, a functional zone of blocking and area of sluggish conduction create the conditions for re-entry in these dogs. The first documented model of canine atrial flutter using sterile pericarditis also shows functional entrance^244,245^. Atrial flutter frequently occurs in patients after cardiac surgery, and this may be associated to postoperative sterile pericarditis, which is why this model was created.

## Genetically induced arrhythmia

It has been reported that a group of German shepherd dogs have inherited ventricular arrhythmias and are more prone to abrupt demise, which typically happens while they are sleeping or at rest following an active or exciting day. These canines can be used to evaluate possible antiarrhythmic medications. Although there is commonly noticeable T wave notching on the ECG, the QT interval is not prolonged. The ventricular tachycardias are quick polymorphic, follow lengthy R-R intervals, and are almost certainly brought on by early depolarizations of the Purkinje system, which activate the triggered activity. Both the transient outward current's density and its time constant of inactivation are decreased in the epicardial myocytes^246^. Additionally, there are deficiencies in cardiac sympathetic denervations. This dog model initially reminds one of the congenital long QT- syndrome, which is characterised by bradycardia-induced polymorphic ventricular tachycardia, rapid mortality, and genetic abnormalities in ion channels that control repolarization. The dogs do not, however, exhibit a prolonged QT interval. No I_O_ deficiency has been reported in patients with long QT syndrome^242–245^. However, patients have been reported to have polymorphous ventricular tachycardia (Torsadede pointes) with a normal QT interval, suggesting that this animal model may have a human counterpart.

Limitations-comments:

Genetic arrhythmia disorders are either caused by or associated with identifiable gene mutations. Genetic arrhythmia syndromes can be subdivided into channelopathies without structural heart disease (eg, CPVT, long QT syndrome [LQTS], Brugada Syndrome) and genetic arrhythmia syndromes associated with structural heart disease (eg, hypertrophic cardiomyopathy [HCM], dilated cardiomyopathy [DCM], action potential duration [ARVC], AF). Tables 2 and 3 list published animal models of genetic arrhythmia syndromes. Most animal arrhythmia models are mice, given the ease of genetic manipulation. Despite differences between rodent and human cardiac electrophysiology (Fig. 2), mouse models have enabled the study of human genetic diseases, identification of pathogenic mutations, characterization of disease pathophysiology, and testing/screening of therapeutic interventions. Advances in gene editing technology such as CRISPR/Cas9 have provided faster, easier, and cheaper methods to develop genetic models in animals and cells. Compared with cellular models such as human-induced pluripotent stem cells, animal models provide a distinct advantage in modeling cardiac arrhythmias where the anatomy of the heart is relevant^247^. In the following section, each of the major genetic arrhythmia syndromes are introduced, followed by examples of animal models that have informed disease pathophysiology and treatment approaches.

**Table 2:** Animal models of genetic arrhythmia channelopathies.

Disease, type, animal	Human ortholog gene (protein)	Mutation	Notes
CPVT			
CPVT1			
Mouse	*RYR2* (RyR2)	R4496C ±	Catecholamine/exercise-induced arrhythmia; phenotype not as penetrant as some other models
Mouse	*RYR2* (RyR2)	V2475F ±	Homs are embryonic lethal, catecholamine-induced arrhythmia
Mouse	*RYR2* (RyR2)	R2474S ±	Seizures, exercise-induced arrhythmia, sudden death
Mouse	*RYR2* (RyR2)	P2328S ±	Iso-induced PVCs, VT, VF
Mouse	*RYR2* (RyR2)	R2474S/V3599K	GoF + LoF; protective- no arrhythmia
Mouse	*RYR2* (RyR2)	R4496C + / − with E4872Q + / −	GoF + LoF; protective- no arrhythmia
Mouse	*RYR2* (RyR2)	L433P + / − , N2386I + / −	Mutations from patients with CPVT; no report of CPVT in mouse models; mice also develop AF with pacing protocol
Mouse	*RYR2* (RyR2)	R176Q ±	CPVT-like phenotype; AF
Mouse	*RYR2* (RyR2)	exon3del ±	Homs embryonic lethal, hets did not develop CPVT; patients with exon3del have exercise-induced VT
C. elegans	*RYR2* (RyR2), *CASQ2* (Casq2)	R4743C, Casq2 KO	Enables optogenetic pacing, observed defect with mutations
CPVT2			
Mouse	*CASQ2* (Casq2)	KO	Severe CPVT, ultrastructure changes, catecholamine/exercise- induced arrhythmia
Mouse	*CASQ2* (Casq2)	D307H/D307H, D309deltaE9/D309deltaE9 (Casq2-/-)	Both mutations result in loss of Casq2 protein, catecholamine/exercise-induced arrhythmia
Mouse	*CASQ2* (Casq2)	Ventricular/Purkinje KO	Subtissue-selective knockout
Mouse	*CASQ2* (Casq2)	Ventricular/Purkinje KO	Subtissue-selective knockout
Mouse	*CASQ2* (Casq2)	R33Q/R33Q	Reduction of Casq2 protein, ultrastructure changes, arrhythmia
Mouse	*CASQ2* (Casq2)	K180R ±	Autosomal dominant inheritance; iso-induced arrhythmias
CPVT4			
Zebrafish	*CALM* (CaM)	N53I and N97S	Increased iso-induced HR (indicated dominant negative effect)
Zebrafish	*CALM* (CaM)	E105A	Arrhythmias, tachycardia, altered RyR2 binding
CPVT5			
Mouse	*TRDN* (Triadin)	KO	Ultrastructure changes; iso-induced arrhythmias; overlap syn- drome with LQT?
CPVT?			
Mouse	*KCNJ2* (Kir2.1)	R67Q + / − , cardiac-specific	Structurally normal heart, iso-induced VT, no LQT
CRDS			
Mouse	*RYR2* (RyR2)	A4860G ±	VF with sympathetic stimulation, homs embryonic lethal
Mouse	*RYR2* (RyR2)	D4646A ±	RyR2 Ca2 + release deficiency syndrome (CRDS); homs are embryonic lethal
LQTS			
LQT1			
Mouse	*KCNQ1* (Kv7.1)	KO	Deaf, shaker/waltzer phenotype; LQT
Mouse	*KCNQ1* (Kv7.1)	Truncated isoform, cardiac-specif- ic overexpression	LQT, sinus node dysfunction, occasional AV block
Mouse	*KCNQ1* (Kv7.1)	A340V − / −	Homs have LQT, hets do with gene dose dependence; PVCs after feeding (linked to diabetes)
Rabbit	*KCNQ1* (Kv7.1)	Y315S cardiac-specific overex- pression	LQT; sympathetic stimulation induces EADs and VT; rabbits die within 3 weeks of AV node ablation
LQT2			
Rabbit	*KCNH2* (Kv11.1/hERG)	G628S cardiac-specific overex- pression	LQT, spontaneous PVT, sudden death; prolonged APD
Zebrafish	*KCNH2* (Kv11.1/hERG)	I462R, M521K	2:1 block (phenotype)
Zebrafish	*KCNH2* (Kv11.1/hERG)	I59S − / −	2:1 block (phenotype); prolonged APD; EADs
Zebrafish	*KCNH2* (Kv11.1/hERG)	Morpholino KD of WT + expres- sion of hERG ± mutants	Tested 40 pathogenic and 10 nonpathogenic hERG mutants in the zERG background
LQT3			
Mouse	*SCN5A* (Nav1.5)	ΔKPQ/ +	1505–1507 deletion; prolonged QT/QTc; prominent T wave; prolonged APD; arrhythmias; sudden death
Mouse	*SCN5A* (Nav1.5)	ΔQKP/ +	1507–1509 deletion; long QTc, wide QRS, AV block; spontane- ous PVCs, VT, and VF with sudden death; no atrial arrhythmias
Mouse	*SCN5A* (Nav1.5)	N1325S cardiac-specific overex- pression	LQT; arrhythmia; sudden death; also other non-LQT3 features like shorter PR and elevated heart rate
Mouse	*SCN5A* (Nav1.5)	1795insD	LQT and Brugada in family; homs embryonic lethal; sinus node dysfunction, conduction slowing, bradycardia, and LQT
Guinea pig	*SCN5A* (Nav1.5)	Cellular model, isolated cells tx	Isolated cardiomyocytes treated with anthopleurin; rescued by mexillitine
Minor types of LQTS			
Mouse	*ANK2* (Ankyrin-B)	KO	LQT with HR deceleration, sinus node dysfunction
Mouse	*ANK2* (Ankyrin-B)	±	Bradycardia, variable HR, slow conduction, AV dissociation, long QTc; iso/exercise-induced PVT & death
Mouse	*KCNA1* (Kv1.1)	N-term fragment overexpression	Long QTc; spontaneous PVC, couplets, ventricular tachycardia
Mouse	*KCNA5* (Kv1.5)	W461F cardiac-specific overex- pression	Long QTc
Mouse	*KCNB1* (Kv2.1)	N216 cardiac-specific overex- pression	Long QTc
Mouse	*KCND2* (Kv4.2)	W362F cardiac-specific overex- pression	Subtle bradycardia, prolonged QTc
Mouse	KCND2 × KCNA4	W362F × KO	QRS widening, prolonged QTc, AV block/dropped beats, spon- taneous ventricular arrhythmia
Rabbit	*KCNE1* (minK)	G52R dominant negative overex- pression	Long QTc; drug-induced arrhythmia (Torsades)
Mouse	*KCNE1* (minK)	KO	LQT with HR deceleration
Mouse	*KCNE2* (MiRP1)	KO	Long QTc with age, mice had hyperkalemia
Mouse	*KCNE3* (MiRP2)	KO	Females have long QTc at 9 mo (hyperaldosteronism); increased susceptibility to IR arrhythmias
Mouse	*KCNJ2* (Kir2.1)	KO	Bradycardia, LQT
Mouse	*KCNJ2* (Kir2.1)	T75R cardiac-specific overex- pression	Long QTc; spontaneous VT; iso-induced PVCs, VT, atrial flutter/fibrillation
Mouse	*KCNIP2* (KChIP2)	KO	Significant reduction in Ito; elevated ST segment, atrial flutter and VT with PES; prolonged APD in cells
Mouse	*CACNA1C* (Cav1.2)	− / − , ±	KO embryonic lethal, hets survive and are just like WT
Mouse	*CACNA1C* (Cav1.2)	G406R cardiac-specific overex- pression	Long QTc, exercise-induced PVCs and Torsades; crossing with AKAP150 KO protected against all phenotypes of the G406R mutant
Zebrafish	*CALM* (CaM)	D129G	Bradycardia; conduction abnormality; LQT
Mouse	*SCN1B* (Scn β1)	KO	Bradycardia, prolonged APD/QTc, slowed repolarization; sodium channel expression increased
Mouse	*SLC18A2* (VMAT2)	±	LQT
Mouse	*ATP1A3* (NaK ATPase α3)	Human isoform overexpression	LQT, steeper QT rate dependence, T wave alternans, VT
Short QT syndrome (SQTS)			
SQTS1			
Rabbit	*KCNH2* (Kv11.1/hERG)	N588K cardiac-specific overex- pression	Shortened APs and QTc, normal T wave height, ex vivo perfused hearts inducible VT/VF
Zebrafish	*KCNH2* (Kv11.1/hERG)	L499P	SQT
SQTS8			
Zebrafish	*SLC4A3* (AE3)	Knockdown	SQT and systolic duration; WT SLC4A3 expression rescued phenotype, but R370H did not
Mouse	*SLC8A1* (NCX)	KO	SQT
Mouse	*CAV3* (Caveolin 3)	WT cardiac-specific overexpres- sion	Short QTc, bradycardia, prolonged PR
Kangaroo	Unknown	None reported	Kangaroos have LV hypertrophy, SQT, and are highly susceptible to VF and sudden death, especially under light anesthesia
BrS			
Pig	*SCN5A* (Nav1.5)	E558X ±	Conduction abnormalities, QRS widening, reduced conduction velocity, ex vivo hearts have increased susceptibility to VF
Mouse	*SCN5A* (Nav1.5)	±	KO is embryonic lethal; hets have sick sinus, slowed conduction, pacing-induced VT; QTS widening and fibrosis with age
Mouse	*SCN5A* (Nav1.5)	1795insD	LQT and BrS in family; hom mice embryonic lethal; sinus node dysfunction, conduction slowing, bradycardia, and LQT
Mouse	*SCN5A* (Nav1.5)	ΔSIV/ +	C-term truncation; SA, AV, and His conduction slowing; one hu- man patient with V2016M diagnosed with Brugada

AF indicates atrial fibrillation; APD, action potential duration; AV, atrioventricular; caff, caffeine; BrS, Brugada Syndrome; CPVT, catecholaminergic polymorphic ven- tricular tachycardia; CRDS, calcium release deficiency syndrome; DAD, delayed afterdepolarization; DKO, double knockout; DN, dominant negative; EAD, early afterde- polarization; GoF, gain of function; het, heterozygous; HF, heart failure; hom, homozygous; HR, heart rate; IR, ischemia–reperfusion; iso, isoproterenol; KO, knockout; LoF, loss of function; LQTS, long QT syndrome; MVT, monomorphic ventricular tachycardia; NSVT, nonsustained ventricular tachycardia; PES, programmed electrical stimula- tion; PVC, premature ventricular complex; PVT, polymorphic ventricular tachycardia; RBBB, right bundle branch block; SVT, supraventricular tachycardia; VF, ventricular fibrillation; and WT, wild type.

**Table 3:** Animal models of genetic arrhythmia syndromes with structural heart disease.

Disease, type, animal	Human ortholog gene (protein)	Mutation	Notes
Arrhythmogenic right ventricular cardiomyopathy			
Desmosomal			
Mouse	*JUP* (Plakoglobin)	±	Homs embryonic lethal; hets develop arrhythmia, RV structure change, etc
Mouse	*JUP* (Plakoglobin)	c.2037-2038delTG	Truncated form, LoF, mortality, fibrosis
Mouse	*JUP* (Plakoglobin)	23654del2 overexpression	Repressed Wnt signaling; fibrosis, dysfunction, death
Zebrafish	*JUP* (Plakoglobin)	Knockdown	Smaller heart size, blood reflux between chambers, reduced heart rate
Zebrafish	*JUP* (Plakoglobin)	2057del2	Structural change, mortality
Mouse	*DSP* (Desmoplakin)	±	Homs are embryonic lethal; hets have fibrosis, dysfunction, arrhyth- mias; Wnt signaling implicated
Mouse	*DSP* (Desmoplakin)	Conduction-specific KO (HCN4)	Migrating atrial pacemakers, sinus rhythm dysfunction, all without cardiac remodeling
Mouse	*DSP* (Desmoplakin)	R2834H cardiac-specific over- expression	Cardiac fibrosis, other small changes, no dysplasia
Mouse	*PKP2* (Plakophilin 2)	±	Homs embryonic lethal; hets histologically normal, but electrical re- modeling, ultrastructure changes, arrhythmia susceptibility
Mouse	*PKP2* (Plakophilin 2)	S329X cardiac-specific over- expression	Histologically normal, but electrical remodeling, ultrastructure chang- es, arrhythmia susceptibility
Mouse	*PKP2* (Plakophilin 2)	Inducible cardiac KO	Fibrosis, arrhythmia, remodeling, death, reduced lvEF
Mouse	*PKP2* (Plakophilin 2)	R735X AAV9-expression	RV dysfunction with exercise
Zebrafish	*PKP2* (Plakophilin 2)	Knockdown	Structural defects, signaling reduced
Zebrafish	*DSC2* (Desmocollin 2)	Knockdown	Altered ultrastructure, contractile dysfunction
Mouse	*DSC2* (Desmocollin 2)	G790del/G790del, + /G790del	No phenotype
Mouse	*DSC2* (Desmocollin 2)	WT overexpression	Necrosis, acute inflammation and patchy cardiac fibrotic remodeling
Mouse	*DSG2* (Desmoglein 2)	KO	Embryonic lethal
Mouse	*DSG2* (Desmoglein 2)	Cardiac-specific KO	Dilation, fibrosis, electrophysiological remodeling
Mouse	*DSG2* (Desmoglein 2)	N271S cardiac-specific over- expression	Biventricular dilatation; spontaneous ventricular arrhythmias, cardiac dysfunction, sudden death
Mouse	*DSG2* (Desmoglein 2)	Q558X	Fibrosis, decrease in desmosomal size and number, and reduced Wnt signaling
Mouse	*PPP1R13L* (IASPP)	KO	Inducible Ppp1r13l knockout mouse model, dilation, arrhythmia, sud- den death
Mouse	*SORBS2* (ArgBP2)	KO	QRS widening, RBBB, spontaneous PVCs, NSVT, VT
Nondesmosomal			
Mouse	*RYR2* (RyR2)	R176Q ±	Patients with R176Q also have T250M and present with ARVC; but mouse 176Q alone gives CPVT-like phenotype; no ARVC/D, but slight end-diastolic changes
Mouse	*RYR2* (RyR2)	Inducible cardiac-specific KO	Sinus bradycardia, block, ventricular tachycardia, sudden death
Mouse	*ITGB1* (Integrin β1)	Inducible cardiac specific KO	Beta1d isoform is reduced in ARVC patients; KO mice had iso/caff- inducible VT
Mouse	*TMEM43* (Luma)	S358L ±	Fibro-fatty replacement, structural abnormalities, arrhythmia, sudden death
Mouse	*ILK* (ILK)	Cardiac-specific KO	Arrhythmia, sudden death; arrhythmogenic cardiomyopathy in some patients with missense mutations in ILK
Mouse	*RPSA* (LAMR1)	1031 bp insertion (spontane- ous)	ARVC; conduction abnormalities (QRS widening); no examination of inducible arrhythmias
Unknown			
Dog	Unknown	Autosomal dominant inheri- tance	Boxers; fatty replacement of RV myocardium, ventricular arrhythmia, syncope, sudden death
Dog	Unknown	Unknown	Weimaraner; syncope, ventricular arrhythmias, and sudden death, with histopathologic fatty tissue infiltration
Dog	Unknown	Unknown	English bulldog
Cat	Unknown	Unknown	SVT, VT, PVT, RBBB
Dilated cardiomyopathy (DCM)			
Mouse	*LMNA* (Lamin A/C)	±	DCM, arrhythmia
Mouse	*LMNA* (Lamin A/C)	H222P/H222P	Chamber dilation; slowed conduction, AV block, spontaneous PVCs
Mouse	*LMNA* (Lamin A/C)	N195K/N195K	Bradycardia, exit block, AV block, arrhythmia, sudden death
Mouse	*LMNA* (Lamin A/C)	G609G/G609G	Truncating splice variant; bradycardia, QRS widening, SA block, LQT
Pig	*LMNA* (Lamin A/C)	G609G/ +	Bradycardia, SA block, short QTc; spontaneous PVT, 3rd degree block at death
Zebrafish	*DES* (Desmin)	KO or aggregating mutant	Embryonic tachycardia, arrhythmia; however, no reports of electrophysi- ological changes or arrhythmias in two independent KO mouse models
Mouse	*DES* (Desmin)	R349P ±	Human R350P; DCM, ARVC; slowed conduction and AV block; spontaneous and induced atrial fibrillation, PVCs, and VT
Zebrafish	*ACTN2* (F-Actin)	Knockdown	DCM, bradycardia
Mouse	*LDB3* (ZASP)	S196L cardiac-specific over- expression	DCM, arrhythmia
Mouse	*CDH2* (Cadherin)	cardiac-specific KO	DCM, arrhythmia, conduction defects
Mouse	*LMOD2* (Leiomodin)	KO	DCM; LQT
Rat	*RMB20* (RMB20)	KO	DCM; QRS widening, AV block, susceptibility to arrhythmias with PES, sudden death
Mouse	*RMB20* (RMB20)	KO	DCM; slowed conduction, LQT; changes in ion channel and calcium- handling proteins; spontaneous calcium release in isolated cardio- myocytes
Mouse	*RMB20* (RMB20)	S637A/S637A	DCM; spontaneous AF, spontaneous VT/VF with syncope, sudden death; much more severe cardiomyopathy than KO
Mouse	*PLN* (Phospholamban)	R14del/R14del	Mice develop severe DCM; no arrhythmias in vivo; explanted hearts have induced ventricular arrhythmias
Mouse	*SCN5A* (Nav1.5)	Cardiac-specific knockdown	Slow conduction, sudden death
Mouse	*SCN5A* (Nav1.5)	S571E/S571E	Phosphomimetic CaMKII target; LV dilation; LQT, iso-induced PVCs and VT
Mouse	*SCN5A* (Nav1.5)	D1275N/ + or D1275N/D1275N	Homs have slow conduction, heart block, AF, VT, and DCM without significant fibrosis or myocyte disarray
Zebrafish	*SCN5A* (Nav1.5)	D1275N	Bradycardia, sinus pause, AV block, sudden death; no AF or VT observed
Pig	*DMD* (Dystrophin)	KO (exon52del)	Fibrosis, low voltage areas, and sudden death
Dog	*DMD* (Dystrophin)	X-linked DMD	Short PR interval, sinus arrest, spontaneous ventricular arrhythmias
Mouse	*DMD* (Dystrophin)	KO (mdx strain)	Tachycardia; short PR, QRS, and QTc
Mouse	*DMD* (Dystrophin)	KO (5cv strain)	Short PR interval, inducible VT
Mouse	*VCL* (Vinculin)	Cardiac-specific KO	Normal sinus rhythm, AV block, spontaneous PVT, sudden death (be- fore onset of DCM phenotype)
Mouse	*VASP* (VASP)	Cardiac-specific overexpression	Bradycardia, AV block, sudden death
Mouse	*REST* (NRSF)	DN cardiac-specific overex- pression	Prolonged PQ, AV block, spontaneous VT, sudden death (observed as VT/VF with asystole)
Dog	Unknown	Autosomal dominant	Doberman Pinscher, DCM with age, PVCs on Holter monitor
Dog	Unknown	X-linked recessive inheritance	Great Dane; DCM; AF
HCM			
Mouse	*TNNT2* (TnT)	I79N cardiac-specific	Elevated diastolic Ca with elevated HR; iso-inducible ectopy; spon- taneous NSVT; no hypertrophy or fibrosis
Mouse	*TNNT2* (TnT)	+ /ΔK210 and ΔK210/ΔK210	Cardiac enlargement; HF; TdP, VF, sudden death; homs worse than hets but both had phenotype
Mouse	*TNNT2* (TnT)	F110I, R278C, or slow skeletal isoform TG	Iso-induced PVCs and VT in mice with F110I or skeletal isoform; VT inducibility with PES in ex vivo hearts; R278C had no arrhythmias compared to control
Rat	*TNNT2* (TnT)	Trunc transgenic overexpression	VT, VF
Mouse	*TNNI3* (TnI)	G203S cardiac-specific over- expression	PR prolongation, conduction delay; no arrhythmias, but later shown to have AF
Mouse	*TNNI3* (TnI) × *MYH6* (α- MHC)	G203S × R403Q	Bradycardia, slow conduction (PR and QRS), long QTc, catechol- amine-induced VT
Mouse	*MYH6* (α-MHC)	R403Q/ +	Right axis deviation, prolonged ventricular repolarization and pro- longed sinus node recovery times; programmable VT more in males than females
Mouse	*MYH6* (α-MHC)	R403Q overexpression	
Mouse	*MYPBC3* (MyBP-C)	trunc/trunc	Arrhythmias with PES
Mouse	*MYPBC3* (MyBP-C)	KO	Prolonged QTc, spontaneous PVCs and VT
Cat	*MYPBC3* (MyBP-C)	A31P	HCM
Cat	*MYPBC3* (MyBP-C)	R820W	HCM
Mouse	*HRAS* (H-Ras)	Cardiac-specific overexpres- sion	Sinus arrest, idioventricular rhythm, VT, block, and AF; phenotype stronger and more penetrant in females
Mouse	*RYR2* (RyR2)	P1124L/P1124L	Mild HCM; bradycardia, iso/caff-induced VT
Mouse	*OBSCN* (Obscurin)	R4344Q/R4344Q	Tachycardia, spontaneous PVCs and VT; all without structural remodeling
Kangaroo	Unknown	Unknown	Kangaroos have LV hypertrophy, short QT intervals, and are highly sus- ceptible to VF and sudden death, especially under light anesthesia
AF			
SR Calcium Release			
Mouse	*RYR2* (RyR2)	L433P + / − , N2386I + / − , R2474S ±	Mutations from CPVT patients. Also develop AF with atrial PES
Mouse	*FKBP1B* (FKBP12.6)	KO	No ECG abnormalities or spontaneous arrhythmias; AF with PES
Mouse	*CASQ2* (Casq2)	KO	AF with PES
Mouse	*JPH2* (Junctophilin 2)	E169K/ + (non AF-associated A399S ctrl)	E169K identified in HCM family with AF; before hypertrophy- AF with PES only in E169K mice
Mouse	*CREM* (CREM)	IbΔC-X	Atrial dilation; 100% of mice developed paroxysmal and persistent AF with age
Mouse	*NLRP3* (NLRP3)	A350V/ +	Constitutively active; normal conduction, spontaneous PACs, pacing- induced AF
Mouse	*SLN* (Sarcolipin)	KO	Cellular APD prolongation; atrial fibrosis; AF with age
Ion Channel			
Mouse	*SCN5A* (Nav1.5)	F1759A ±	Atrial and ventricular enlargement, myofibril disarray, fibrosis and mi- tochondrial injury, and electrophysiological dysfunction
Mouse	*SCN5A* (Nav1.5)	ΔKPQ/ +	Atrial enlargement; increased susceptibility to AF with PES
Mouse	*KCNE1* (minK)	KO	Spontaneous AF
Mouse	*KCNA1* (Kv1.1)	KO	AF with PES
Mouse	*KCNJ2* (Kir2.1)	T75R cardiac-specific overex- pression	Long QTc; spontaneous VT; iso-induced PVCs, VT, atrial flutter/fibrillation
Mouse	*KCNE5* (MiRP4)	KO	Inducible PVCs, atrial arrhythmia, PVT
Mouse	*KCNN2* (KCa2.2)	− / − and ±	Sinus and AV node dysfunction, AF with PES
Structural			
Mouse	*PITX2* (PITX2)	±	Normal cardiac parameters except reduced transpulmonary flow (pulmonary valve narrowing), ex vivo hearts were more susceptible to atrial pacing-induced arrhythmia
Mouse	*TBX5* (TBX5)	KO, inducible	Spontaneous AF within 2 wk post-induction, substantial arrhythmo- genesis and cardiac remodeling starting after 3 wk, calcium-handling protein expression changes
Mouse	*GJA1* (Cx43)	G60S/ +	Highly susceptible to inducible AF
Mouse	*STK11IP* (LKB1)	KO	Spontaneous AF, AV block, atrial flutter, electrical and structural remodeling
Mouse	*STK11IP* (LKB1)	Inducible atrial-specific KO	Spontaneous AF
Mouse	*CALCR* (calcitonin re- ceptor)	KO	Atrial fibrosis, inducible AF
Goat	*TGFB1* (TGF-β1)	C33S cardiac-specific overex- pression	Constitutively active TGF-beta; atrial fibrosis, prolonged P wave, AF inducible with PES, no spontaneous or persistent AF
Mouse	*TGFB1* (TGF-β1)	C33S cardiac-specific overex- pression	Constitutively active TGF-beta; atrial fibrosis, atrial inducibility with PES
Mouse	*MAP2K4* (MKK4)	Atrial-specific KO	Regulator of TGF-beta; reduced P wave amplitude, spontaneous atri- al tachycardia, polymorphic atrial beats, AF induced ex vivo with PES
Mouse	*ACE* (ACE)	Cardiac-restricted expression	Atrial enlargement, mild fibrosis; low QRS voltage, spontaneous AF, sudden death (escape rhythm preceded death in observed cases)
Mouse	*JDP2* (JDP2)	Cardiac-specific overexpres- sion	QRS widening, AV block, spontaneous paroxysmal AF; atrial hyper- trophy, fibrosis
Dog	Unknown	Unknown	Great Danes with DCM; AF
Sick sinus syndrome			
Mouse	*SCN5A* (Nav1.5)	±	Bradycardia, slowed conduction, exit block
Mouse	*SCN3B* (Scn β3)	KO	Bradycardia, sinus conduction slowing, exit block, AV block
Mouse	*NOTCH1* (notch recep- tor 1)	Inducible intracellular domain (Notch activation)	Bradycardia, sinus pauses, reduced conduction velocity; atrial ar- rhythmias with PES; Nkx2-5, Tbx2, Tbx5 expression altered
Zebrafish	*SMO* (smoothened)	Unreported, homozygous	Bradycardia, reduced spontaneous hyperpolarizing current
Mouse	*SLC8A1* (NCX)	Atrial-specific KO	Bradycardia, no P waves, junctional escape rhythm (His)
Zebrafish	*SLC8A1* (NCX)	Truncation	Embryonic lethal; embryos have atrial fibrillation/bradycardia/tachycar- dia; some VF but mostly silent ventricle; cardiac morphological defects
Mouse	*HCN1* (HCN1)	KO	Bradycardia, sinus dysrhythmia, prolonged SA node recovery time, increased SA conduction time, and recurrent sinus pauses
Mouse	*HCN2* (HCN2)	KO	Sinus dysrhythmia
Mouse	*HCN4* (HCN4)	KO	Global or cardiac HCN4 − / − embryonic lethal, but embryos have sinus bradycardia, isolated cells have no spontaneous pacemaker activity
Mouse	*HCN4* (HCN4)	R669Q ±	Homs embryonic lethal, but hets survive and develop SA exit block during exercise
Mouse	*HCN4* (HCN4)	573X inducible cardiac-specif- ic overexpression	Bradycardia, but no dysrhythmia
Mouse	*HCN4* (HCN4)	Inducible cardiac-specific KO	Bradycardia, reduced iso response, AV block, sudden death
Mouse	*HCN4* (HCN4)	Inducible HCN4 + cell ablation	Nodal tissue fibrosis, bradycardia, exit block, SVT, VT, complete block, sudden death
Atrioventricular block (AV block)			
Mouse	*NKX2-5* (NKX2.5)	I183P cardiac-specific overex- pression	PR prolongation, worsening AV block with age
Mouse	*NKX2-5* (NKX2.5)	R52G ±	PR prolongation, AV node smaller, AV block
Mouse	*DMPK* (DMPK)	− / − and ±	PR prolongation as mice age, ± mice develop 1st degree block, − / − mice develop third-degree block
Mouse	*GJA5* (Cx40)	− / −	Conduction delay (first-degree block)
Mouse	*TRPM4* (TRPM4)	KO	AV block (prolonged PR and QRS widening), Wenckebach
Preexcitation syndrome			
Mouse	*PRKAG2* (AMPK γ2)	R302Q cardiac-specific over- expression	Hypertrophy, preexcitation, accessory pathway, QRS widening, and inducible reentrant arrhythmia
Mouse	*PRKAG2* (AMPK γ2)	N488I overexpression	Hypertrophy, sinus bradycardia, accessory pathway, preexcitation
Mouse	*PRKAG2* (AMPK γ2)	R531G cardiac-specific over- expression	Hypertrophy, impaired contractile function, and electrical conduction abnormalities
Mouse	*TBX2* (TBX2)	Cardiac-specific KO	Accessory pathway, preexcitation
Other cardiac conduction disorders			
Mouse	*GJA1* (Cx43)	D378stop cardiac-specific inducible	Conducting truncation; germline deletion die right after birth; induc- ible model die 16 days after tamoxifen; 2–threefold QRS widening, BBB, spontaneous MVT/PVT/VF, sudden cardiac death
Mouse	*MAP2K4* (MKK4)	Cardiac-specific KO	Reduced Cx43 expression; ~ 55% QRS widening, long QTc, VT with PES
Zebrafish	*KCNJ3* (Kir3.1)	N83H cardiac-specific overex- pression	Atrial dilation; sinus arrest, sinus bradycardia, SA block, and AV block; patient mutations associated with AF
Mouse	*CACNA1D/G* (Cav1.3, Cav3.1)	CACNA1D KO or CACNA1D/CACNA1G DKO	Sinus bradycardia, slow conduction; DKO also had 3rd degree block, escape rhythms, spontaneous VT
Mouse	*KCNN3* (KCa2.3)	WT overexpression	Bradyarrhythmias, AV block, abnormal AV node, sudden death
Mouse	*IRX3* (IRX-1)	KO	His-Purkinje transcription factor; normal PR, wide QRS, notched R wave, block; spontaneous PVCs and VT; iso- and exercise-induced VT
Developmental			
Mouse	*TBX3* (TBX3)	KO	Ectopic atrial pacemakers
Mouse	*TBX5* (TBX5)	±	Hypoplasia, arrhythmias, see above in atrial fibrillation
Mouse	*MECP2* (MeCp2)	KO	X-linked; long QTc, QRS widening, pacing-inducible VT, asystole/sudden death; neuronal KO was similar

Models are separated by disease, listing the animal species, orthologous human gene and protein, mutation, notable arrhythmia phenotypes/findings, and reference. AF indicates atrial fibrillation; APD, action potential duration; AV, atrioventricular; caff, caffeine; DAD, delayed afterdepolarization; DKO, double knockout; DN, dominant negative; EAD, early afterdepolarization; GoF, gain of function; HCM, hypertrophic cardiomyopathy; het, heterozygous; HF, heart failure; hom, homozygous; HR, heart rate; IR, ischemia–reperfusion; iso, isoproterenol; KO, knockout; LoF, loss of function; MVT, monomorphic ventricular tachycardia; NSVT, nonsustained ventricular tachycardia; PES, programmed electrical stimulation; PVC, premature ventricular complex; PVT, polymorphic ventricular tachycardia; RBBB, right bundle branch block; SVT, supraven- tricular tachycardia; VF, ventricular fibrillation; and WT, wild type.

### Channelopathies

Channelopathies are caused by mutations in ion channel genes or genes that regulate ion channels and generate arrhythmia risk in the structurally normal heart. However, overlap syndromes caused by mutations in channelopathy genes (eg, SCN5A) can also be associated with alterations in cardiac structure, which are discussed in Section genetic arrhythmia syndromes associated with structural heart disease. Catecholaminergic Polymorphic Ventricular Tachycardia CPVT is characterized by arrhythmogenesis evoked by elevated catecholamines during stress or exercise. CPVT is caused by gain-of-function mutations in proteins that constitute the intracellular Ca2 + release unit of the SR. These mutations result in spontaneous Ca2 + release from RYR2 SR Ca2 + release channels, raising diastolic Ca2 + and generating membrane depolarizations and delayed afterdepolarizations via Ca2 + extrusion through the electrogenic Na +—Ca2 + exchanger. Pathological mutations in RYR2 make up more than half of identified CPVT cases and are inherited in an autosomal dominant fashion. Mutations in regulatory partners of RyR2 are rarer and include calsequestrin, calmodulin, and triadin. Evidence suggests that KCNJ2 mutations may also be involved in CPVT. Mice have been the primary animal model for studying CPVT and have been valuable not only to establish arrhythmia pathophysiology but also to identify new drug therapy that proved efficacious in humans^248^. At least 1 attempt to generate a rabbit model overexpressing human RYR2-R4497C was unsuccessful, likely due to the selected promotor and size of the transgene^249^. The first animal model of CPVT generated was an RYR2- R4496C ± knock-in mouse^29^, which had exercise- and catecholamine-inducible ventricular arrhythmias. Since then, several mouse models have been designed that carry RYR2 mutations identified from patients with CPVT (see Table 1). When loss-of-function mutations are combined with gain-of-function mutations, mice are protected from CPVT^250^. Identifying the pathogenicity of specific mutations is important because RYR2 mutations are implicated in several other arrhythmia disorders, as detailed below. Interestingly, a RYR2-exon3 deletion was identified in patients with a severe form of CPVT, but the corresponding mouse model failed to reproduce the CPVT phenotype^251^. Cardiac calsequestrin (CASQ2) mutations are inherited in an autosomal recessive manner, leading to loss of function. Casq2 serves as a high-capacity Ca2 + buffer in the SR and regulates RYR2 gating. In the mouse, CASQ2 deletion causes a severe exercise- and/or catecholamine-induced arrhythmia phenotype consistent with patients lacking Casq2 expression. Observations from CASQ2 mouse models carrying mutations identified from patients show these commonly lead to loss of Casq2 expression in the heart. Recent evidence, however, suggests that Casq2 mutations could also be inherited in an autosomal dominant manner^252^. A knock-in mouse model expressing Casq2-K180R ± reported catecholamine-induced arrhythmias validating this inheritance pattern^253^. Triadin is another protein in the SR Ca2 + release unit where autosomal recessive inheritance has been reported in patients with CPVT. When triadin was knocked out in mice, they developed substantial ultrastructural changes in the junctional SR and reduced expression of proteins comprising the Ca2 + release unit, leading to catecholamine-inducible arrhythmias^254^. Calmodulin genes (CALM1/2/3) encode 3 identical proteins (CaM) and some mutations are associated with CPVT^255^. Zebrafish models have been generated to examine the pathogenesis of overexpressing CALM mutations, and they have successfully demonstrated cardiac arrhythmias (Table 2). Finally, KCNJ2 loss of function mutations, which commonly have been linked to the Anderson-Tawil Syndrome and cause reduced IK1 current (see section Long QT syndrome), have also been identified in patients with CPVT. A knock-in mouse carrying KCNJ2 − R67Q ± had significant evoked arrhythmias without any QT prolongation (Table 2).

The CASQ2 knockout (KO) mouse is an excellent model to investigate CPVT. It has a severe and highly penetrant phenotype, something not always observed with RYR2 mouse models of CPVT (Table 2). The age of onset for CPVT is only a few weeks old and mice have spontaneous arrhythmias under normal housing conditions^256^. CASQ2 KO mice were used to establish proof of principle for the efficacy of gene-therapy in CPVT^257^. In what may be the only example of its kind for an arrhythmia syndrome, the CASQ2 KO mouse was used to establish the therapeutic efficacy of an existing FDA-approved drug, flecainide, in CPVT^248^. CASQ2 KO mice were instrumental to demonstrate that in vivo, RYR2 block is the principal mechanism of flecainide’s antiarrhythmic action^258^. Since its discovery in CASQ2 KO mice, flecainide has become the standard of care for preventing arrhythmias in CPVT patients when betablockers are insufficient^43,259^. CASQ2 KO mice have also recently been used to establish a novel tissue mechanism responsible for ventricular ectopy in CPVT (Fig. 3). To determine the cellular origin of ventricular arrhythmias in CPVT, Blackwell et al.^260^, used conditional murine models with Casq2 expression only in ventricular myocardium or in the specialized conduction system, utilizing the contactin-2 promoter to drive Cre expression and control tissue-specific Casq2 expression. CPVT occurred when Casq2 was deleted in the ventricular myocardium but still expressed in the conduction system. Moreover, catecholamine challenge did not elicit any arrhythmias when Casq2 was deleted in the conduction system but still expressed in the ventricular myocardium. Additional experiments determined that the subendocardial ventricular myocardium juxtaposed to Purkinje fibers is the only cellular source for focal ventricular arrhythmias in CPVT. To understand why that was the case, in silico modeling demonstrated an intriguing phenomenon whereby subthreshold DADs in ventricular myocardium elicit full-blown APs in the conduction system to generate arrhythmias, identifying the Purkinje-myocardial junction as the tissue origin of ventricular ectopy in CPVT (Fig. 3). This discovery could shape treatment and may be critical to our understanding of arrhythmogenesis in other ventricular arrhythmia syndromes where DADs are the cellular arrhythmia mechanism.

#### Calcium release deficiency syndrome

Whereas RYR2 gain-of-function mutations have been associated with CPVT, arrhythmogenic cardiomyopathies, and AF, loss-of-function mutations in RYR2 can cause an arrhythmia syndrome recently termed Ca2 + release deficiency syndrome (CRDS)^261^. In patients with CRDS, exercise stress tests do not provoke arrhythmias. Consequently, this syndrome can escape clinical diagnosis and often presents as sudden cardiac death. In CRDS, arrhythmias are thought to develop because of electrophysiological remodeling. Given the substantial number of benign RYR2 mutations observed, predicting pathogenicity without in vitro or in vivo examination is challenging. CRDS has only been recently described, but 1 knock-in mouse model was generated carrying the patient-specific RYR2-D4646A ± mutant allele^261^. Exercise and catecholamine challenge did not invoke arrhythmias, as predicted. Importantly, this animal model enabled the authors to establish a burst pacing protocol that induced arrhythmias, supporting a possible new clinical diagnostic tool, which was recently confirmed in a clinical study. A previously reported loss-of-function mutation, RYR2-A4860G ± , was identified in a patient with idiopathic VF and subsequently knocked-in to a mouse, but arrhythmias were not reported in vivo^262^. Ex vivo hearts did develop VF in response to isoproterenol but had no arrhythmias in the presence of the RyR2 agonist caffeine. These data hint that this RYR2 loss-of-function mutation may be part of the calcium release deficiency syndrome. Prior work on RYR2 has focused on gain-offunction mutations. CRDS is a relatively new syndrome and animal models provide an opportunity to advance our understanding of CRDS pathophysiology and determine the impact of loss-of-function mutations.

### Long QT syndrome

Congenital LQTS often presents as a multiorgan syndrome caused by mutations in proteins responsible for the repolarization of the heart and can cause seizures, syncope, arrhythmia, and sudden death. Alterations in repolarization manifest as prolongation of the AP and, consequently, the QT interval, predisposing the heart to EAD, DAD, and reentrant circuits. Ca2 + , Na + , and many K + channels play a role in cardiac repolarization; accordingly, the genes involved in this syndrome vary and LQTS can be inherited in autosomal dominant or recessive forms. LQTS is traditionally described as a distinct disease manifestation; however, long QT intervals are observed in many overlap syndromes (see Table 2). Approximately 80% of LQTS cases are caused by mutations in KCNQ1 or KCNH2, with SCN5A constituting 7% to 10% of cases^263^. It should be reiterated that the currents responsible for mouse and human repolarization are quite different (Fig. 2), and mice are inadequate to model human disease involving mutations in delayed rectifier K + channels (eg, LQT1, LQT2).

An aspect of understanding LQT pathogenesis is the observed sex differences in patients, such as QT interval, the onset of cardiac events, and sudden death^264,265^. Animal models allow for significant insight into these investigations since most in vitro models fail to capture the hormonal factors that influence development and regulation. It is noteworthy that sexual dimorphism in QT is not captured by mouse models and may require other animal models such as rabbits^266,267^. Estradiol treatment in ovariectomized rabbits led to prolongation of the QT interval and changes in proteins responsible for repolarization, whereas dihydrotestosterone did not^268^. Another study in rabbits recapitulated findings in patients showing that QT interval was more prolonged in females following treatment with erythromycin^269^.

LQT1 is caused by loss-of-function mutations in KCNQ1 (Kv7.1). Knockout of KCNQ1 in mice leads to characteristics of Jervell and Lange-Nielsen syndrome, causing deafness and prolonged QT interval^270^. Other models have examined distinct mutations with varying phenotypes; however, caution should be used when reviewing the mouse LQT1 literature. Rabbits are a much better model for examining human-like mechanisms of altered repolarization. However, only 1 transgenic rabbit model is reported, which carries cardiac-specific overexpression of KCNQ1-Y315S, leading to LQT and inducible EADs and VT with sympathetic stimulation^271^.

LQT2 is caused by loss-of-function mutations in KCNH2 (Kv 11.1). In contrast to humans, KCNH2 contributes little to repolarization in mice (Fig. 2). As such, mouse models are not realistic for modeling LQT2 and should not be used. A rabbit transgenic model was generated with cardiac-specific overexpression of KCNH2-G628S and developed LQT, spontaneous PVT, and sudden death. Arguably, the most interesting LQT2 model is the work in zebrafish^272^. In 1 study, the endogenous ortholog of hERG (zERG) was knocked down using morpholinos and then various hERG mutants were expressed in its place. The phenotype data (prolonged APD and/or 2:1 AV block) correctly identified 39/39 pathogenic mutants and 9/10 nonpathogenic polymorphisms. Further work using this model could establish the pathogenicity of other variants of uncertain significance.

LQT3 is caused by gain-of-function mutations in SCN5A (Nav1.5). Several mouse models have been generated that recapitulate the LQT phenotype. Both the SCN5A-ΔKPQ/ + and SCN5A-ΔQKP/ + mouse models report many characteristic phenotypes such as prolonged QT interval, a more pronounced T wave, prolonged APD, arrhythmias, and sudden death, which make them suitable for examining LQT3 mechanisms and pathogenesis^273,274^. A successful bench-to-bedside study, conducted in ventricular myocytes isolated from guinea pigs, demonstrated that mexiletine restored the APD in cells treated with the Na + channel inactivation inhibitor, anthopleurin^275^. Mexiletine is now used routinely in the treatment of patients with LQT3. In rare cases, patients have been identified linking LQTS with mutations in the beta accessory proteins for SCN5A. An SCN1B knockout mouse was generated and had long QT, bradycardia, and delayed repolarization; interestingly, it was found that these mice had increased Na + channel expression. Nav1.5 channel gating was unaffected, but peak and persistent currents were increased in isolated cardiomyocytes.

Andersen-Tawil syndrome is a rare LQTS associated with physical abnormalities and hypokalemic periodic paralysis and is primarily caused by loss of function mutations in KCNJ2 (Kir2.1), resulting in reduced IK1 current. Neonatal (1 day old) KCNJ2 knockout mice were characterized by long QT and bradycardia, before dying from complete cleft palate and inability to feed^276^. A more useful arrhythmia phenotype-LQT and spontaneous VT—was observed in the KCNJ2-T75R cardiacspecific overexpression mouse model^277^. The remaining LQTS types result from mutations in K + channels, Ca2 + channels, and key regulators of ion channel function (Table 2). The ankyrin-B syndrome is characterized as an overlap syndrome, with long QT, sinus node dysfunction, conduction abnormalities, exerciseinduced arrhythmia, VF, and VT. The ankyrin-B knockout mouse demonstrated long QT and sinus node dysfunction^278^; however, a more severe and faithful phenotype was reported in heterozygous mice, capturing many of the same observations from humans^279^. KCNE1 knockout mice developed long QT, but only when heart rate deceleration occurred^280^. A transgenic rabbit model overexpressing dominant negative KCNE1-G52R developed long QT and increased susceptibility to drug-induced arrhythmia by accelerating IKs and IKr deactivation kinetics^281^. This model could be useful for examining the proarrhythmic liability of drugs. CACNA1C (Cav 1.2) gainof-function mutations cause LQTS that can be associated with extracardiac manifestations known as Timothy syndrome. In mice, CACNA1C knockout is embryonic lethal, but heterozygous mice survived without any cardiac phenotypes. Cardiac-specific overexpression of the G406R mutation in mice led to long QTc and exerciseinduced PVCs and Torsades de Pointes (TdP)^282^. It was hypothesized that this mutant had altered interaction with AKAP; in fact, when G406R-overexpressing mice were crossed with AKAP150 KO mice, they were protected against all phenotypes of the G406R mutant.

Investigators have also knocked out many of the potassium channel genes in mice to determine their effects on cardiac electrophysiology. When a dominantnegative fragment of KCNA1 was expressed, mice developed LQT and spontaneous ventricular arrhythmias^283^. Interestingly, it was later shown by Glasscock et al.^284^ that KCNA1 is preferentially expressed (≈tenfold higher) in atria over ventricles. Programmed electrical stimulation (PES) induced AF in KCNA1 knockout mice but did not lead to any ventricular arrhythmias and no differences in QT interval were observed. Dominant negative overexpression of KCNA5, KCNB1, or KCND2 all prolonged the QT interval without any other overt electrophysiological changes. Mice do not express KCNE3 in the adult heart, but deletion led to long QTc in aged female mice because of hyperaldosteronism^285^.

Limitations-Comments:

Mouse models of LQTS should be viewed with caution when the repolarizing current of interest does not reflect the human AP. As transgenic rabbit models become more common, they may find strong ground in advancing our understanding LQT pathogenesis. Rabbits accurately capture sex differences and express a similar repolarization ion channel gene profile as humans. An area of value will be evaluating predisposition to drug-induced LQT and arrhythmia^286^, which rabbit models will be best suited to answer.

### Short QT syndrome

Short QT syndrome (SQTS) is an extremely rare disorder; only a few 100 cases have been identified to date. SQTS is caused by the shortening of the cardiac AP. Like long QT syndrome, alterations in cardiac repolarization alter the QT interval. Because of the abbreviated QT interval, the refractory period is also shortened, leaving the heart susceptible to reentrant arrhythmias. Symptoms associated with short QT syndrome include both atrial and ventricular fibrillation, palpitations, and sudden cardiac death. Gain-of-function mutations in KCNH2, KCNQ1, and KCNJ2 or loss-of-function mutations in CACNA1C, CACNB2, CACNA2D1, SCN5A, and SLC4A3 have all been associated with SQTS.

There are few genetic animal models available to examine SQTS in vivo. Zebrafish carrying the KCNH2- L499P mutation have a shortened QT interval^287^. Knockdown of SLC4A3 in zebrafish led to a short QT interval that was rescued by expressing WT SLC4A3 but not by SLC4A3-R370H, identified from a patient with SQTS^288^. To appreciably capture the human cardiac AP, a rabbit model was engineered to overexpress KCNH2 carrying the N588K mutation^289^. Transgenic rabbits had shortened AP and QTc but a normal T wave height. Ex vivo perfused hearts had inducible VT and VF; this model is arguably the best available and, as discussed above, rabbit models more accurately typify human cardiac repolarization.

### Brugada syndrome

Brugada syndrome (BrS) is a disorder characterized by elevated ST segment, partial bundle branch block, arrhythmia, and sudden cardiac death. It is commonly caused by mutations in SCN5A; however, ≈20 genes are now associated with BrS. Brugada ECG patterns are sometimes observed in overlap syndromes, such as with long QT syndrome, as SCN5A mutations are associated with several arrhythmia disorders. In mice, SCN5A deletion is embryonic lethal, but heterozygous mice survived and developed slowed conduction, pacing-induced VT, and fibrosis with age^290^. Interestingly, the authors observed variability in phenotype penetrance that correlated with NaV1.5 expression levels^291^. A mouse model for an overlap syndrome of LQT and Brugada was generated to carry 1795insD in SCN5A^292^. Homozygous mice were embryonic lethal, but heterozygous mice developed sinus node dysfunction, slowed conduction, bradycardia, and QT prolongation. Interestingly, CaMKII-dependent phosphorylation of wild-type Nav1.5 appears to phenocopy this mouse model, as late current predominates at slower heart rates. Phosphomimetic and phosphoablation mouse models demonstrated that phosphorylation and oxidation modulate Nav1.5 current and susceptibility to arrhythmias^293,294^. A transgenic pig model was generated to better understand BrS disease mechanisms. Pigs were designed to carry the orthologous SCN5AE558X/ + mutation identified in a patient diagnosed with BrS and developed conduction abnormalities and QRS widening but had no elevated ST segment, arrhythmias, or sudden death through 2 years of age^295^. However, ex vivo hearts had increased susceptibility to VF with programmed stimulation. One debate surrounding BrS is whether many of the associated genes cause BrS or simply increase susceptibility to developing BrS. SCN5A mutations have variable and incomplete penetrance and differing phenotypes. Moreover, SCN5A mutations are prevalent in the general population and discerning pathogenesis can be difficult. Given the failure of animal BrS models to reproduce the full clinical syndrome, their utility studying BrS pathogenesis and treatment options remains to be determined.

## Genetic arrhythmia syndromes associated with structural heart disease

Animal models have been beneficial for understanding the pathogenesis of arrhythmias caused by mutations in non-ion channel genes that result in structural heart disease. The section below discusses the major arrhythmia syndromes associated with a cardiomyopathy phenotype (ARVC, HCM, and DCM) and microscopic structural disease such as AF, sick sinus syndrome, heart block, and preexcitation syndromes.

### Arrhythmogenic right ventricular cardiomyopathy/dysplasia

ARVC/dysplasia is a disease manifesting as fibro-fatty replacement of the right ventricular myocardium and widespread electrophysiological remodeling, predisposing individuals to ventricular arrhythmias and increased risk of sudden death. Approximately half of the ARVC cases are caused by mutations in desmosomal proteins, which make up cell–cell mechanical junctions. Arrhythmias often arise during periods of exercise or stress, suggesting that catecholamines contribute to arrhythmogenesis. Accordingly, one of the primary phenotypes in animal models is catecholamineinduced ventricular arrhythmias.

The primary genetic model used to study ARVC has been the mouse. Of note, mice do not get fatty infiltration in the heart, one of the primary phenotypes of ARVC in humans. However, numerous cardiac phenotypes have been found, from remodeling to arrhythmogenesis. The first genetic mouse models to examine the importance of desmosomal proteins were congenital knockouts (Table 2). Mouse models with global knockout of plakoglobin, desmoplakin, plakophilin-2, and desmoglein-2 are all embryonic lethal. However, heterozygotes survived and developed varying degrees of fibrosis and arrhythmia phenotypes (Table 2). Subsequent animal models were based on patient mutations and frequently resulted in haploinsufficiency. This seems to be the primary cause, along with repression of the Wnt signaling pathway. DSC2 (desmocollin-2) mutations are rare, and the link between pathogenesis and this protein is not well understood because data are lacking and conflicting. DSC2 knockout mice are viable but did not develop any cardiac phenotypes. However, DSC2 knockdown reportedly led to altered ultrastructure and contractile dysfunction, while overexpression also led to fibrotic remodeling^296^. Germline knockout of DSG2 (desmoglein-2) results in embryonic lethality in mice, but the cardiac-specific knockout led to dilation, fibrosis, and electrophysiological remodeling. Finally, the inhibitor of apoptosis-stimulating protein of p53 has been linked to ARVC; knockout mouse model developed dilation, arrhythmia, and sudden death^297^.

The PKP2 (plakophilin-2) mouse model is well-suited for understanding ARVC mechanisms and disease progression. Heterozygote PKP2 mice survived and developed arrhythmias in the absence of overt structural remodeling^296^, which was validated in mice carrying a PKP2 truncation mutant^298^. These findings raised an interesting question about the cause of arrhythmias: how do arrhythmias arise in the absence of structural changes to the heart? Because PKP2 knockout is embryonic lethal, a cardiac-specific inducible PKP2 knockout mouse was generated, which had many of the characteristic ARVC phenotypes: fibrosis, remodeling, reduced ejection fraction, arrhythmia, and sudden death^299^. In this model, PKP2 signaling regulates transcription of many genes involved in Ca2 + homeostasis and proteins of the intracellular Ca2 + release unit are downregulated, causing proarrhythmic RyR2 activity. The authors discovered that flecainide, a class Ic antiarrhythmic that inhibits RyR2 channels, effectively prevented arrhythmias in these mice. Exercise exacerbated RyR2 hyperactivity, and it was shown that membrane-permeable beta-blockers had greater efficacy than nonpermeable beta-blockers^300^.

ARVC has been observed in dogs and cats, generally of unknown cause. In these models, fibro-fatty replacement is commonly observed alongside syncope, arrhythmias, and sudden death. Dog models could be considered when disease mechanisms, as they relate to human cardiac (electro)physiology, are essential. Nondesmosomal proteins, such as phospholamban, RyR2, lamin A/C, transmembrane protein 43, and integrin linked kinase have all been associated with ARVC in patients; however, their role is less well understood due to the spectrum of phenotypes in ARVC. For example, some mutations in phospholamban cause DCM, which is a differential diagnosis, but may also present with ARVC. Because of the difficulty of identifying pathogenic mutations, diagnostic criteria rely on interpreting cardiac imaging and ECG data. An important line of investigation understands the molecular mechanisms that drive the development of the disease, as early disease progression escapes detection and the heterogeneity of genes involved makes prognosticating a diagnosis difficult.

### Dilated cardiomyopathy

Congenital DCM is characterized by ventricular dilation and is commonly caused by mutations in cytoskeletal or myofibrillar proteins leading to remodeling, reduced ejection fraction, conduction abnormalities, arrhythmia, and sudden death. Many animal models have been created to investigate the structural and functional consequences of these mutations. Here we focus on animal models where arrhythmias are a prominent feature of the reported phenotype (Table 2). Of note, there is significant overlap with the clinical diagnosis of arrhythmogenic (right ventricular) cardiomyopathy.

A widely studied DCM mutant is SCN5A-D1275N. A wide spectrum of phenotypes in different families carrying this mutation have been reported, including AF, conduction defects, and sinus dysrhythmia^301,302^. It is speculated that the variation in genetic background between the families contributes to these phenotypes. Homozygous SCN5A-D1275N mice developed many of the characteristic arrhythmia phenotypes observed in humans, while heterozygous mice did not^303^. Many changes in the ECG parameters reflected a gene dose-dependent effect and these features were also observed in zebrafish^304^.

Mutations in LMNA cause severe DCM and sudden death. Several mouse models have been generated that carry mutations identified from patients (Table 2). A variety of symptoms have been reported and spontaneous arrhythmias occur frequently. Mutations in RMB20 also have a severe phenotype in mice, commonly leading to spontaneous ventricular arrhythmias and sudden death. Duchenne muscular dystrophy has been studied in pig, dog, and mouse models, each having electrophysiological changes (Table 2).

### Hypertrophic cardiomyopathy

HCM is characterized by enlargement of the left ventricle primarily caused by mutations in sarcomeric proteins, leading to increased risk of arrhythmia and sudden death^305^. HCM is commonly caused by mutations in either the beta myosin heavy chain or myosin binding protein C, together accounting for nearly half of all cases. Mice express beta myosin heavy chain during cardiogenesis, but rapidly switch from the beta to the alpha isoform, postnatal. Numerous HCM animal models have been generated; however, the predominant focus has been the study of underlying changes in sarcomere function, contractility, and hypertrophy, without detailed examination of electrophysiological phenotypes. Many studies have reported on arrhythmia susceptibility in ex vivo hearts but only reports examining in vivo arrhythmia phenotypes are discussed here.

Alpha myosin heavy chain mutations are commonly associated with HCM, but the only reports of arrhythmias in an animal model come from the R403Q mutant, which was identified in MYH7 from a human patient. The knock-in mouse model was designed to carry R403Q in MYH6^306^. Mice developed HCM but only had a modest arrhythmia phenotype. A rabbit model was generated to overexpress the transgene but did not exhibit an arrhythmia phenotype^307^. Troponin mutations are also associated with HCM and a transgenic mouse was generated to carry TNNI3-G203S^308^. Mice had conduction defects, but no other arrhythmias. However, when these mice were crossed with MYH6-R403Q mice, they developed a more severe phenotype with conduction defects, LQT, and catecholamine-induced VT. Knockout of MYBPC caused long QT and spontaneous VT in mice^309^.

Several troponin T models have been designed to carry mutations identified in patients. Knollmann et al.^310^ showed that TNNT2-I79N mice had tachycardia, isoproterenol-inducible ectopy, and spontaneous nonsustained VT, despite having no hypertrophy or fibrosis. Subsequent work in mice demonstrated that Ca2 +—sensitizing TNNT2 mutations cause inducible arrhythmias, whereas nonsensitizing mutants (R278C) do not leave the heart susceptible to arrhythmias^311^. Myofilament Ca2 + sensitization increases cytosolic Ca2 + -binding affinity, alters int racellular Ca2 + homeostasis, and causes pause-dependent Ca2 + -triggered arrhythmia. In addition, myofilament Ca2 + sensitization causes focal energy deprivation, which further increases arrhythmia susceptibility in mice^312^. Hence, myofilament sensitization per se, caused by drugs, mutations, or posttranslational modifications after myocardial infarction^313^, is a novel arrhythmia mechanism^314^. These reports illustrate the power of murine HCM models for discovering new arrhythmia mechanisms and identifying therapeutic targets.

### Atrial fibrillation

AF is the most common arrhythmia and is characterized by rapid, abnormal atrial rhythms, with symptoms manifesting as palpitations, syncope, stroke, and heart failure, among others. The etiology of AF is multifactorial, stemming from environmental factors, diet, lifestyle, family history, medication, and surgery. For an in-depth review of acquired AF and various animal models available, the reader is referred to this review^36^. Many genetic animal models develop AF alongside their primary disease phenotype (see Tables 2 and 3), but the models discussed in this section are more directly related to AF as a primary pathology.

One trigger for AF is thought to be hypersensitive and leaky RyR2 channels. Thus, animal models of CPVT (both gain-of-function RyR2 and loss-of-function Casq2) have been used to investigate arrhythmogenic mechanisms and screen therapeutic modalities^315^. It was demonstrated that RyR2 inhibition can attenuate AF in these models. Other CPVT models are susceptible to AF with PES. A more severe phenotype was seen with CREM mice, which developed atrial dilation and spontaneous paroxysmal and persistent AF that worsened with age^316^. A second trigger for AF may be channelopathies that accentuate excitability. KCNE1, SCN5A, KCNQ1, SK2, and SK3 mutations have all been introduced into mice, with varying phenotypes (Table 2). The KCNE1 knockout mouse develops spontaneous AF^317^. SCN5A models show overt structural changes, fibrosis, conduction abnormalities, and mitochondrial injury. Proteins associated with development, signaling pathways, and transcription regulation have been found to induce AF. Atrial-specific or complete knockout of LKB1 in mice caused electrical and structural remodeling and mice developed spontaneous AF^318,319^. A goat model overexpressing constitutively active TGF-β1 had atrial fibrosis and AF inducibility with PES^320^. Genome-wide association studies have identified many gene loci associated with increased AF risk. AF loci include genes known to affect ion channel function, cardiogenesis, or cell–cell conduction, although in many cases, candidate genes have not been determined. There are several animal models of inherited AF, mostly in mice. A primary challenge with mouse models of AF is that they do not commonly develop spontaneous AF, instead, only uncovering the phenotype with PES. Moreover, AF typically lasts for a brief period (on the order of seconds) before resolving. However, some mouse models have more severe and protracted AF that occurs spontaneously (Table 2). AF manifests in a wide range of cardiac diseases and resolving causative versus correlative pathogenesis is ongoing for some genetic models. Despite these shortcomings, investigators have successfully captured AF phenotypes in many different mouse models for genes identified in genomewide association studies studies or laboratory testing.

Genome-wide association studies have identified loss of function (PITX2, TBX5, GJA1) and gain of function (KCNN3) variants associated with patients with AF. PITX2 heterozygous mice had normal cardiac parameters except reduced transpulmonary flow; however, ex vivo hearts were more susceptible to atrial pacing-induced arrhythmia^321^. Subsequent work showed AF in vivo with PES and several groups have used this model to study AF and explore treatment^322^, especially since PITX2 is the most common risk locus identified in patients with AF. Inducible deletion of the TBX5 transcription factor led to a much more severe phenotype of spontaneous AF and electrophysiological remodeling within 2 weeks of tamoxifen treatment^323^. Gene profiling identified numerous changes in the expression of Ca2 + handling proteins and ion channels, including PITX2. These data provide a link between TBX5-PITX2 activity and electrophysiological protein regulation in the heart. AF may also be promoted by delayed conduction. Mice carrying the GJA1-G60S/ + mutation are more susceptible to pacing-induced AF^324^. Mice overexpressing KCNN3 had bradyarrhythmias, heart block, abnormal AV node morphology, and sudden death^325^.

Many other models have explored mutations or deletion of other ion channels, transcription factors, developmental pathways, or hormones to examine AF (Table 2). Data from patients have frequently confirmed the downregulation of various proteins, providing yet another link to pathogenesis. AF is a complex multifactorial disease; each animal model is an opportunity to extend our understanding of this disease.

### Sick sinus syndrome

Sick sinus syndrome occurs when the sinoatrial node is not capable of generating a normal rhythm. In heterozygous SCN5A ± mice (see also Brugada Syndrome), investigators identified sick sinus syndrome phenotypes and sex differences in sinoatrial node function with age^326^. The hyperpolarization-activated cyclic nucleotide-gated channels (HCN) play an essential role in generating spontaneous pacemaker activity. Various models have examined the deletion of HCN1-4. HCN4 is the most abundant isoform, and congenital deletion in mice is embryonic lethal. However, embryonic hearts had sinus bradycardia^327^. Inducible cardiac-specific knockout led to severe bradycardia, block, and sudden death within days^328^. Mice with germline deletion of HCN1 or HCN2 survived and developed sinus dysrhythmia^329,330^. Deletion of HCN3 did not lead to bradycardia or arrhythmia, but mice had subtle perturbations in their ECG morphology at lower heart rates. Perhaps the most elegant and accurate mouse model of sick sinus syndrome is an inducible HCN4-specific cellular ablation mouse^331^. This model accurately captured many of the phenotypes observed in patients, such as fibrosis of nodal tissue, sinoatrial dysrhythmia, supraventricular tachycardia, VT, and sudden death.

### Atrioventricular block

AV block is partial or complete disruption of impulse propagation from the atria to the ventricles. As the single site for collating atrial depolarization and passing it to the ventricle, aberrant AV node function may prevent or delay sinus rhythm from conducting into the ventricle. The AV node makes up part of the conduction system and, as such, expresses many of the same ion channels and gap junctions as the sinoatrial node, bundle of His, and Purkinje cells. AV block is observed in many of the models described above. However, some genes seem to specifically alter AV node conduction and effect block without altering sinus node rhythm. The NKX2-5 gene appears to be definitely related to AV block in patients, and mouse models carrying 2 different mutations independently validated the role of this gene in disease progression^332,333^.

### Preexcitation syndrome

Preexcitation occurs when the ventricles are activated prematurely via an accessory pathway. The most common mouse models generated to study preexcitation syndrome have mutations in PRKAG2, a regulatory subunit of 5′ AMP-activated protein kinase^334–336^. Mutations in the TBX2 transcription factor also led to the development of an accessory pathway in mice^337^.

## Arrhythmia mechanisms in human induced pluripotent stem cell–derived cardiomyocytes

**Figure 11:**
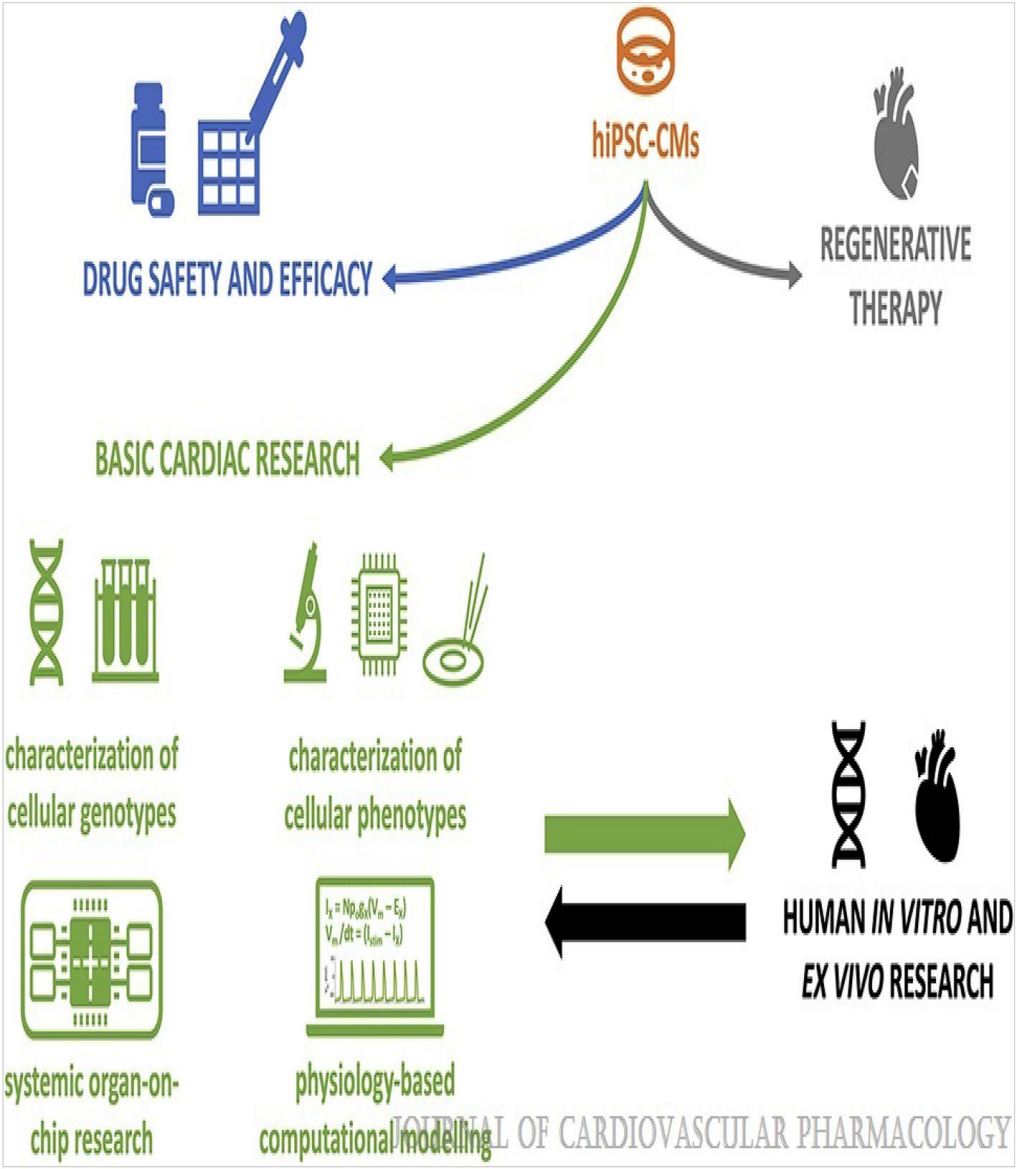
Scope of the review. Although arrhythmogenic properties of hiPSC-CMs are very relevant in all 3 principal application fields (basic research, drug screening, and regenerative therapy), we focus on the first one and on the second one to a lesser extent. Also, we link the hiPSC-based findings to the human/patient context whenever possible. (Paci, Michelangelo; Penttinen, Kirsi; Pekkanen-Mattila, Mari; Koivumäki, Jussi T. Arrhythmia Mechanisms in Human Induced Pluripotent Stem Cell–Derived Cardiomyocytes. Journal of Cardiovascular Pharmacology77(3):300–316, March 2021. 10.1097/FJC.0000000000000972).

The advent of human induced pluripotent stem cell– derived cardiomyocytes (hiPSC-CMs) in 2007 has solved one fundamental challenge in basic cardiac research and drug discovery; now, there is an unlimited source of CMs of human origin, both from healthy individuals and patients with cardiac diseases^338^. The hiPSC-CM discovery opened a plethora of different use cases as follows: tests on drug cardiotoxicity and efficacy, understanding arrhythmic mechanisms, modeling diseases in a dish, developing patient-specific models and personalized drugs, and regenerative medicine. Some use cases are in a more developed state than others (Fig. 11). For example, using hiPSC-CMs for drug cardiac safety assessment was theorized in 2013 within the Comprehensive in vitro Proarrhythmia Assay (CiPA)^339^, as a step to confirm the results of in vitro and in silico tests on drug effects on multiple cardiac ion channels and the cardiac action potential (AP). At the time of this review, the CiPA initiative has already obtained the support of regulatory agencies and pharmaceutical companies, and it is at a very advanced state (cipaproject.org/timelines/). Conversely, other applications are still in their infancy, for example, the development of personalized therapies informed by in-depth analysis of patient-specific hiPSC-CMs and personalized medicine^340^. Furthermore, the full potential of hiPSC-based applications remains to be harnessed because these cells do not adequately recapitulate (1) morphological and ultrastructural, (2) electrophysiological, (3) contractile, and (4) metabolic properties of native adult human CMs. Efforts aimed at solving these challenges have been recently reviewed by others^341–345^. Here, we review the advances that the hiPSC-based modeling systems have brought so far regarding the understanding of both arrhythmogenic triggers and substrates. First, we assess how different mechanisms of arrhythmogenesis that relate to abnormal impulse formation and propagation have been captured. Then, we explore to what extent can hiPSCbased approaches recapitulate the different atrial and ventricular arrhythmogenic phenotypes. The usability of hiPSC-CMs in investigating drug-induced arrhythmias and sex-dependent arrhythmic characteristics are discussed in the following chapters. Finally, we provide some conclusions and speculate about future directions.

**Figure 12:**
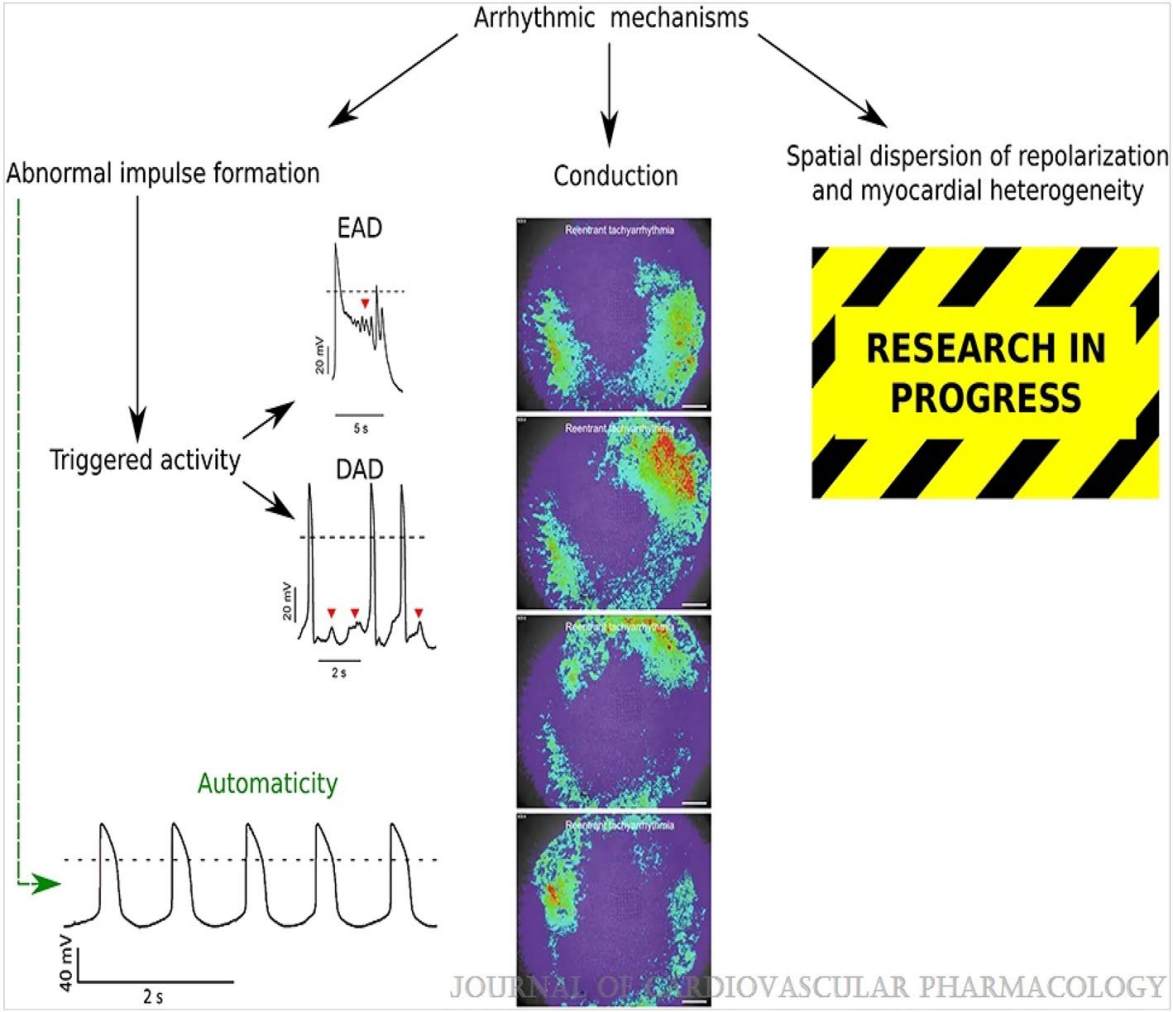
The classification of arrhythmic mechanisms used in this review article. The panels for EADs and DADs were adapted from Ref. 43 under CC BY 3.0 (original Figs. 5A and 7G, http://creativecommons.org/licenses/by/3.0/). The panel for automaticity was adapted from Ref. 142 under CC BY 3.0 (original Fig. 2A). The panel for conduction was adapted from Ref. 77 under CC BY 4.0 (original Supplementary Movie 5, http://creativecommons.org/licenses/by/4.0/). (Paci, Michelangelo; Penttinen, Kirsi; Pekkanen-Mattila, Mari; Koivumäki, Jussi T. Arrhythmia Mechanisms in Human Induced Pluripotent Stem Cell–Derived Cardiomyocytes. Journal of Cardiovascular Pharmacology 77(3):300–316, March 2021. 10.1097/FJC.0000000000000972).

**Figure 13:**
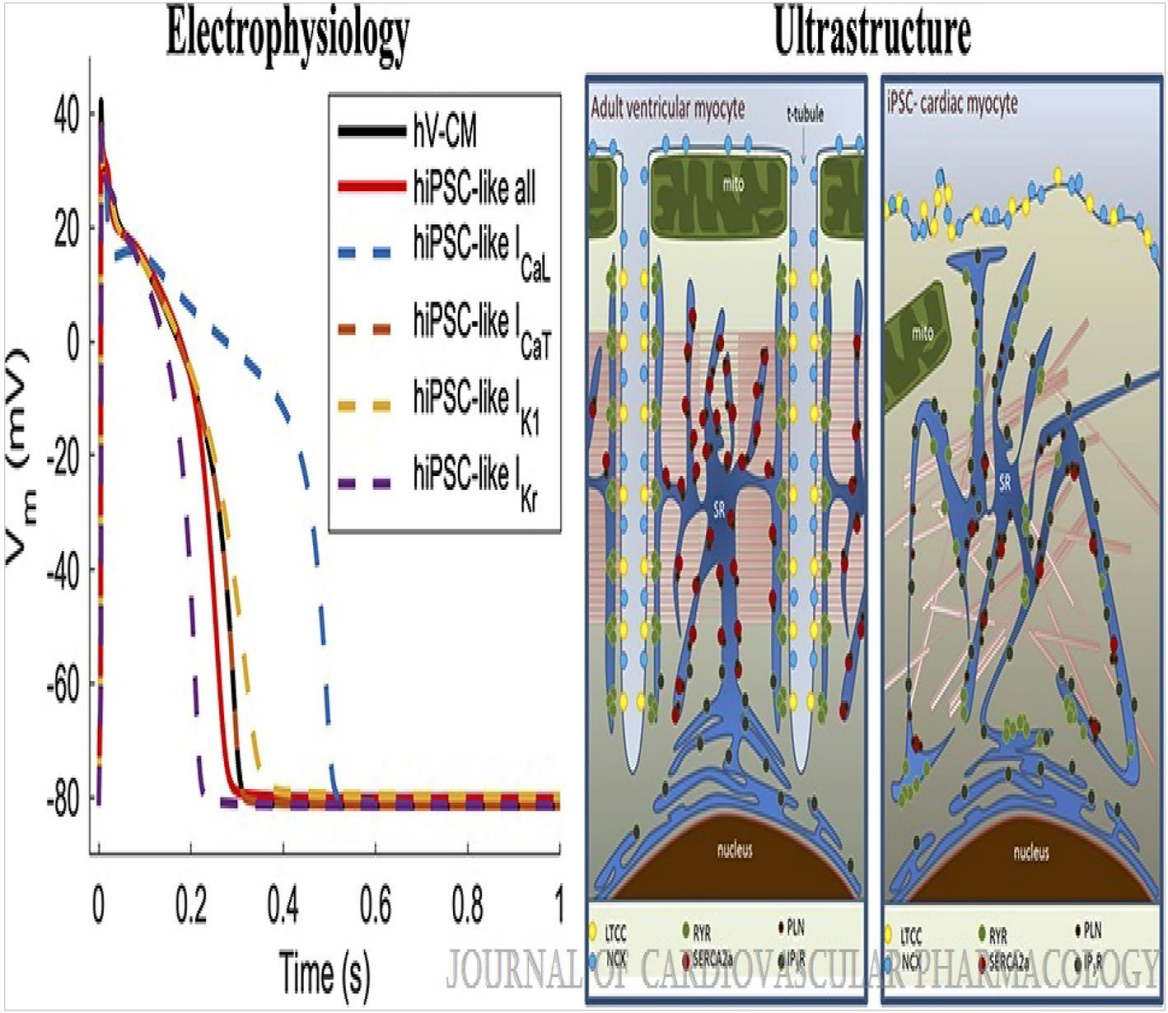
Distinct electrophysiological and ultrastructural characteristics of hiPSC-CMs in comparison with human native/adult ventricular CMs (hV-CMs). Impact of prototypical hiPSC-like ICaL, IK1, and IKr densities, which are 1.5-fold, 0.5-fold, and fourfold in comparison with hV-CM, on the repolarization reserve, simulated as in Ref. 193. The absence of t-tubules in hiPSC-CM results in spatial uncoupling of L-type Ca2 + channel (LTCC) and ryanodine receptors (RyR), as well as irregular distribution of SR Ca2 + handling proteins: RyR, Ca2 + ATPase (SERCA), and its regulatory protein phospholamban (PLN). Furthermore, inositol triphosphate receptors (IP3R) activity is substantially higher in hiPSC-CMs. The panels for were adapted from Ref. 196 CC BY 4.0 (original Fig. 1, http://creativecommons.org/licenses/by/4.0/). (Paci, Michelangelo; Penttinen, Kirsi; Pekkanen-Mattila, Mari; Koivumäki, Jussi T. Arrhythmia Mechanisms in Human Induced Pluripotent Stem Cell–Derived Cardiomyocytes. Journal of Cardiovascular Pharmacology77(3):300–316, March 2021. 10.1097/FJC.0000000000000972).

One of the aforementioned benefits of hiPSC-CMs is the availability of a pool of in vitro human cell models to reproduce the occurrence of arrhythmias under different conditions, and to understand better the underlying mechanisms^346^, as well as developing pharmacological strategies to reduce them. Examples of use cases are the presence of mutations altering specific membrane channels (channelopathies)^347–353^, or other cell functions^354–358^, or when administering drugs^359^. For all these use cases, the clear advantage of hiPSC-CMs is that they are from human source. A potential disadvantage is that they are relatively recent in vitro models, and a better understanding of the actual similarity to native human CMs in terms of arrhythmic event development is needed^360^. A well-known scheme to classify mechanisms of cardiac arrhythmias^6,361^ identifies 2 macrofamilies, separating nonreentrant and reentrant activities: abnormal impulse formation and improper impulse conduction. In this review, we follow this scheme (Fig. 12), reporting abnormal impulse formation and improper impulse conduction for hiPSC-CMs and expand the mechanisms adding spatial dispersion of repolarization and myocardial heterogeneity (Figs. 11, 12, 13).

### Limitations of hiPSC-CMs

Despite the undeniable value of hiPSC-CMs as a human in vitro model, it is fair to recognize that they suffer specific limitations as models fully representative of hV-CMs, as we reported in the previous sections of this review. In this section, we recapitulate and expand on those limitations (Figs. 11, 12, 13).

Some of the ion currents are differently expressed in hiPSC-CMs and hV-CMs. hiPSC-CMs express 2 ion channels that are absent in healthy hV-CMs: If^362^ and ICaT^363^. Furthermore, the balance of repolarizing potassium currents is quite different. The expression of IK1 has been reported as low as in human adult atrial CMs (hA-CMs)^364^ or even absent^360^. Whereas, the density of IKr is substantially higher in hiPSC-CMs compared with hV-CMs^352,362,365^. The balance of IK1 and If underlies hiPSC-CM automaticity in the voltage clock paradigm; one of the hallmarks of the immature or non-adult hiPSC-CMs. In addition, expression of some ion channels and consequent ion current densities seems to be highly variable in comparison with hV-CMs: lower^366^ or similar^362^ SCN5A and lower^366^ or similar^362^ KCNQ1. A huge range of ICaL densities has been reported for hiPSC-CM: 3.3–17.1 pA/pF^367,368^, which is likely because of different methodology. The only study comparing ICaL under identical experimental conditions showed 50% larger densities in hiPSC-CMs versus hV-CMs^363^. Together, the aberrant balance of inward and outward ion currents reduces the repolarization reserve in hiPSC-CMs compared with their native counterparts. For example, Blinova et al.^366^ correlate these differences in expressions to the different proarrhythmic effects that a set of drugs (namely, bepridil, ranolazine, and mexiletine) has in hiPSC-CMs. Furthermore, Lemoine et al^362^ showed that EHT is much more prone to EADs in comparison with human ventricular tissue.

Ca2 + handling is functional but not fully developed in CMs derived from pluripotent stem cells^369^. Two main points are worth to be mentioned. First, the lack or lower expressions of functional proteins, eg, junctin and triadin (to facilitate RyR function), and of calsequestrin for Ca2 + bufferinas well as lower SERCA and RyR. Second, hiPSC-CMs lack a well-developed T-tubule network that is a hallmark of mature ventricular CMs^370^. This ultrastructural immaturity results in an U-shaped Ca2 + wavefront with a remarkable delay in peak between the cell periphery and center^371^, as observed also in hA-CMs, and a poor excitation–contraction coupling^372^.

The hiPSC-CM contraction is also nonoptimal, not only because of poor excitation–contraction coupling, but also to the immature sarcomeric structure and the orientation in multiple directions of the myofibers within the cell^342^. When compared with native human ventricular tissue, hiPSC-based EHTs lack a positive force–frequency relation^371^, which is one of the hallmarks of cardiac contractility. Furthermore, the frequency-dependent acceleration of relaxation is much weaker in EHTs^373^.

Finally, the hiPSC-CM potential to recapitulate the contractile and remodeling signaling of adult CMs is not fully known. Jung et al.^374^ studied the different stages of hiPSCCMs and showed that at day 30 b2-AR signaling is dominant, reflecting a relative immaturity of hiPSC-CMs. By day 60, a transition to downstream signaling by the b1-AR represents a more “adult-like” phenotype. Even by day 90, b1-AR expression does not fully recapitulate the pattern seen in adult human ventricles, and b-ARs only activate CaMKII at this time, although the protein expression reached a maximal level by day 30. The differential maturation of intracellular signaling pathways in hiPSC-CMs should be considered in disease modeling and drug testing.

## Emerging etiologies of arrhythmogenesis: opportunity for animal models

Cardiac electrophysiology is regulated not only by the amino acid sequence at the protein level but also by posttranslational modifications, micropeptides, epigenetics, long noncoding RNAs (lncRNA), micro RNAs (miRs), aging, and environmental factors. Unprecedented advances in sequencing technology, deep mutational scanning, and epigenetic and transcriptome mapping have highlighted several new areas of pathogenesis. A primary challenge with many of the ascribed changes is whether they directly cause disease or, rather, follow disease progression. Animal models have been generated to address this challenge and study new areas of arrhythmia biology, with a select few highlighted below. In the past decade, micropeptides have been identified that were previously excluded during annotations of open reading frames due to their small size or position in the transcriptome. In the heart, functionally expressed peptides include examples such as sarcolipin and dwarf open reading frame, which regulate SR Ca reuptake. Sarcolipin protein levels are decreased in humans with AF or heart failure^375^ and the sarcolipin knockout mouse developed AF with age^376^. In mice with DCM, overexpression of dwarf open reading frame attenuated the heart failure phenotype, although arrhythmias were not examined^377^. The micropeptides apelin and elabela affect cardiogenesis, fibrosis, hypertrophy, and inotropic responses. Reduced apelin levels predicted major myocardial events and MI scar size in patients with a previous MI event^377^. Apelin knockout mice developed larger scars and increased mortality following MI, although arrhythmias were not described^378^. These findings establish the pathogenic role and therapeutic potential for micropeptides in the heart, but their impact on arrhythmogenesis remains to be examined. Posttranslational modifications, primarily phosphorylation, are documented in many arrhythmias^379^. While phosphorylation by PKC, PKA, and CaMKII are the most studied mediators for changes in Na + channels^293,294,380,381^, Ca2 + channels^382–384^, K + channels^385^, NCX^386^, and RyR2^387^, conflicting data exist regarding the relative contribution of different phosphorylation sites to ion currents. Phosphomimetic and phosphoablation mouse models have been created to tease out the importance of each site to arrhythmogenesis^293,382,388,389^. In addition, posttranslational modification of signaling kinases themselves, including PKA^390^ and CaMKII^390^, play a role in arrhythmia susceptibility in response to oxidative stress. Other posttranslational modifications are less well studied, and it remains to be determined whether these cause disease or are merely epiphenomena. Epigenetic DNA modifications regulate protein expression. It is now recognized that many cardiovascular diseases are associated with epigenetic changes. For example, cardiac-specific deletion of HDAC1 and HDAC2 in mice led to dilated cardiomyopathy, arrhythmia, and premature death^391^. A family with a history of autosomal recessive DCM was later discovered to carry homozygous mutation in GATAD1^392^. In AF, epigenetics also contribute to risk as it relates to hypertension, obesity, age, and other factors^393^. Patients with persistent AF have genome-wide changes in DNA methylation^394^, and animal models could help elucidate the pathophysiology of these changes at the whole genome or protein level. MiRs regulate gene expression by RNA complementation and silencing. In the heart, miRs control cardiogenesis and pathways to affect gene expression during development. Some miRs are muscle specific and may serve as macroregulators of ion channel expression. In disease, changes in many different miRs have been documented. In dogs with rapid pacing, nicotine administration reduced miR-133 and miR-590 levels and increased AF risk^395^. Atrial tachypacing in dogs and development of AF was also associated with changes in many miRs compared with control^396^. Dogs with ventricular tachypacing developed CHF and AF and miR-29b levels declined within the first 24 h of pacing^397^. In zebrafish, miR-182 served as a TBX-5 effector and miR-182 upregulation led to block, tachycardia, and arrhythmias^398^. In mice, it was shown that miR-1 directly binds to the C-terminus of Kir2.1 and represses channel activity^399^. Moreover, a single nucleotide polymorphism in miR-1 identified from patients with AF did not suppress Kir2.1 channel activity and failed to rescue arrhythmia inducibility in miR-1 knockdown mice (whereas WT miR-1 did). Of most interest is the therapeutic potential for RNA regulation to reverse disease progression. LncRNA regulates gene expression via several mechanisms and changes in lncRNA expression have been documented in a wide range of diseases. Altered expression of lncRNA has been observed in AF^400^, including altered regulation of PITX2^401^. Another study in rabbits with AF induced by atrial tachypacing purported a mechanism whereby lncRNA “sponges” up miR-328 to regulate CACNA1C^402^. Yet another report indicated changes in lncRNA in AF reduce expression of JP2 and RYR2^403^. These studies demonstrate additional mechanisms of regulation beyond controlling gene expression and open up the field to exciting new opportunities in arrhythmia research.

## Conclusion

Antiarrhythmic drugs often work by altering conduction velocity, the length of the refractory period, or both. The properties of the Na + and Ca + 2 channels and the passive electrical properties of cardiac tissue play a role in conduction velocity. The length of the action potential and the length of the refractory period vary greatly between species due to large changes in the K + currents that essentially control repolarization. It is clear that there are species-specific differences in the variables that influence the development of arrhythmias, and it is also clear that no animal model can accurately represent a human with arrhythmias. However, the development of diagnostic and therapeutic methods for supraventricular and ventricular arrhythmias has clearly benefited from the knowledge gained from animal experiments. We hope that in the future, new insights will be gained from experiments performed at different scales, including single cells, cell cultures, prepared heart sections, isolated whole hearts, whole hearts from conscious and anesthetized animals, and systems expressing and testing functions of molecules involved in electrical excitation. The development of new diagnostic and treatment methods will result from the integration of these discoveries and not from the use of a single model or experimental approach. Electrophysiological testing should be encouraged in animals with "natural" cardiovascular disease. Animal models have played a key role in advancing our understanding of the mechanisms of human cardiac arrhythmias, but they have also revealed fundamental problems for all forms of disease modelling. In any complex process, it is better to summarize the causal path as much as possible as to empirically model the individual components. The mechanistic insights gained in the last decades illustrate the complexity of the pathogenesis of clinical arrhythmias. Models that integrate the effects of genetic and epigenetic modifiers are needed to analyze the multistep signaling pathways involved, including myocyte heterogeneity, channel processing, and downstream signalling.

For decades, animal models have been an essential tool to study arrhythmia pathophysiology and therapeutic approaches. Recent work with genetic mouse models yielded a new diagnostic tool in CRDS, identified lifesaving drug therapies for CPVT, LQT3, and ARVC, identified pathogenic mechanisms, and advanced our understanding of arrhythmogenesis in ways that other models cannot. The broad availability of transgenic mouse models and the option to generate mice with cell-specific and/or time-dependent regulation of gene expression provides a significant advantage of the mouse over other small animal species. A major limitation is that their fast heart rate, small heart size, and differential ionic currents do not fully recapitulate human cardiac electrophysiology. While guinea pigs and rabbits can overcome some of these electrophysiological limitations, genetic manipulation is limited in these species. Large animal electrophysiological and hemodynamic parameters match humans more closely but can be prohibitively expensive. Regardless, large animals are useful for preclinical studies evaluating new drugs, gene therapy, and devices that necessitate large animal models before moving to humans. Here, we provide an in-depth review of existing animal models to help interpreting published arrhythmia mechanisms as well as planning future experimental studies investigating cardiac arrhythmia diseases.

In this review, we showed how hiPSC-CMs can recapitulate arrhythmia mechanisms observed in adult CMs, such as abnormal impulse formation and conduction abnormalities, as well as disease phenotypes because of channelopathies (eg, LQT syndrome) or cardiomyopathies (eg, HCM and DCM) that trigger arrhythmic behaviors. However, it can be argued that thus far they have provided only limited new insights into arrhythmia mechanism.

In summary, animal models remain of crucial importance for the discovery and development of cardiac antiarrhythmic drugs. These models help evaluate drug candidates, predict therapeutic efficacy and safety, and better understand the underlying processes of arrhythmogenesis. Future research on effective therapies to prevent and treat cardiac arrhythmias is promising as animal models are improved and more commonly used in conjunction with complementary methods.

## Data Availability

The data that support the findings of this study are available from the corresponding author upon reasonable request.

## Abbreviations

AF: Atrial fibrillation
AP: Action potential
APD: Action potential duration
ARVC: Arrhythmogenic right ventricular cardiomyopathy
AT: Atrial tachycardia
BrS: Brugada syndrome
CHB: Complete heart block
CPVT: Catecholaminergic polymorphic ventricular tachycardia
CRDS: Ca^2+^ release deficiency syndrome
DAD: Delayed afterdepolarization
DCM: Dilated cardiomyopathy
DSC2: Desmocollin-2
DSG2: Desmoglein-2
EAD: Early afterdepolarization
ECM: Extracellular matrix
HCM: Hypertrophic cardiomyopathy
LQTS: Long QT syndrome
MAT: Multifocal atrial tachycardia
MPO: Myeloperoxidase
PES: Programmed electrical stimulation
PKP2: Plakophilin-2
PLB: Phospholamban
PVC: Premature ventricular contraction
SCD: Sudden cardiac death
SQTS: Short QT syndrome
SR: Sarcoplasmic reticulum
SVT: Supraventricular tachycardia
TAC: Transverse aortic constriction
TdP: Torsades de Pointes
TGF: Transforming growth factor
VF: Ventricular fibrillation
VT: Ventricular tachycardia
